# Mutated *FGFR1* is an oncogenic driver and therapeutic target in high-risk neuroblastoma

**DOI:** 10.1172/JCI189152

**Published:** 2026-02-12

**Authors:** Lisa Werr, Jana Boland, Josephine Petersen, Fiorella Iglesias, Stefanie Höppner, Christoph Bartenhagen, Carolina Rosswog, Anna-Maria Hellmann, Yvonne Kahlert, Nadine Hemstedt, Nadliv Ibruli, Marcel A. Dammert, Boris Decarolis, Jan-Michael Werner, Florian Malchers, Kathrin Schramm, Olaf Witt, Klaus H. Beiske, Anne Gro Wesenberg Rognlien, Maria Winther Gunnes, Karin P. Langenberg, Jan Molenaar, Marie Bernkopf, Sabine Taschner-Mandl, Debbie Hughes, Sally L. George, Louis Chesler, Johannes H. Schulte, Giuseppe Barone, Mario Capasso, Lea F. Surrey, Rochelle Bagatell, Julien Masliah-Planchon, Gudrun Schleiermacher, Holger Grüll, Frank Westermann, Anne M. Schultheis, Reinhard Büttner, Anton G. Henssen, Angelika Eggert, Martin Peifer, Neerav N. Shukla, Thorsten Simon, Barbara Hero, H. Christian Reinhardt, Roman K. Thomas, Matthias Fischer

**Affiliations:** 1 Department of Experimental Pediatric Oncology and Hematology,; 2 Center for Molecular Medicine Cologne and Department of Experimental Pediatric Oncology, Medical Faculty, and; 3 Department of Translational Genomics, Medical Faculty, University of Cologne, Cologne, Germany.; 4 Mildred Scheel School of Oncology, Cologne, University Hospital Cologne, Medical Faculty, Cologne, Germany.; 5 Koeln Fortune Program/Faculty of Medicine, University of Cologne, Cologne, Germany.; 6 Department of Pediatrics, Memorial Sloan Kettering Cancer Center, New York, New York, USA.; 7 Else Kröner Forschungskolleg Clonal Evolution in Cancer, University Hospital Cologne, Cologne, Germany.; 8 Institute of Pathology, Faculty of Medicine and University Hospital Cologne,; 9 Department of Pediatric Oncology and Hematology, and; 10 Department of Neurology, Faculty of Medicine and University Hospital Cologne, University of Cologne, Cologne, Germany.; 11 Division of Oncology and Christian Doppler Laboratory for Personalized Immunotherapy, Department of Medicine I, Medical University of Vienna, Vienna, Austria.; 12 Institute of Biochemistry I, Medical Faculty, University of Cologne, Cologne, Germany.; 13 Hopp Children’s Cancer Center Heidelberg, Heidelberg, Germany.; 14 Division of Pediatric Glioma Research (B360) and; 15 Clinical Cooperation Unit Pediatric Oncology, German Cancer Research Center (DKFZ), Heidelberg, Germany.; 16 Department of Pediatric Oncology, Hematology, Immunology and Pulmonology, Heidelberg University Hospital, Heidelberg, Germany.; 17 German Cancer Consortium (DKTK), Heidelberg, Germany.; 18 National Center for Tumor Diseases, Heidelberg, Germany.; 19 Department of Pathology, Oslo University Hospital, Oslo, Norway.; 20 Division of Pediatric and Adolescent Medicine, Oslo University Hospital Rikshospitalet, Oslo, Norway.; 21 Princess Máxima Center for Pediatric Oncology, Heidelberglaan 25, Utrecht, Netherlands.; 22 Department of Pharmaceutical Sciences, University Utrecht, Utrecht, Netherlands.; 23 St. Anna Children’s Cancer Research Institute, Vienna, Austria.; 24 Division of Clinical Studies, The Institute of Cancer Research, Sutton, London, United Kingdom.; 25 The Francis Crick Institute, London, United Kingdom.; 26 Children and Young People’s Unit, The Royal Marsden Hospital, Sutton, London, United Kingdom.; 27 University Children’s Hospital, Eberhard Karls University, Abteilung I, Tuebingen, Germany.; 28 Great Ormond Street Hospital, London, United Kingdom.; 29 Department of Molecular Medicine and Medical Biotechnology, University of Naples “Federico II,” Naples, Italy.; 30 CEINGE Biotecnologie Avanzate, Napoli, Italy.; 31 Division of Genomic Diagnostics, Children’s Hospital of Philadelphia, Philadelphia, Pennsylvania, USA.; 32 Department of Pathology and Laboratory Medicine, and; 33 Department of Pediatrics, Division of Oncology, Children’s Hospital of Philadelphia and the Perelman School of Medicine, University of Pennsylvania, Philadelphia, Pennsylvania, USA.; 34 Oncogenetic lab, Institute Curie, Paris, France.; 35 Recherche Translationelle en Oncologie Pédiatrique; INSERM U830, and SIREDO Integrated Pediatric Oncology Center, PSL Research University, Institut Curie, Paris, France.; 36 Faculty of Medicine and University Hospital of Cologne, Institute of Diagnostic and Interventional Radiology, University of Cologne, Cologne, Germany.; 37 Division of Neuroblastoma Genomics, DFKZ, Heidelberg, Germany.; 38 Department of Pediatric Oncology/Hematology, Charité-Universitätsmedizin Berlin, Berlin, Germany.; 39 DKTK, Partner Site Berlin, and DKFZ, Heidelberg, Germany.; 40 Max-Delbrück-Center for Molecular Medicine in the Helmholtz Association (MDC), Technology Platform Electron Microscopy, Berlin, Germany.; 41 Experimental and Clinical Research Center of the MDC and Charité Berlin, Berlin, Germany.; 42 Department of Hematology and Stem Cell Transplantation, University Hospital Essen, University Duisburg-Essen, DKTK, partner site Essen, Essen, Germany.

**Keywords:** Cell biology, Oncology, Cancer, Mouse models

## Abstract

Fibroblast growth factor receptor 1 (*FGFR1*) is recurrently mutated at p.N546 in neuroblastoma. We examined whether mutant FGFR1 is an oncogenic driver, a predictive biomarker, and an actionable vulnerability in this malignancy. *FGFR1* mutations at p.N546 were associated with high-risk disease and rapid tumor progression, resulting in dismal outcome for these patients. Ectopic expression of *FGFR1^N546K^* induced constitutive downstream signaling and IL-3–independent growth in Ba/F3 cells, indicating oncogene-addicted proliferation. In *FGFR1^N546K^*;*MYCN* transgenic mice, neuroblastoma developed within the first days of life, with fatal outcome within 3 weeks, reflecting the devastating clinical phenotypes of patients with *FGFR1*-mutant, high-risk neuroblastoma. Treatment with FGFR inhibitors impaired proliferation and pathway activation in *FGFR1^N546K^*-expressing Ba/F3 and patient-derived *FGFR1^N546K^*-mutant neuroblastoma cells and inhibited tumor growth in *FGFR1^N546K^*;*MYCN* transgenic mice and in a chemotherapy-resistant, patient-derived xenograft mouse model. In addition, partial regression of *FGFR1^N546K^*-mutant tumor lesions occurred upon treatment with the FGFR inhibitor futibatinib and low-intensity chemotherapy in a patient with refractory neuroblastoma. Together, our data demonstrate that FGFR1^N546K^ is a strong oncogenic driver in neuroblastoma associated with failure of current standard chemotherapy and suggest potential clinical benefit of FGFR-directed therapies in patients with high-risk mutant *FGFR1*.

## Abstract 2

*FGFR1^N546K^* is a strong oncogenic driver in neuroblastoma and targeting mutated FGFR1 with FGFR inhibitors may be a promising therapeutic strategy in high-risk neuroblastoma patients.

## Introduction

 Neuroblastoma is a malignant pediatric tumor of the developing sympathetic nervous system, representing 8% of childhood malignancies (1). Roughly 50% of patients have excellent outcome with no or limited treatment, owing to frequent occurrence of spontaneous regression or maturation of the tumor into benign ganglioneuroma (2, 3). By contrast, the 50% of patients is at high risk to die from disease, with long-term survival still being below 50% despite intense multimodal treatment strategies (4, 5). According to the International Neuroblastoma Risk Group (INRG), patients are classified as high risk if they have metastatic disease and are older than 18 months at diagnosis, or if their tumor cells bear genomic amplification of the proto-oncogene *MYCN* (6, 7). On the molecular level, high-risk tumors are defined by the presence of telomere maintenance mechanisms, which are invariably absent in low-risk neuroblastoma (8, 9). In addition, mutations in genes related to RAS/MAPK pathway activation, such as the RAS family genes themselves or anaplastic lymphoma kinase (*ALK*), are associated with inferior outcome when occurring in combination with telomere maintenance mechanisms (10). In patients with relapsed or refractory *ALK*-mutated neuroblastoma, ALK inhibitors (e.g., crizotinib, lorlatinib, ceritinib) have shown promising antitumor activity (11–13), and their potential therapeutic value in first-line therapy is being evaluated (4). However, targeted treatment options for patients with tumors bearing alterations in genes other than *ALK* are still limited.

We and others have found that mutations at codon 546 of the fibroblast growth factor receptor 1 (*FGFR1*) gene, affecting the tyrosine kinase domain, recur in high-risk neuroblastoma (10, 14–20), and that their presence tends to be associated with worse survival (15). The impact of mutant *FGFR1* on neuroblastoma pathogenesis, however, has remained unclear (15, 20). FGFR1 belongs to the family of FGFR transmembrane receptor tyrosine kinases consisting of 2 or 3 extracellular domains, 1 transmembrane domain, and 2 tyrosine kinase subdomains (21–23). Binding of FGF ligands activates various downstream pathways, such as RAS/MAPK, PI3K/AKT, and STAT signaling, thereby regulating distinct cellular processes, including proliferation, differentiation, survival, and migration. Dysregulation of FGFR signaling by point mutations, amplification, or translocations of *FGFR* genes contributes to tumorigenesis in various solid tumors (21), such as lung cancer, breast cancer, bladder carcinoma, cholangiocarcinoma, and glioblastoma (24, 25). Several selective FGFR inhibitors have been developed, therefore, and entered clinical trials in recent years, and erdafitinib, pemigatinib, infigratinib, and futibatinib have been approved by the FDA for adult patients with cancer with FGFR-altered tumors.

Here, we set out to determine the association of mutant *FGFR1* with clinical neuroblastoma phenotypes, its transforming capacity in vitro, and its potential role as an oncogenic driver in neuroblastoma pathogenesis in vivo. We also evaluated whether mutated *FGFR1* may represent an actionable alteration in neuroblastoma, both experimentally in vitro and in vivo, and in a patient with relapsed disease, to develop more efficacious treatment options in children with this deadly malignancy.

## Results

### Patients with FGFR1^N546^-mutated neuroblastoma have poor outcome.

**Figure 1:**
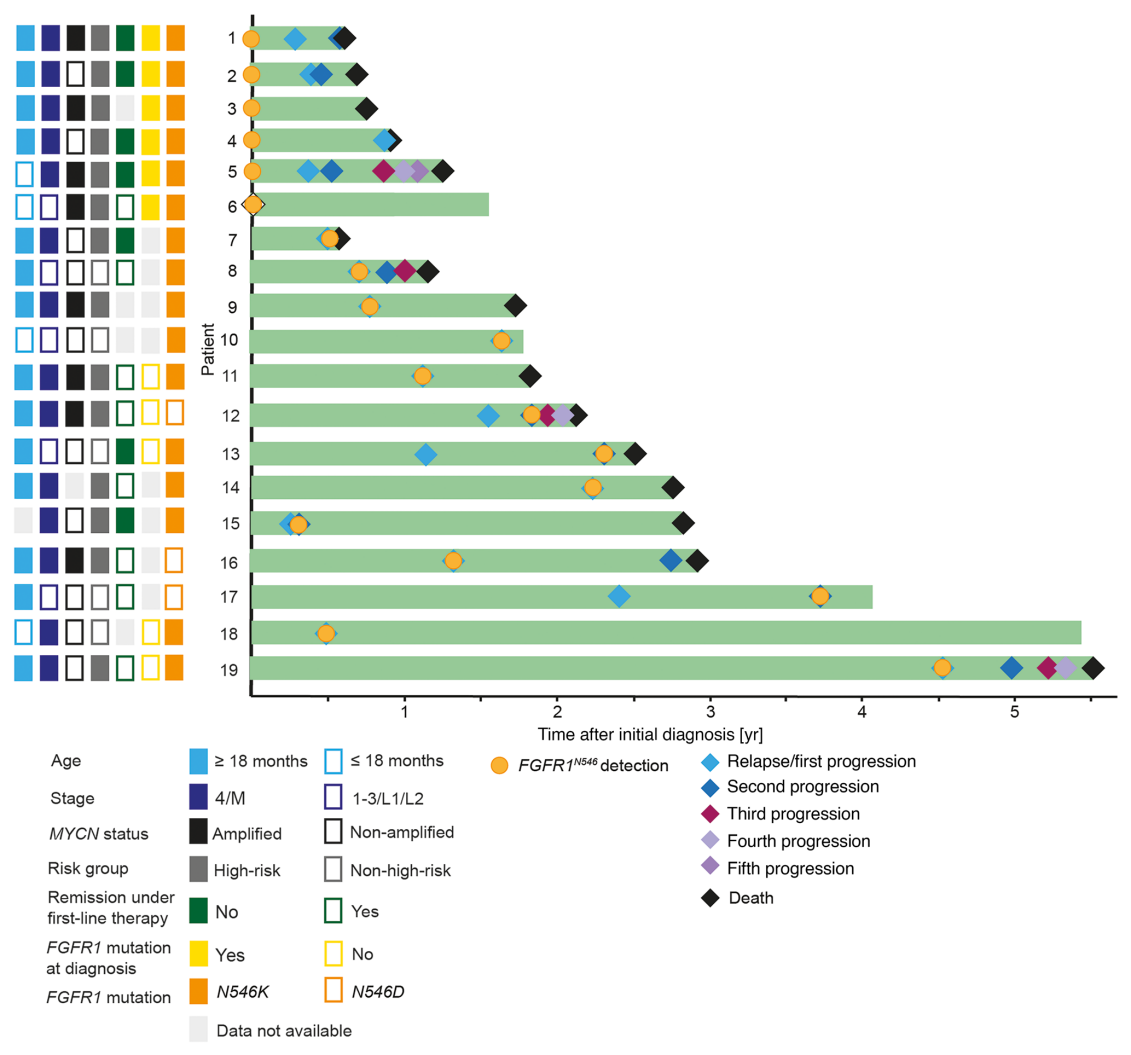
Clinical courses of patients with *FGFR1^N546^* mutated neuroblastoma. Swimmer plot illustrating the course of disease of 19 patients with *FGFR1^N546^*-mutated neuroblastoma. Light grey rectangles indicate missing data (not available; NA).

**Figure 2:**
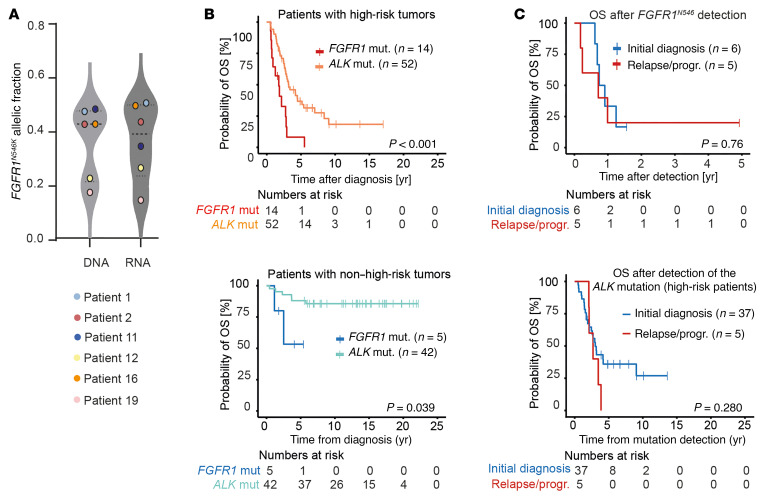
Patients with *FGFR1^N546^*-mutated neuroblastoma have a poor outcome. (**A**) Allelic fractions of *FGFR1^N546^* mutations (mut.) in DNA and RNA-Seq data obtained from 6 of the *FGFR1^N546^-*mutated tumors. (**B**) OS of patients at high risk (top) and those not at high risk (bottom) with *FGFR1^N546^***-**mutated neuroblastoma and patients with tumors bearing single nucleotide variants in the *ALK* kinase domain (27). Survival curves were estimated according to the Kaplan-Meier test and compared with log-rank test. (**C**) OS after first-time detection of mutant *FGFR1^N546^* (top) or mutant *ALK* in high-risk patients (bottom) at diagnosis (blue) or relapse/progression (progr.) (red). Survival curves were estimated according to Kaplan-Meier and compared with a log-rank test.

To determine the association of *FGFR1^N546^* mutations with clinical variables and outcome in neuroblastoma, we screened sequencing data obtained from patients in Germany, the Netherlands, Austria, Norway, the United Kingdom, France, Italy, and the United States, and identified mutations at this position in 19 cases (Figure 1 and Supplemental Figure 1; supplemental material available online with this article; https://doi.org/10.1172/JCI189152DS1). The prevalence of *FGFR1^N546^* mutations was approximately 1% at diagnosis (*n* = 2 of 239 patients in the German cohort) and 2% at relapse (*n* = 2 of 73 patients in the German cohort). Mutations led to asparagine-to-lysine substitution in 16 of the tumors (p.N546K) and asparagine-to-aspartic acid substitution in 3 cases (p.N546D), both predicted to be activating (Supplemental Figure 2A and Supplemental Table 1) (15, 26). Mutations at other positions of *FGFR1* were not found in the cohort from which we had complete sequencing information available (*n* = 312). We also observed that the mutated *FGFR1^N546^* allele was expressed at the RNA level in affected tumors (*n* = 6 of 19 tumors analyzed) (Figure 2A). *FGFR1^N546^* mutations were found predominantly in International Neuroblastoma Staging System (INSS) stage 4/INRG Staging System stage M tumors and high-risk disease, as defined by INRG (*n* = 14 of 19 cases), and occurred in combination with *MYCN* amplification in 8 of 18 tumors (not analyzed, *n* = 1) (Supplemental Table 1).

In 6 patients, the *FGFR1^N546^* variant was detected at the time of diagnosis, whereas it occurred only at relapse or progression in 5 other cases (not analyzed at diagnosis, *n* = 8) (Figure 1 and Supplemental Table 1). Survival of patients with *FGFR1^N546^*-mutant high-risk tumors was poor: all but 1 of these patients have died (2-year overall survival [OS] = 0.408 ± 0.136, and survival was significantly inferior to that of patients with tumors bearing mutant *ALK*, the most frequently altered receptor tyrosine kinase in this malignancy (Figure 2B) (27). The prevalence of the risk factors age, stage, and *MYCN* status did not differ between *FGFR1^N546^*- and *ALK*-mutant high-risk neuroblastoma (Supplemental Figure 2B).

Survival of patients with *FGFR1^N546^*-mutant non–high-risk disease appeared to be better than that of high-risk patients (2-year OS = 0.800±0.179; *P* = 0.304), but inferior to that of *ALK*-mutant non–high-risk patients (Figure 2B); however, this finding was limited by the small number of non–high-risk patients with mutant *FGFR1* and the short follow-up of some of these patients. The difference in survival between patients with *FGFR1*- and *ALK*-mutant neuroblastoma was also evident after exclusion of patients with *ALK*-mutant tumors who had been treated with ALK inhibitors (Supplemental Figure 2C). Most of the patients with *FGFR1^N546^*-mutant neuroblastoma had rapid disease progression shortly after detection of the mutation despite therapy (Supplemental Figure 1); median survival was 270 (range, 66–1,798) days after detection of mutated *FGFR1^N546^*, and this did not differ significantly between patients in whom mutations occurred in tumors at diagnosis or only at relapse (1-year OS = 0.333±0.192 vs. 0.200±0.179, respectively; *P* = 0.76) (Figure 2C). Similarly, survival did not differ between high-risk patients with *ALK* mutation in whom mutations occurred at diagnosis or only at relapse (*P* = 0.280) (Figure 2C), which was, however, significantly longer than that of patients with *FGFR1*-mutant neuroblastoma after detection of mutated *FGFR1* (median survival 1,108 days vs. 270 days, respectively; *P* < 0.001). Together, these data suggest *FGFR^N546^* mutations contribute to chemotherapy resistance and poor outcome in neuroblastoma.

### FGFR1^N546K^ is an oncogenic driver in Ba/F3 cells.

**Figure 3:**
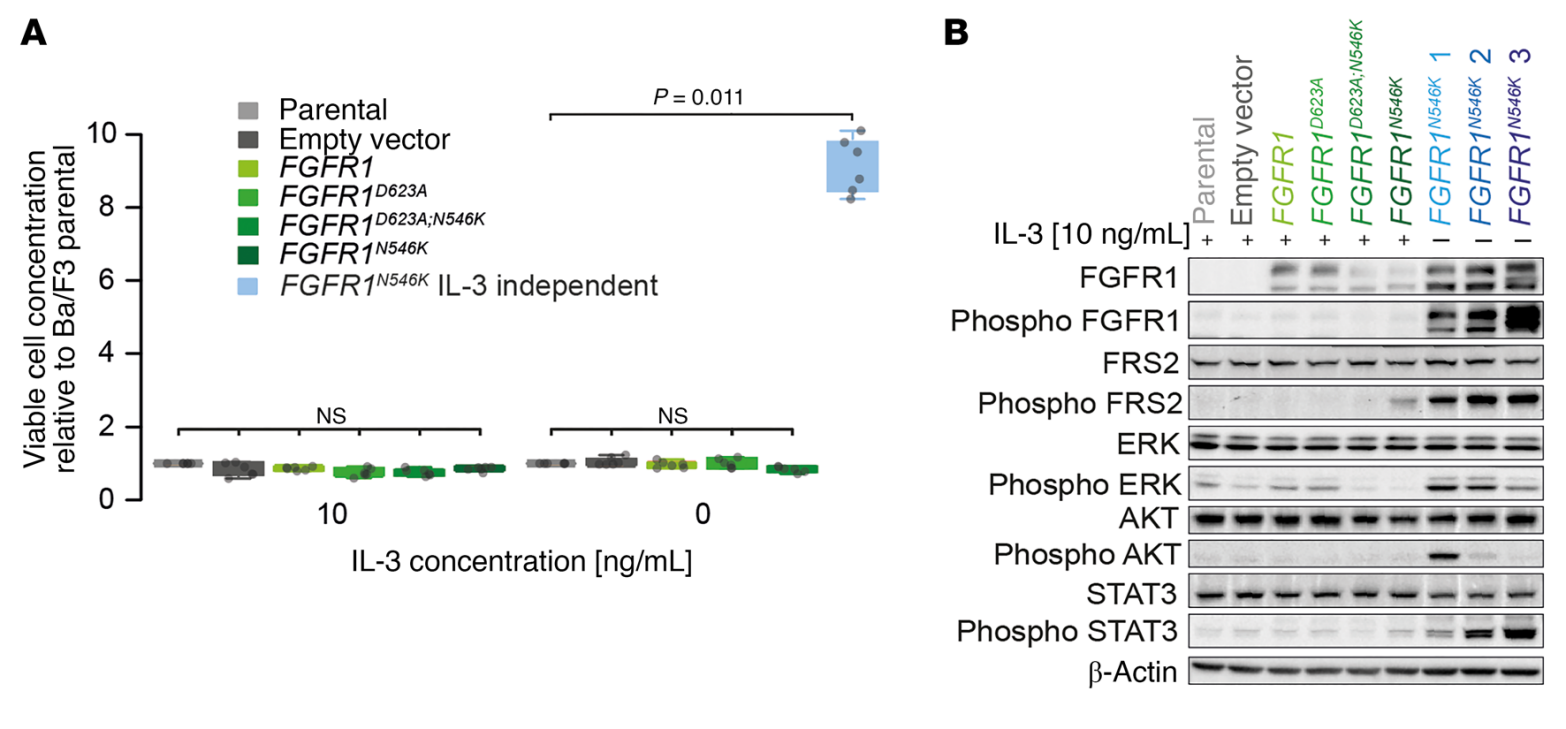
*FGFR1^N546K^* drives cytokine-independent proliferation of Ba/F3 cells and renders them sensitive to pharmacological FGFR inhibition. (**A**) Relative cell counts of viable Ba/F3 cells transduced with different vectors (empty vector, *FGFR1* WT, *FGFR1^D623A^*, *FGFR1^D623A;N546K^*, *FGFR1^N546K^*) normalized to parental Ba/F3 cells. Analyses were performed with 10 ng/mL IL-3 in culture medium and after withdrawal of IL-3 after 144 hours. Average numbers (±SD) of 6 independent experiments are shown. *P* values were calculated in pair-wise comparisons to Ba/F3 parental cells using a 1-sided Wilcoxon rank-sum test and adjusted for multiple testing using the Benjamini–Hochberg method. (**B**) Levels of total and phosphorylated proteins of the FGFR pathway in IL-3–dependent Ba/F3 cells (parental, empty vector, *FGFR1* WT, *FGFR1^D623A^*, *FGFR1^D623A;N546K^*, *FGFR1^N546K^*) and 3 IL-3–independent *FGFR1^N546K^***-**transduced Ba/F3 cell clones (blue). IL-3–independent *FGFR1^N546K^***-**transduced BA/F3 cells shown in different blue tones correspond to cells from 3 independent experiments labelled in light blue in (**A**). Antibodies against total and phosphorylated FGFR1, the adaptor protein FRS2, and the downstream targets AKT, ERK, and STAT3 were used. β-actin served as loading control.

Previous studies have shown that the p.N546K variant leads to enhanced tyrosine kinase activity of FGFR1 in Rat-1 and neuroblastoma cells, and that *FGFR1^N546K^* may confer oncogenic properties in vitro (20, 26). To validate the transforming capacity of mutant *FGFR1^N546K^*, we generated Ba/F3 cells stably expressing *FGFR1^WT^* and *FGFR1^N546K^*. We also generated Ba/F3 cells with stable expression of *FGFR1* bearing a kinase-dead mutation (*FGFR1^D623A^*), both in the *FGFR1^WT^* and *FGFR1^N546K^* background, to test whether *FGFR1^N546K^* leads to constitutive activation of the kinase domain of FGFR1 (26). Because proliferation of parental Ba/F3 cells is dependent on IL-3 (28, 29), we evaluated the transforming capacity of mutated *FGFR1* by determining cell counts in its presence and absence. As expected, cell counts did not differ between *FGFR1^N546K^*-transduced and control Ba/F3 cells in the presence of IL-3. By contrast, we found a significantly higher number of viable Ba/F3 *FGFR1^N546K^* cells in comparison with all control cells after withdrawal of IL-3 (Figure 3A), which points toward the transforming potential of this variant. Of note, proliferation of *FGFR1^N546K^*-expressing Ba/F3 cells was completely abrogated by introduction of the kinase-dead mutation p.D623A (Figure 3A), indicating that activation of the kinase-domain is essential for the oncogenic effect.

We next generated 3 distinct IL-3–independent, *FGFR1^N546K^*-expressing Ba/F3 cell lines and analyzed FGFR pathway activation, using immunoblot analysis of downstream targets. Protein expression of FGFR1 was detected in all *FGFR1*-transduced Ba/F3 cells, whereas significant levels of FGFR1 phosphorylation occurred only in the 3 IL-3–independent *FGFR1^N546K^* cell lines (Figure 3B). Accordingly, we found intense phosphorylation of the downstream targets FRS2, ERK, AKT, and STAT3 in IL-3–independent *FGFR1^N546K^* cells, whereas phosphorylation of these proteins was substantially lower in all cytokine-dependent cells (Figure 3B). Together, these data indicate the variant p.N546K constitutively activates the tyrosine kinase domain of FGFR1, leading to induction of FGFR signaling by phosphorylation of downstream targets and consecutive autonomous cell proliferation, which is in line with previous reports (20, 26).

### Treatment of FGFR1^N546K^ Ba/F3 cells with FGFR inhibitors downregulates FGFR pathway activity and impairs cell proliferation.

**Figure 4:**
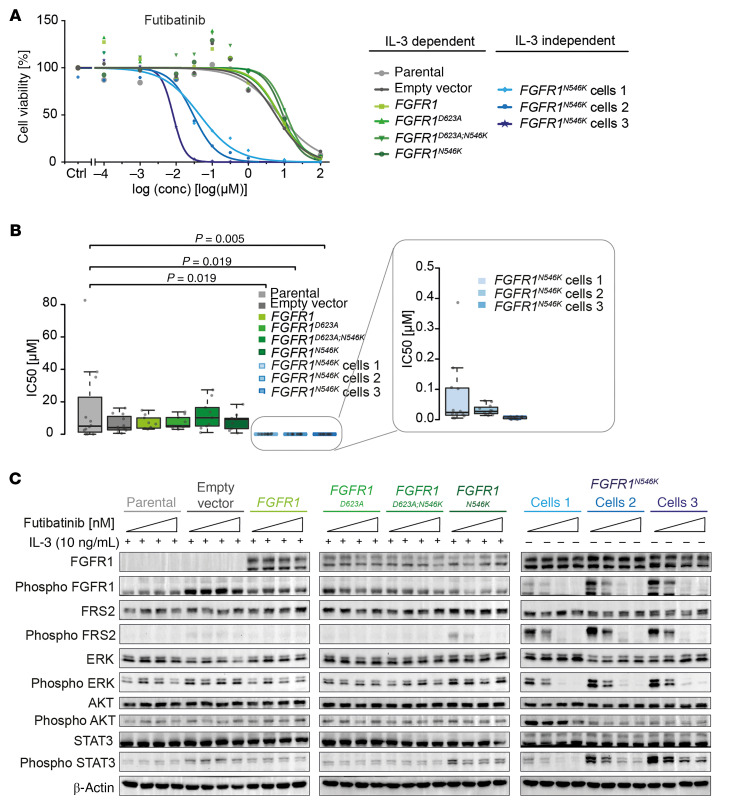
*FGFR1^N546K^* renders Ba/F3 cells sensitive to pharmacological FGFR inhibition. (**A**) Relative cell viability of IL-3–dependent Ba/F3 cells (parental, empty vector, *FGFR1* WT, *FGFR1^D623A^*, *FGFR1^D623A;N546K^*, *FGFR1^N546K^*) and 3 IL-3–independent *FGFR1^N546K^***-**transduced Ba/F3 cell clones (blue) after treatment with various concentrations (conc.) of futibatinib (in μM: 0.0001, 0.001, 0.01, 0.0398, 0.0631, 0.1, 1, 10, and 100) and DMSO as control for 72 hours. Mean cell viabilities ± SD of 3 (*FGFR1* WT, *FGFR1^D623A^*, *FGFR1^D623A;N546K^*, *FGFR1^N546K^*) or 4 (parental, empty vector, *FGFR1^N546K^* IL-3–independent cells) independent experiments conducted in triplicate each are plotted. (**B**) IC_50_ of futibatinib in IL-3–dependent Ba/F3 cells and 3 IL-3–independent *FGFR1^N546K^*-transduced Ba/F3 cell clones, derived from 3 (*FGFR1* WT, *FGFR1^D623A^*, *FGFR1^D623A;N546K^*, *FGFR1^N546K^*) or 4 (parental, empty vector, *FGFR1^N546K^* IL-3–independent cells) independent experiments consisting of triplicates each. *P* values were calculated in pair-wise comparisons to Ba/F3 parental cells using a 1-sided Wilcoxon rank-sum test and adjusted for multiple testing using the Benjamini–Hochberg method. (**C**) Levels of total and phosphorylated proteins of the FGFR pathway in IL-3–dependent Ba/F3 cells (parental, empty vector, *FGFR1* WT, *FGFR1^D623A^*, *FGFR1^D623A;N546K^*, *FGFR1^N546K^*) and 3 IL-3–independent *FGFR1^N546K^* transduced Ba/F3 cell clones (blue) after treatment with DMSO or 10, 50, or 100 nM futibatinib (experiment was conducted 3 times).

Because various small-molecule FGFR inhibitors have been developed and are in clinical use for adult patients with tumors bearing *FGFR* alterations, we next asked whether FGFR1^N546K^ is a potential therapeutic target. We examined effects of the clinically approved FGFR inhibitors futibatinib and erdafitinib in IL-3–independent *FGFR1^N546K^* and control Ba/F3 cells (30–32). Cell viability of IL-3–independent *FGFR1^N546K^*-expressing cells was significantly reduced in comparison with parental cells after treatment with futibatinib or erdafitinib for 72 hours, whereas no effect was observed in IL-3–dependent cells bearing *FGFR1^N546K^*, WT *FGFR1*, or *FGFR1^D623A^* in the presence or absence of p.N546K (Figure 4, A and B, and Supplemental Figure 3, A–C). In line with these observations, we found that phosphorylation of FGFR1 and its downstream targets decreased in IL-3–independent *FGFR1^N546K^* cells upon treatment with futibatinib or erdafitinib in a dose-dependent manner (Figure 4C and Supplemental Figure 3D).

### Targeted FGFR1^N546K^ expression leads to neuroblastoma development in vivo.

To determine whether *FGFR1^N546K^* drives the development of neuroblastoma, we generated an *R26-LSL-FGFR1^N546K^* transgenic mouse model in which an *FGFR1^N546K^* transgene is integrated into the *ROSA26* locus and expressed after *Cre-loxP*-mediated recombination (Supplemental Figure 4A). To direct expression of the transgene to cells of the developing sympathetic nervous system, *FGFR1^N546K^* transgenic mice were crossbred with mice bearing a *Th-IRES-Cre* transgene, which express *Cre* recombinase under control of the tyrosine hydroxylase (*Th*) promoter (33). In *R26-LSL-FGFR1^N546K/wt^;Th-IRES-Cre^tg/wt^* mice, the stop cassette flanked by *loxP* recombination sites is removed in cells of the neural crest during early development, leading to ectopic *FGFR1^N546K^* expression driven by the *CAG* promoter (Supplemental Figure 4A).

**Figure 5:**
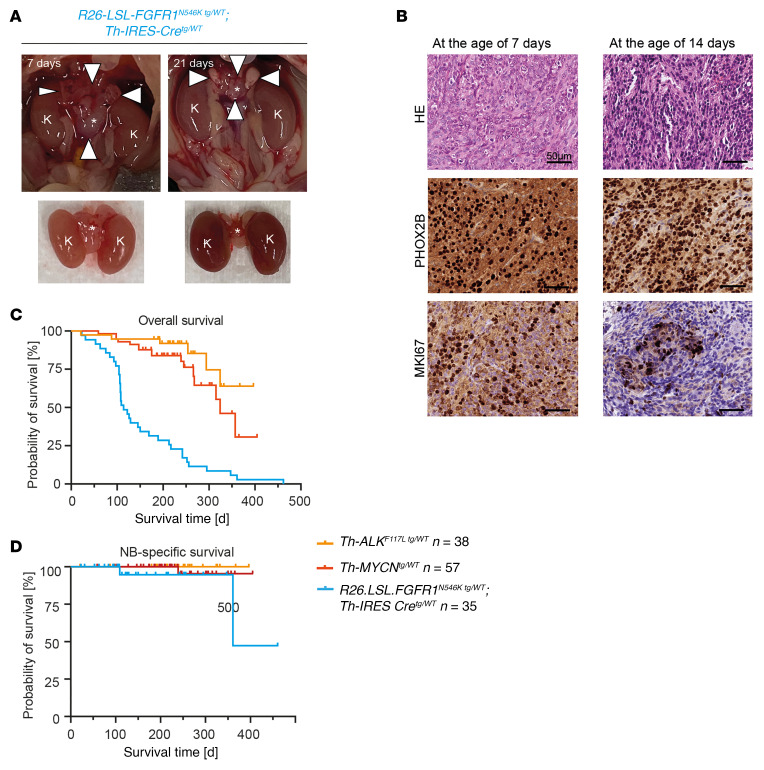
*FGFR1^N546K^* drives neuroblastoma development in a transgenic mouse model. (**A**) Images of abdominal cavities of *R26-LSL-FGFR1^N546K/wt^;Th-IRES-Cre^tg/wt^* mice sacrificed at the ages of 7 and 21 days, showing tumors between the kidneys (top). Arrows point to the tumor boundaries. Kidneys (K) and tumors (indicated by asterisks) were prepared for better visualization of the tumors (bottom). (**B**) H&E and PHOX2B and Ki67 immunohistochemical staining of tumor sections obtained from *R26-LSL-FGFR1^N546K/wt^;Th-IRES-Cre^tg/wt^* mice at the ages of 7 (left) and 14 (right) days; scale bar: 50 μm. (**C** and **D**) OS (**C**) and neuroblastoma-specific (NB-specific) survival (**D**) of *R26-LSL-FGFR1^N546K/wt^;Th-IRES-Cre^tg/wt^* (blue), *Th-MYCN^tg/wt^* (red), and *Th-ALK^F1174L/wt^* (yellow) mice. Although OS was reduced in *FGFR1^N546K^* transgenic mice in comparison with the other groups, due to development of papillomas and sarcomas (*FGFR1^N546K^* vs. *MYCN^tg^*, *P* < 0.001; *FGFR1^N546K^* vs. *ALK^F1174L^*, *P* < 0.001; *MYCN^tg^* vs. *ALK^F1174L^*, *P* = 0.106), neuroblastoma-specific survival of *FGFR1^N546K^* transgenic mice was worse only in comparison with *ALK^F1174L^* mice (*P* = 0.049), but did not differ from that of *MYCN^tg^* mice (*P* = 0.226). Survival of *ALK^F1174L^* compared with *MYCN^tg^* mice was not significant (*P* = 0.394). Survival curves were estimated according to Kaplan-Meier test and compared with a log-rank test.

Macroscopic inspection of the abdominal cavity in 16 *R26-LSL-FGFR1^N546K/wt^;Th-IRES-Cre^tg/wt^* mice sacrificed within the first days of life revealed neuroblastoma formation in all cases, which was validated by histological examination (Figure 5, A and B, and Supplemental Table 2). By contrast, no abdominal tumors were detected in 35 *R26-LSL-FGFR1^N546K/wt^;Th-IRES-Cre^tg/wt^* mice at the age of 4 weeks by MRI, which suggested early *FGFR1^N546K^*-driven neuroblastomas do not develop an aggressive phenotype but undergo spontaneous regression within the first weeks after birth (Figure 5, A and B). Two of these mice developed neuroblastoma beyond the age of 4 weeks, as detected by MRI (Supplemental Table 3). In 1 mouse, the tumor partially regressed over time, whereas in the other, disease progressed to fatal outcome (Supplemental Figure 4, B and C). Histological assessment and expression of paired-like homeobox 2B (PHOX2B) confirmed that both tumors were neuroblastoma, and FGFR1 expression was validated by immunofluorescence (Supplemental Figure 4, D and E).

By contrast, absence of neuroblastoma was validated by macroscopic inspection of the abdominal cavity in the remaining 33 animals (Supplemental Table 3). We noted, however, that adrenal glands of *R26-FGFR1-N546K^N546K/wt^;Th-IRES-Cre^tg/wt^* mice without visible tumors that were sacrificed at the age of 13–21 weeks contained microscopic clusters of neuroblasts in 4 of 9 cases (Supplemental Figure 5A, Supplemental Table 3), resembling neuroblastoma in situ (34). In addition, we noted that long-term survival of *R26-FGFR1^N546K/wt^;Th-IRES-Cre^tg/wt^* mice was limited by the development of papillomas at the tail, mouth, and genitals, as well as sarcomas (Figure 5C, Supplemental Figure 5, B–F, and Supplemental Table 3). The development of these tumors is likely due to low-level expression of tyrosine hydroxylase in murine skin and muscle (35, 36), leading to expression of the *FGFR1^N546K^* transgene also in these tissues and consecutive development of papillomas and sarcomas.

We next compared the phenotype of *R26-FGFR1^N546K/wt^;Th-IRES-Cre^tg/wt^* mice with that of mice transgenic for *ALK^F1174L^*, representing 1 of the most common tyrosine receptor kinase mutations in human neuroblastoma (14, 27, 37). In contrast to *FGFR1^N546K^* transgenic mice, we did not detect any tumors in *Th-ALK^F1174L^* mice beyond the age of 4 weeks by MRI imaging or by macroscopic or histological inspection of the adrenal glands (Supplemental Figure 6A). We also did not observe macroscopic tumors in the adrenal glands of *Th-ALK^F1174L^* mice sacrificed at the age of 14 days (*n* = 3 mice) (Supplemental Figure 6B); however, histological examination revealed neuroblastoma in situ in 2 of 4 adrenal glands in these animals, similar to the findings in older *FGFR1^N546K^* transgenic mice (Supplemental Figure 6C).

### Co-expression of FGFR1^N546K^ and MYCN drives aggressive disease in murine neuroblastoma.

**Figure 6:**
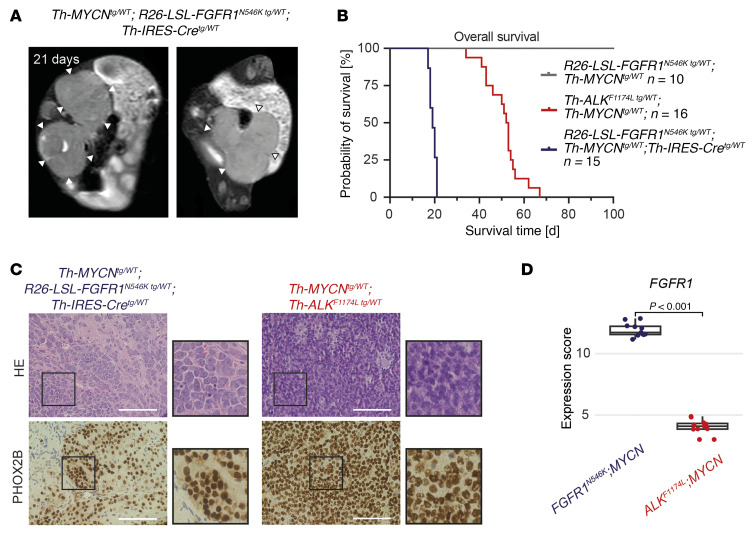
Concurrent expression of *FGFR1^N546K^* and *MYCN* promotes aggressive neuroblastoma in murine models. (**A**) Axial T2-weighted MRI scans of an *R26-FGFR1^N546K/wt^;Th-IRES-Cre^tg/wt^;Th-MYCN^tg/wt^* mouse that developed neuroblastomas at the adrenal glands (left) and the pelvic sympathetic trunk (right). (**B**) OS of *R26-FGFR1^N546K/wt^;Th-IRES-Cre^tg/wt^;Th-MYCN^tg/wt^*, Th-*ALK^F1174L/wt^*;*Th-MYCN^tg/wt^*, and *R26-LSL-FGFR1^N546K/wt^;Th-MYCN^tg/wt^* mice. Survival of all groups differed significantly from each other in pairwise comparisons (*P* < 0.001 each). Survival curves were estimated according to Kaplan-Meier tests and compared with log-rank tests. (**C**) H&E (top) and PHOX2B immunohistochemical (bottom) staining of an adrenal tumor obtained from an *R26-FGFR1^N546K/wt^;Th-IRES-Cre^tg/wt^;Th-MYCN^tg/wt^* mouse at the age of 21 days, and of an adrenal tumor obtained from a *Th-ALK^F1174L/wt^;Th-MYCN^tg/wt^* mouse at the age of 49 days; scale bar: 50 μm. (**D**) *FGFR1* transcript levels in tumors obtained from *R26-FGFR1^N546K/wt^;Th-IRES-Cre^tg/wt^;Th-MYCN^tg/wt^* mice and from *Th-ALK^F1174L/wt^;Th-MYCN^tg/wt^* mice (*n* = 11 each), as determined by RNA-Seq. Box plots show the median, first, and third quartiles; the whiskers represent the minimum and maximum values within ±1.5 times the interquartile range. Comparisons between groups were performed using a 2-tailed Wilcoxon rank-sum test.

Because *FGFR1* mutations occurred in combination with *MYCN* amplification in almost half of the tumors of patients, we sought to reproduce this genotype in a murine model. We mated *R26-FGFR1-N546K^N546K/wt^;Th-IRES-Cre^tg/wt^* mice with an established *Th-MYCN* neuroblastoma mouse model (38) to assess the effect of *FGFR1^N546K^* and *MYCN* co-expression on tumor development and progression. We found that ectopic co-expression of *FGFR1^N546K^* and *MYCN* resulted in development of multifocal abdominal tumors within the first days of life with 100% penetrance (Figure 6A and Supplemental Table 3), leading to death of the animals at the age of 17–21 days (Figure 6B and Supplemental Table 3). Both histology and PHOX2B staining of the tumors indicated they corresponded to neuroblastoma (Figure 6C). Expression of both the mutant *FGFR1* transcript and FGFR1 protein in the tumor was validated by RNA-Seq and Western blot analysis, respectively (Figure 6D and Supplemental Figure 7, A and B).

Because we had observed more aggressive disease courses in patients with *FGFR1*-mutant high-risk neuroblastoma than in those with *ALK* mutations, we compared the phenotype of *FGFR1^N546K^;Th-MYCN* mice with that of *Th-ALK^F1174L^;Th-MYCN* mice, a well-established murine neuroblastoma model corresponding to a recurrent genotype in human neuroblastoma (38, 39). The combined expression of mutant *ALK* and *MYCN* also resulted in tumor formation with 100% penetrance, whereas heterozygous expression of the *MYCN* transgene alone led to macroscopic tumor development in 3% of the cases (*n* = 2 of 67 mice) only, as described previously (Supplemental Tables 3 and 4) (38, 39). In comparison with *FGFR1^N546K^;Th-MYCN* mice, neuroblastomas developed later in *Th-ALK^F1174L^;Th-MYCN* mice, which led to death at the age of 6–8 weeks, indicating *FGFR1^N546K^* might have a stronger oncogenic potential than *ALK^F1174L^* (Figure 6B, Supplemental Figure 7, C and D, and Supplemental Table 4). The aggressive tumor phenotype observed in *FGFR1^N546K^;Th-MYCN* mice thus resembled that of *FGFR1^N546^*-mutant neuroblastoma in high-risk patients.

### FGFR1^N546K^;MYCN- and ALK^F1174L^;MYCN-driven neuroblastomas differ in their molecular profiles.

To determine the molecular basis of the distinct phenotypes observed in *FGFR1^N546K^*-, *FGFR1^N546K^;MYCN*-, and *ALK^F1174L^;MYCN*-driven neuroblastomas, we generated RNA-Seq data from tumors of these 3 subtypes (Supplemental Table 5). Unsupervised analysis of expression data by t-distributed stochastic neighbor embedding revealed that these 3 genetically defined neuroblastoma types formed distinct clusters (Supplemental Figure 8A). We then determined differentially expressed genes between *FGFR1^N546K^*- and *FGFR1^N546K^;MYCN*-driven neuroblastomas, followed by gene set enrichment analysis (40–42), which uncovered enrichment of cell cycle– and proliferation-associated and adrenergic gene sets in the latter (Supplemental Figure 8, B and C, and Supplemental Table 6). By contrast, cell cycle and proliferation gene sets were depleted, and both adrenergic and mesenchymal gene sets were enriched in *FGFR1^N546K^;MYCN* in comparison with *ALK^F1174L^;MYCN*-driven neuroblastomas (Supplemental Figure 8, D and E) (43, 44).

**Figure 7:**
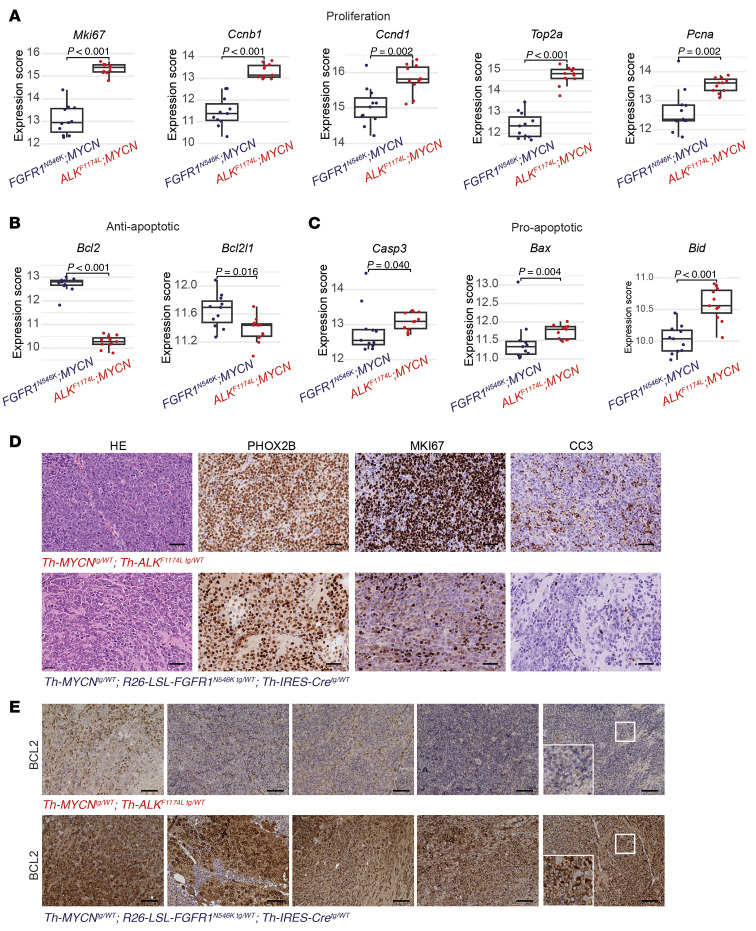
*FGFR1^N546K^;MYCN*-driven and *ALK^F1174L^;MYCN*-driven tumors differ in the expression of proliferation-associated and apoptotic markers. (**A**–**C**) Expression levels of genes associated with proliferation (*Mki67*, *Ccnb1*, *Ccnd1*, *Top2a*, and *Pcna* (**A**); anti-apoptotic genes (*Bcl2* and *Bcl2l1*) (**B**); and pro-apoptotic genes (*Casp3, Bax*, and *Bid*) (**C**) in tumors of *R26-FGFR1^N546K/wt^;Th-IRES-Cre^tg/wt^;Th-MYCN^tg/wt^* and *Th-ALK^F1174L/wt^;Th-MYCN^tg/wt^* mice, as determined by RNA-Seq. Box plots show the median, first, and third quartiles; the whiskers represent the minimum and maximum values within ±1.5 times the interquartile range. Comparisons between groups were performed using a 2-tailed Wilcoxon rank-sum test. (**D**) H&E and PHOX2B, Ki67, and cleaved caspase 3 (CC3) immunohistochemical staining of tumor sections obtained from a *Th-ALK^F1174L/wt^;Th-MYCN^tg/wt^* mouse (56 days old; top) and from an *R26-FGFR1^N546K/wt^;Th-IRES-Cre^tg/wt^;Th-MYCN^tg/wt^* mouse (20 days old; bottom); scale bar, 50 μm. (**E**) BCL2 immunohistochemical staining of tumor sections obtained from of *Th-ALK^F1174L/wt^;Th-MYCN^tg/wt^* (top) and *R26-FGFR1^N546K/wt^;Th-IRES-Cre^tg/wt^;Th-MYCN^tg/wt^* (bottom) mice; scale bar: 100 μm.

Because these data unexpectedly suggested that *ALK^F1174L^*, in combination with *MYCN*, triggers a higher proliferation rate of the tumor cells than *FGFR1^N546K^;MYCN*, we examined differentially expressed genes of these 2 tumor types in more detail (Supplemental Table 7). Indeed, we found that proliferation-associated genes, such as *Mki67*, *Ccnb1*, *Ccnd1*, *Top2a*, and *Pcna*, were expressed at higher levels in *ALK^F1174L^*-driven tumors (Figure 7A). We speculated, therefore, that the more aggressive growth of tumors in *FGFR1^N546K^;MYCN* mice was due not only to increased proliferation but also to a shifted balance between pro- and anti-apoptotic signals in the malignant cells, leading to impaired cell death in these tumors. In line with that notion, we observed that the anti-apoptotic genes *Bcl2* and *Bcl2l1* were significantly upregulated, whereas pro-apoptotic *Casp3*, *Bax, Bid*, and *Bcl2l11* were downregulated in *FGFR1^N546K^;MYCN*-driven tumors (Figure 7, B and C, Supplemental Figure 8F, and Supplemental Table 7).

We validated reduced protein levels of MKI67 and cleaved caspase 3 in *FGFR1^N546K^;MYCN*-driven neuroblastoma by immunohistochemistry (Figure 7D and Supplemental Figure 9A), as well as elevated levels of BCL2 by immunohistochemistry and Western blot analysis (Figure 7E, Supplemental Figures 7B and 9B). We also evaluated apoptotic DNA fragmentation by TUNEL staining and found only few positive cells in *FGFR1^N546K^;MYCN*-driven tumors, whereas TUNEL-positive cells were abundant in *ALK^F1174L^;MYCN*-driven neuroblastomas (Supplemental Figure 9, C and D). Together, these data suggest the aggressive growth of tumors bearing *FGFR1^N546K^* may be due to impaired mechanisms of cell death when compared with *ALK^F1174L^*-driven tumors, which is in line with the poor response to chemotherapy observed in patients.

### FGFR inhibition impairs tumor growth and prolongs survival in FGFR1^N546K^;MYCN transgenic mice.

We next asked whether targeting mutated FGFR1 with FGFR inhibitors might impair *FGFR1^N546K^*-driven tumor growth in vivo. To this end*,* we genotyped *R26-FGFR1-N546K^N546K/wt^;Th-MYCN^tg/wt^;Th-IRES-Cre^tg/wt^* mice at the age of 10–13 days and confirmed tumor development by MRI at the age of 14–15 days. We then immediately started treatment with futibatinib, which has been used at a broad dosing range (0.5–50 mg/kg body weight per day) in previous mouse experiments (32, 45–47).

**Figure 8:**
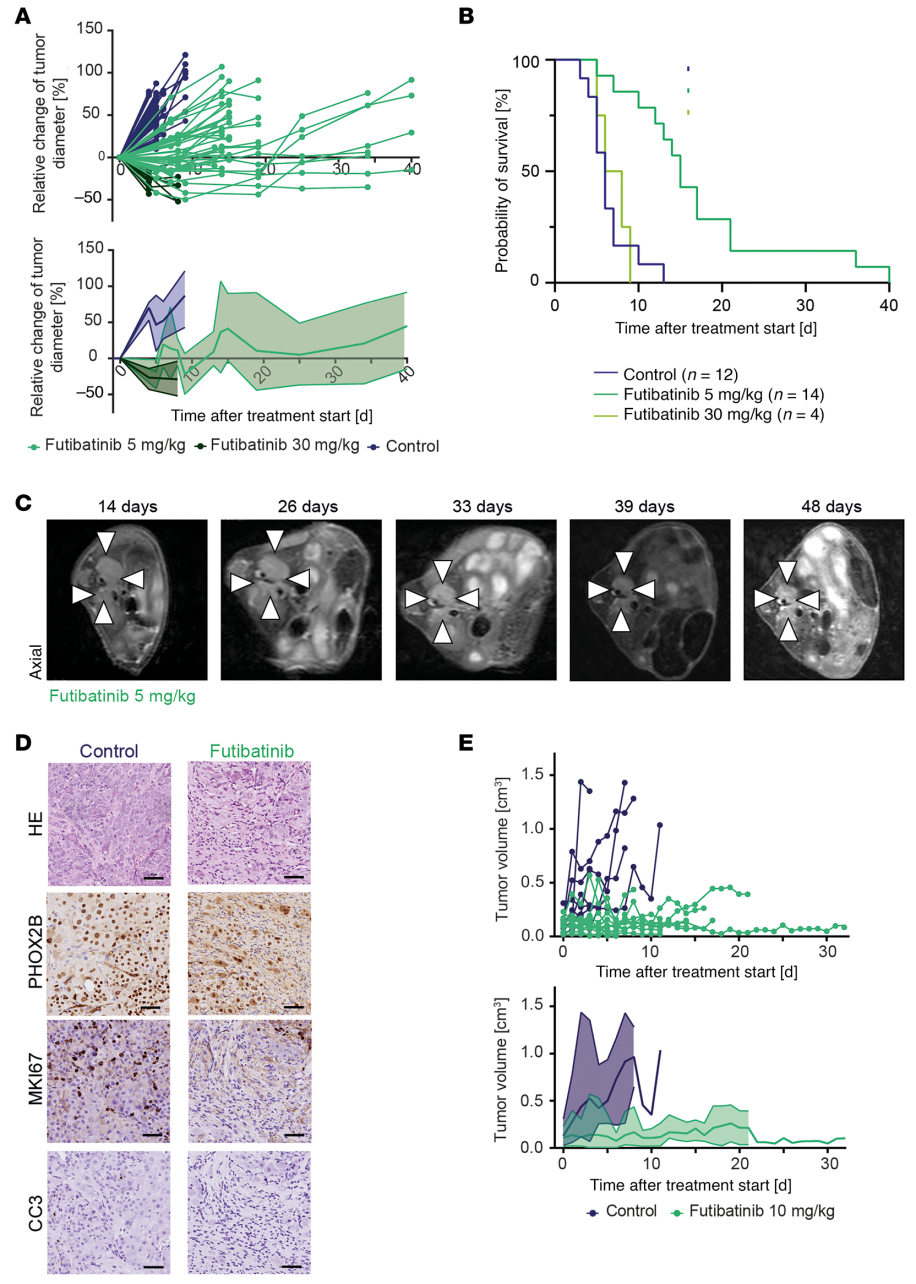
Futibatinib impairs tumor growth and prolongs survival in *FGFR1^N546K^;MYCN* transgenic mice. (**A**) Temporal changes of relative tumor diameters in *R26-FGFR1^N546K/wt^;Th-IRES-Cre^tg/wt^;Th-MYCN^tg/wt^* mice treated with 5 mg/kg futibatinib (light green), 30 mg/kg futibatinib (dark green), or control substance (0.5% CMC-Na; blue). Growth curves of individual tumors are shown on the left; mean and range of the relative tumor diameter changes are shown on the right. (**B**) OS of *R26-FGFR1^N546K/wt^;Th-IRES-Cre^tg/wt^;Th-MYCN^tg/wt^* mice treated with 5 mg/kg, or 30 mg/kg futibatinib, or control. Survival of mice treated with 5 mg/kg futibatinib was significantly longer than that of control mice (*P* < 0.001), whereas survival of mice treated with 30 mg/kg futibatinib was not significantly prolonged, due to toxicity (*P* = 0.47). (**C**) Axial T2-weighted MRI scans of an *R26-FGFR1^N546K/wt^;Th-IRES-Cre^tg/wt^;Th-MYCN^tg/wt^* mouse treated with 5 mg/kg futibatinib at the indicated ages. (**D**) H&E and PHOX2B, Ki67, and CC3 immunohistochemical staining of tumor sections obtained from *R26-FGFR1^N546K/wt^;Th-IRES-Cre^tg/wt^;Th-MYCN^tg/wt^* mice treated with control (top) or 5 mg/kg futibatinib (bottom); scale bar, 50 μm. (**E**) Absolute volumes of tumors obtained from *R26-FGFR1^N546K/wt^;Th-IRES-Cre^tg/wt^;Th-MYCN^tg/wt^* mice, subcutaneously reimplanted into NSG mice and treated with 10 mg/kg futibatinib (*n* = 11) or control (*n* = 9) after reaching a volume of 0.08–0.2 cm^3^. Growth curves of individual tumors are shown on the left; mean and range of tumor volumes are shown on the right.

At a daily dose of 30 mg/kg, we observed reduction in tumor size in all treated mice (*n* = 4) (Figure 8A and Supplemental Figure 10, A–C), whereas control mice had rapid tumor progression, as expected (Supplemental Figure 10, A, B, and D). Tumor response in futibatinib-treated mice, however, was not accompanied by prolonged survival (Figure 8B and Supplemental Table 8), which was probably due to impaired tolerability of the compound and/or the administration procedure; mice developed weight loss (>10% of the body weight) and reduced condition (measured by various parameters defined in the score sheet we used) over treatment. Therefore, we lowered the dose of futibatinib to 5 mg/kg/day, which resulted in partial tumor remission or decelerated tumor growth, and significantly prolonged survival of the treated cohort in comparison with the control cohort (Figure 8, A–C, and Supplemental Table 8). We again noted, however, that futibatinib- and control-treated mice concordantly showed weight loss and reduced condition, suggesting the oral gavage procedure itself may cause these symptoms, which may limit the feasibility of oral drug administration, particularly in mice of this young age (48).

Histological analysis of treated tumors revealed impaired proliferation as compared with controls, as indicated by reduction in MKI67-positive cells (Figure 8D and Supplemental Figure 10E). We also observed focal enrichment of TUNEL-positive cells, indicating apoptotic DNA fragmentation; however, we did not observe increased fractions of cleaved caspase 3–positive cells by immunohistochemistry (Figure 8D and Supplemental Figure 10, E–G).

To compensate for the limitations caused by gavage and/or futibatinib toxicity in mice of very young age and small size, we reimplanted tumor specimens obtained from R*26-LSL-FGFR1^N546K/wt^;Th-MYCN^tg/wt^;Th-IRES-Cre^tg/wt^* mice in immunocompromised NSG mice, thus enabling evaluation of the therapeutic efficacy of FGFR inhibition in older mice with higher body weight at the start of treatment. Here, we found that treatment with futibatinib at an increased dose of 10 mg/kg almost completely abrogated tumor growth and significantly prolonged survival of the mice (Figure 8E, Supplemental Figure 11, A and B, and Supplemental Table 9). We again observed, however, that mice had to be sacrificed due to weight loss without having reached the maximum tumor volume, both in the futibatinib- and the control-treated cohorts, supporting the notion that the procedure of gavage or the solvent may be associated with stress and reduced food intake.

### FGFR inhibition has antitumor activity in human FGFR1^N546K^-mutant neuroblastoma models.

We next examined the antitumor activity of pharmacological FGFR inhibition in human *FGFR1^N546K^*-mutant neuroblastoma models. Exposure of a human *FGFR1^N546K^*-mutant, *MYCN*-amplified cell line to futibatinib led to both dose-dependent reduction of cell viability and decreased phosphorylation of FGFR1 and its downstream targets (Supplemental Figure 11, C–E), similar to results in Ba/F3 cells (Figures 3 and 4). Likewise, we observed dose-dependent reduction of cell viability in a patient-derived, *MYCN-*amplified organoid model upon exposure to erdafitinib or futibatinib, whereas this model was largely resistant to cytotoxic agents used in neuroblastoma therapy (Supplemental Figure 11F).

We also evaluated the antitumor effect of FGFR inhibition in a non–*MYCN*-amplified, *FGFR1^N546K^*-mutated, chemotherapy-resistant, patient-derived xenograft (PDX) model that had been generated at the time of relapse from patient 8 (Figure 1; ITCC-P4_s15_NB0675; ref. 49). The patient had stage 2A disease at diagnosis but had a metastatic relapse with pleural metastases and did not respond to relapse therapy. Tumors were implanted subcutaneously in immunodeficient NOD/Shi-scid/IL-2Rγ^null^ (NOG) mice after confirmation of the *FGFR1^N546K^* mutation (Supplemental Figure 12, A and B). Mice were randomized into treatment and control groups, and treatment with futibatinib at 10 mg/kg/day or control substance was started when tumors had reached a volume of 0.08–0.2 cm³. In line with the results from the murine re-implantation experiment, we observed that treatment with futibatinib at this dose led to deceleration of tumor growth and prolonged survival in comparison with control mice (Supplemental Figure 12, C and D). Futibatinib treatment was tolerated well by immunodeficient NOG mice over 21 days. We did not observe body weight loss of more than 10% or symptoms potentially related to hyperphosphatemia, such as diarrhea, muscle cramps, or lethargy, in 2 test mice used to determine the tolerability of 10 mg/kg futibatinib (Supplemental Figure 12E). Phosphate blood levels after 2 weeks treatment were within the reference range (Supplemental Figure 12F).

**Figure 9:**
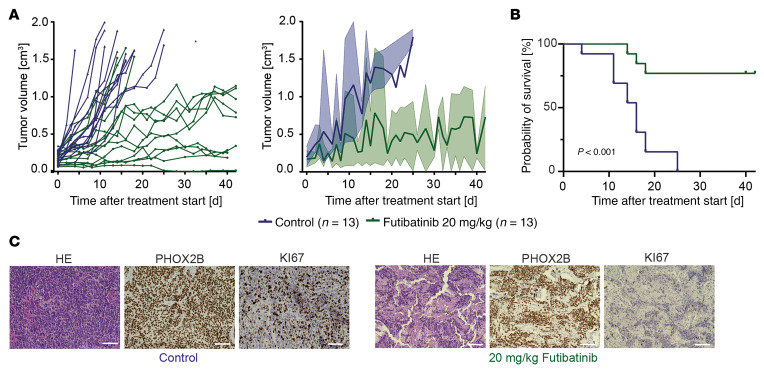
Futibatinib abrogates tumor growth in an *FGFR1^N546K^*-mutant, patient-derived xenograft model. (**A**) Absolute volumes of individual tumors (left) and mean and range of tumor volumes (right) of an *FGFR^N546K^*-mutant, patient-derived xenograft mouse model, treated with control (10% DMSO + 90% [20%] Captisol in 0.9% NaCl); *n* = 13) or 20 mg/kg futibatinib (futibatinib diluted in 10% DMSO + 90% [20%] Captisol in 0.9% NaCl); *n* = 13). (**B**) OS of mice bearing an *FGFR^N546K^*-mutant, patient-derived xenograft, treated with control or 20 mg/kg futibatinib. Survival curves were estimated according to Kaplan-Meier test and compared with a log-rank test. (**C**) H&E and PHOX2B and Ki67 immunohistochemical staining of tumor sections obtained from patient-derived xenograft tumors treated with a control substance (top) of 20 mg/kg futibatinib (bottom); scale bar, 100 μm.

Because our previous results suggested dose-dependent antitumor effects of futibatinib, we asked whether higher doses may enhance growth inhibitory effects in this model. Because treatment was well tolerated at 10 mg/kg futibatinib daily, we treated a second cohort of mice at 20 mg/kg/day (Figure 9A). We found that the increased dose improved the growth-inhibitory effect of futibatinib and survival of mice over the treatment period of 40–42 days (Figure 9B and Supplemental Figure 12G). Treatment with 20 mg/kg futibatinib led to body weight loss in some animals (Supplemental Figure 12H). After a 2-day drug holiday, however, treatment was restarted at the same dose level and was then well tolerated.

We found that treatment with 20 mg/kg futibatinib substantially reduced the number of MKI67-positive cells in the tumor when compared with control and treatment with 10 mg/kg futibatinib (Figure 9C and Supplemental Figure 13A). However, we again did not observe more cleaved caspase 3–positive cells or increased fractions of TUNEL-positive cells (Supplemental Figure 13, B and C). Together, the results obtained from murine and human models treated with futibatinib demonstrate that pharmacological inhibition of FGFR effectively impairs growth of *FGFR1^N546K^*-mutated neuroblastoma and that the antitumor effect is dose dependent.

### Addition of futibatinib to low-intensity chemotherapy has clinical benefit in a patient with FGFR1^N546K^-mutated neuroblastoma.

**Figure 10:**
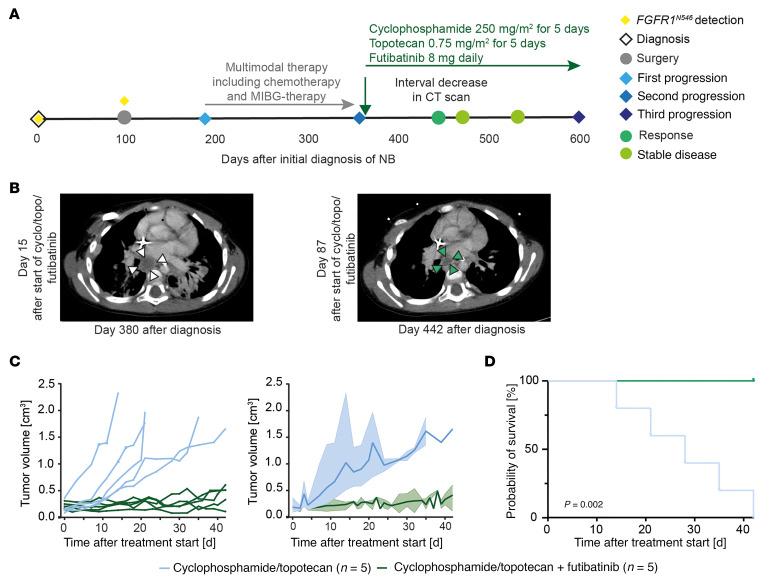
Futibatinib shows clinical benefit in a patient with *FGFR1*-mutated neuroblastoma (NB). (**A**) Schematic timeline of the clinical course of a patient with *FGFR1^N546K^*-mutated neuroblastoma that progressed under multiple lines of treatment and therefore was treated with a combination of futibatinib, cyclophosphamide, and topotecan. (**B**) Computed tomography (CT) scans of the tumor region in patient shown in (**D**) on day 15 after start of futibatinib/cyclophosphamide/topotecan (day 380 after diagnosis) and on day 87 (day 442 after diagnosis), demonstrating a partial regression of the tumor. (**C**) Absolute volumes of individual tumors (left) and mean and range of tumor volumes (right) of the patient-derived xenograft mouse model, treated with cyclophosphamide/topotecan alone (blue) or in combination with 20 mg/kg futibatinib (green). (**D**) OS of mice bearing the patient-derived xenograft, treated with cyclophosphamide/topotecan alone (blue) or in combination with 20 mg/kg futibatinib (green). Survival curves were estimated according to Kaplan-Meier test and compared with a log-rank test. MIBG, metaiodobenzylguanidine.

Finally, we evaluated the tolerability and efficacy of futibatinib in combination with chemotherapy in a patient with *FGFR1^N546K^*-mutant neuroblastoma with progressive disease despite multiple prior lines of therapy (Supplemental Table 10). The patient was initially diagnosed at the age of 2 years and 11 months with *MYCN* nonamplified, locoregional L2 neuroblastoma harboring an *FGFR1^N546K^* mutation. The patient received 2 cycles of induction chemotherapy (cyclophosphamide/topotecan and ifosfamide/carboplatin/etoposide) with no response to treatment (stable disease by INRC). Surgery was performed after these 2 cycles, and the presence of the mutation was confirmed in the surgical specimen. The patient then received a third cycle of chemotherapy (cyclophosphamide/doxorubicin/vincristine [CAV] per trial ANBL1531 [Clinical Trials.gov NCT03126916]), followed by clinical observation. Mediastinal nodal and intraabdominal disease progression occurred 184 days after initial diagnosis. The patient then received multiple rescue therapies, including chemotherapy (1 cycle CAV; 2 cycles of irinotecan/temozolomide/dinutuximab, per trial ANBL1221; and 1 cycle of cisplatin/etoposide, per trial ANBL1531 [Clinical Trials.gov NCT017767194]) and ^131^I-metaiodobenzylguanidine therapy in combination with vorinostat. However, tumor progression occurred again under treatment, consistent with refractory disease. Considering the presence of mutated *FGFR1*, the patient was then treated with cyclophosphamide (250 mg/m^2^) and topotecan (0.75 mg/m^2^) daily for 5 days in a 28-day cycle in combination with futibatinib (8 mg) daily in an outpatient setting (Figure 10A). After 3 months of this combination therapy, a decrease of tumor diameter was observed on computed tomography scans (Figure 10, A and B), followed by stable disease for 5 months of therapy (*n* = 9 cycles). After this period, tumor progression occurred again, and the patient eventually succumbed to disease 3 months later (OS = 699 days).

To experimentally test the added antitumor activity of futibatinib in combination with cyclophosphamide/topotecan over the chemotherapy backbone alone, we administered the combination therapy to mice bearing the *FGFR1^N546K^*-mutant PDX (Figure 9), which was derived from patient 8 (Figure 1 and Supplemental Figure 1) with a similar history of chemotherapy-resistant neuroblastoma. Whereas cytotoxic treatment with cyclophosphamide/topotecan alone had moderate antitumor efficacy in this model, addition of futibatinib almost completely abrogated tumor growth and led to survival of all mice over the treatment period (Figure 10, C and D, and Supplemental Figure 13D), thus supporting the potential relevance of FGFR1 inhibition in a treatment concept for refractory *FGFR1^N546K^*-mutant neuroblastoma.

In the patient, futibatinib treatment in combination with cyclophosphamide and topotecan was tolerated well in general. After 3 weeks of therapy, phosphate levels increased to 7.2 mg/dL (hyperphosphatemia grade 3; ref. 50), resulting in the need for a drug holiday until blood phosphate levels had decreased to normal. Phosphate intake was then restricted to 600–800 mg/day, accompanied by treatment with phosphate-binding medication (sevelamer 400 mg 3 times a day), which maintained normal phosphate levels with continued futibatinib treatment (Supplemental Figure 13E). Although additional experience is required, this case and results from the PDX model suggest addition of an FGFR inhibitor to a chemotherapy backbone may be tolerable and delay disease progression in patients with *FGFR1^N546K^*-mutated neuroblastoma.

## Discussion

Despite advancements over recent years (4, 8, 10, 15, 16, 37, 51), the genetic determinants of poor clinical outcome in neuroblastoma have remained incompletely understood, which has impaired implementation of molecularly guided therapies. Here, we demonstrate that mutations of *FGFR1* at codon 546, resulting in an amino acid exchange from asparagine to lysine or aspartic acid, are associated with chemoresistance and fatal disease progression in patients with high-risk neuroblastoma. We show that ectopic expression of *FGFR1^N546K^* results in cellular transformation in vitro and neuroblastoma development in genetically engineered mice. Expression of transgenic *FGFR1^N546K^* in combination with *MYCN* elicited highly aggressive murine tumors with dysregulated homeostasis of apoptotic cell death, corresponding to the chemotherapy-resistant and fatal phenotypes observed in patients. Importantly, we also demonstrate that pharmacological inhibition of FGFR1^N546K^ by clinically available FGFR-directed compounds results in growth inhibition, both in vitro and in vivo. Clinical benefit of FGFR inhibition in combination with low-intensity chemotherapy was also observed in a patient with relapsed neuroblastoma that was resistant to multimodal treatment. Together, our data demonstrate that *FGFR1^N546K^* is a prognostic variable associated with treatment resistance and devastating outcome, and a strong oncogenic driver in neuroblastoma pathogenesis that potentially provides a target for therapeutic interventions.

Mutations at codon 546 of *FGFR1* have been occasionally reported in neuroblastoma (10, 15, 16, 20) and in other tumor entities, such as pediatric low-grade glioma (26, 52–54); however, their pathogenetic, prognostic, and potential therapeutic relevance in neuroblastoma have remained unclear. In general, *FGFR1^N546^* mutations are rare in neuroblastoma, with a prevalence of approximately 1% at diagnosis in this and a previous study (15) and 2% at relapse. In line with our results, a trend toward inferior outcome for patients with *FGFR1^N546K^*-mutant tumors was observed in a previous study when compared with patients with *FGFR1^WT^* tumors (15). Other studies reported that ectopic expression of *FGFR1^N546K^* in neuroblastoma and other cells led to transformation and downstream pathway activation (both in line with our observations in Ba/F3 cells), and increased colony formation (20, 26). In contrast to our findings, however, ectopic expression of *FGFR1^N546K^* in neuroblastoma cell lines conferred resistance to pharmacological FGFR inhibition (20). The discrepant results may be due to a lack of *FGFR1^N546K^* addiction in established neuroblastoma cell lines bearing WT endogenous *FGFR1*, as opposed to the *FGFR1^N546K^*-expressing cellular and murine models used in our study.

p.N546K is the predominant amino acid substitution in *FGFR1*-mutant neuroblastoma; however, the variant p.N546D also recurs in this malignancy. Although experimental data on the transforming capacity of this variant are not available, to our knowledge, one might expect molecular and phenotypic effects that are similar to *FGFR1^N546K^*, because this variant has been predicted to be activating, and because patients had similar clinical courses (55–61). In support of this notion, a phase I first-in-human study of futibatinib reported partial response in a patient with glioblastoma harboring the *FGFR1^N546D^* variant (55).

We found that rapid disease progression occurred after detection of *FGFR^N546K^* in most patients with neuroblastoma, suggesting that these tumors are largely resistant to common chemotherapies. Consistent with that observation, we noted few antitumor effects of cytotoxic agents in human *FGFR1*-mutant neuroblastoma organoid and PDX models. In addition, elevated BCL2 levels and fewer apoptotic cells occurred in *FGFR1^N546K^;MYCN* as compared with *ALK^F1174L^;MYCN*-driven murine tumors. It has been reported that FGFR pathway activation by FGF2 can induce BCL2 in other cancer entities (62, 63). Given that BCL2 is an actionable target in human malignancies, further studies are warranted to elucidate the impact of mutant *FGFR1* on BCL2 expression and apoptosis in neuroblastoma.

We observed that survival of mice with *FGFR1-*mutated neuroblastoma was substantially shorter than that of mice with *ALK-*mutated neuroblastoma, indicating a stronger oncogenic potential of mutant FGFR1, which is in line with the aggressive phenotypes observed in patients with *FGFR1*-mutant tumors. It must be considered, though, that *FGFR1^N546K^* expression under control of the strong *CAG* promotor after Cre-mediated recombination might differ from *Th-ALK^F1174L^* expression, both in terms of temporal and spatial control. These factors may influence not only the timing of transgene activation but also overall expression dynamics, and more studies are needed, therefore, to compare the oncogenic potential of mutant *FGFR1* and *ALK*.

Treatment of high-risk neuroblastoma remains challenging, with disease in some patients being nonresponsive to current therapeutic strategies, especially at relapse (4). Identification of actionable targets, therefore, is important to develop more efficacious therapies and to improve outcome for affected patients. ALK is the most frequently mutated receptor tyrosine kinase in neuroblastoma, and several clinical trials have demonstrated that pharmacological inhibition of mutant ALK may benefit children with neuroblastoma (12, 13, 64). It is thus tempting to speculate that mutant FGFR1 may also serve as a therapeutic target in neuroblastoma, a notion that is supported by both the preclinical and clinical data presented in this study. We observed antitumor activity of FGFR inhibition in various neuroblastoma models, independent of potentially confounding factors, such as *MYCN* amplification, thus reinforcing the idea of pharmacological FGFR inhibition as a therapeutic concept in *FGFR1^N546^*-mutant neuroblastoma.

Several selective FGFR inhibitors have entered clinical trials in recent years, and erdafitinib, pemigatinib, infigratinib, and futibatinib were recently approved by the FDA for treatment of adult patients. First results from clinical trials on FGFR inhibitors in children with cancer have reported promising preliminary data regarding their antitumor effect (65, 66). The impact of single-agent erdafitinib on recurrent and refractory pediatric tumors with *FGFR* alterations is being investigated in the Molecular Analysis for Therapy Choice (MATCH) phase 2 trial (ClinicalTrials.gov identifier NCT03210714). However, only 1 patient with neuroblastoma has been recruited to date, and partial responses have been reported only in patients with gliomas or glioneuronal tumors, leaving the benefit to patients with neuroblastoma unclear (67). The RAGNAR trial (ClinicalTrials.gov identifier NCT04083976) is another ongoing, phase 2, histology-agnostic trial investigating the efficacy and safety of erdafitinib in pretreated patients older than 6 years (68). In contrast to erdafitinib and other FGFR inhibitors, futibatinib binds irreversibly to FGFR family members, and on-target resistance mutations may develop less frequently (32, 69). Futibatinib is being assessed in the Targeted Agent and Profiling Utilization Registry (TAPUR) phase 2 clinical trial for advanced solid tumors with FGFR alterations in children aged 12 years or older (ClinicalTrials.gov identifier NCT02693535) and the European Proof-of-Concept Therapeutic Stratification Trial of Molecular Anomalies in Relapsed or Refractory Tumors (ESMART) trial (ClinicalTrials.gov identifier NCT02813135). However, inclusion of patients with neuroblastoma in such trials is limited by age eligibility criteria and the low *FGFR1* mutation frequency, which hampers evaluation of neuroblastoma-specific effects within clinical studies.

In patients with cancer, monotherapy with tyrosine kinase inhibitors often results in drug resistance, which may be overcome by combining tyrosine kinase inhibitors with other anticancer drugs. In our study, we found preliminary preclinical and clinical evidence that combination of the FGFR inhibitor futibatinib with low-intensity chemotherapy may be tolerable and enhance the antitumor efficacy of cytotoxic treatment in *FGFR1^N546K^*-mutant neuroblastoma. The additional benefit of FGFR inhibition over chemotherapy alone in the patient reported in this study is supported by the patient’s prior chemotherapy resistance, including nonresponse to the same chemotherapy backbone (topotecan/cyclophosphamide) in first-line treatment and disease progression upon a similar cytotoxic regimen (irinotecan/temozolomide) plus the anti-GD2 antibody dinutuximab at relapse. In line with this notion, we also found significantly improved efficacy of cyclophosphamide/topotecan after addition of futibatinib in an *FGFR1*-mutant PDX mouse model. However, because our findings on combination treatment are based on results from a single PDX mouse model and 1 patient only, further studies are required to assess the efficacy and tolerability of FGFR inhibitors in combination with chemotherapy in *FGFR1*-mutant neuroblastoma. Alternatively, it is tempting to speculate that combination of FGFR inhibition with drugs that target downstream signaling of mutant FGFR1, such as BCL2 or AKT, may improve therapeutic efficacy in *FGFR1*-mutant neuroblastoma.

Taken together, our data suggest consideration of pharmacological FGFR inhibition as a therapeutic strategy in the treatment of patients with *FGFR1^N546K^*-mutant, high-risk neuroblastoma, particularly in light of the poor outcome for these patients. Our study, however, also illustrates a major dilemma of pediatric oncology: although our results support the notion that FGFR-directed therapies may benefit patients with *FGFR^N546K^*-mutated neuroblastoma, their rarity may prevent inclusion of sufficient numbers of such patients in clinical trials and, thus, approval of FGFR inhibitors for this cancer type. To solve this dilemma, novel drug approval strategies may warrant consideration.

## Methods

A detailed description of the Methods is given in the Supplemental Methods.

### Sex as a biological variable.

Sex was not considered as a biological variable.

### Patients.

We included all patients with tumors with confirmed *FGFR1^N546^* mutations. Informed consent was obtained from patients’ legal guardians. Patients had been registered and treated according to different trials. Informed consent for the off-label use of futibatinib was obtained from the patients’ legal guardians. Further information on the patients can be found in the Supplemental Methods.

### Site-directed mutagenesis.

Site-directed mutagenesis was performed according to the manufacturer’s instructions, using the Q5 Site-Directed Mutagenesis Kit (New England Biolabs) (see Supplemental Methods).

### Bacterial transformation.

Transformation of Electrocomp *E*. *coli* (Invitrogen) was performed via standard protocols by electroporation (Micropulser, BIO-RAD). Plasmid isolation was performed according to the QIAGEN plasmid kits.

### Stable virus transduction.

Virus transduction was performed using standard methods, as described previously (70) (Supplemental Methods).

### Cell lines.

Cell lines were cultured according to standard procedures (Supplemental Methods). All cells were incubated at 37°C and 5% CO_2_ and were negatively tested on *Mycoplasma*.

### Cytokine independence assay.

Ba/F3 cells were seeded at 1 × 10^5^ cells/mL and cultured with 10 and 0 ng/mL IL-3. Cell numbers and viability were measured after 144 hours using a Cedex XS Cell Analyzer.

### Western blot.

Immunoblotting was performed using standard procedures. A detailed description can be found in the Supplemental Methods.

### Cell viability assay.

Ba/F3 cells were plated and treated in 96-well plates (10,000 cells/well) using a Biomek 400 automated liquid handler. Viability was measured by CellTiter-Glo assay (Promega) after 72 hours. Luminescence was measured by SpectraMax i3x after 15 minutes to quantify viable cells.

### Genetically engineered mouse model.

The *R26-LSL-FGFR1^N546K^* genetically engineered mouse line was generated by Taconic Biosciences and crossbred with the established *Th-MYCN* and *Th-IRES-Cre* mouse lines (33, 38). Genotyping PCR was performed according to standard protocols with primers indicated in Supplemental Table 11. For OS analyses, animals that succumbed to disease or had to be sacrificed due to termination criteria were recorded as events. Mice were euthanized when they reached termination criteria defined in the score sheet. A detailed clinical and line-specific score sheet assessing body weight, general condition, behavior, and clinical signs and tumor-related parameters was used to evaluate termination criteria. 

### MRI.

MRI was performed as described previously (70).

### Histopathology and immunohistochemistry.

Tumors and organs were harvested and fixed in 4% PBS-buffered FFPE. FFPE tissue (3 μm sections) was deparaffinized and treated according to standard protocols of the routine diagnostics pipeline (Institute for Pathology, University Hospital Cologne, Germany). A detailed description can be found in Supplemental Methods.

### RNA-Seq.

RNA isolation from fresh-frozen tissue was performed with TRIzol (Ambion). RNA concentration was measured by Qubit Assay (Invitrogen), and quality was assessed by 2100 Bioanalyzer (Agilent). mRNA-Seq (paired end, 2 × 100 bp, 50 M reads) was conducted by the Cologne Center for Genomics according to standard procedures. Quantification of RNA-Seq data was carried out using Kallisto (version 0.44.0). Differential gene expression analysis was performed as described in other reports (41, 42).

### TUNEL assay.

TUNEL staining was performed according to the manufacturer’s instructions, using the In Situ Cell Death Detection Kit, TMR red (Roche) (see Supplemental Methods).

### In vivo experiments.

Mice were genotyped at the age of 10 days and weekly MRI was performed starting at the age of 13–15 days to evaluate tumor diameters. All mice with tumors were included in the treatment cohorts. Mice were randomized into treatment groups. For intervention trials, animals were treated with 5 and 30 mg/kg futibatinib dissolved in 0.5% sodium carboxymethyl cellulose (CMC-Na) with 10% DMSO or in the solvent without futibatinib as control. Futibatinib was administered by gavage daily (maximum volume per dose, 10 mL/kg). Blinding of investigators was not performed. Reimplantation of murine tumors was performed in female NOD-SCID mice (Charles River Laboratories) (Supplemental Methods).

### Generation of patient-derived cell lines.

STA-NB-1.2 cells are low-passage cultures derived from a patient with INSS stage 3, *MYCN*-amplified, high-risk neuroblastoma after chemotherapy at St. Anna Children’s Cancer Research Institute (Vienna, Austria) (Supplemental Methods).

### Organoid model.

Organoid generation and culturing, as well as drug screens, were performed as previously described (71).

### Generation of subcutaneous PDX model.

The establishment and characterization of PDX mouse models was performed as previously described (72, 73). The mice were administered daily futibatinib 10 mg/kg via oral application. Placebo control mice were treated with a corresponding vehicle alone (administered orally with 10% DMSO/90% Captisol [20%] in sodium chloride). Chemotherapy treatment was performed as follows: topotecan was administered on days 1–5 of a 21-day cycle at a dose of 0.05 mg/kg, cyclophosphamide was administered on day 1 of the cycle at a dose of 20 mg/kg (74). Futibatinib was then also administered daily at a dose of 20 mg/kg. To avoid potential toxicities of chemotherapy with futibatinib, we chose a low-intensity regimen of cyclophosphamide and topotecan.

### Figure generation.

Figures were generated using Adobe Illustrator (RRID:SCR_010279; version 27.6), BioRender (RRID:SCR_018361; 2023), GraphPad Prism (RRID:SCR_002798; 9.5.1), Integrative Genomics Viewer (version 2.17.3), and R (version 4.2.2) with packages ggplot2 (version 3.5.0), maftools (version 2.18.0), and Rtsne (version 0.17).

### Statistics.

Statistical analyses were carried out with R (version 4.2.2) and Prism (9.5.1). Disease-specific survival was calculated as the time from diagnosis to death from disease or the last follow-up. Survival curves were estimated according to Kaplan-Meier tests and compared with the log-rank test. Box plots mark the median, first, and third quartiles; their whiskers represent the minimum and maximum values within ±1.5 times the interquartile range. Comparisons between groups in box plots were performed using a 2-tailed Wilcoxon rank-sum test unless noted otherwise. *P* values ≤0.05 or less were considered statistically significant.

### Study approval.

All animal experiments were conducted in accordance with Federation of European Laboratory Animal Science Associations recommendations. The mice were housed in a specific-pathogen-free facility, and animal breeding and experiments were approved by the local animal care committee and the relevant authorities (Landesamt für Natur, Umwelt und Verbraucherschutz Nordrhein-Westfalen, AZ: approvals 81-02.04.2019.A009; 81-02.04.2024.A089; and 84-02.04.2020.A146). For human data and samples, written informed consent was obtained from the legal guardians.

### Data availability.

Newly generated sequencing data are available from the Gene Expression Omnibus database (https://www.ncbi.nlm.nih.gov/geo/) under accession number GSE313168. All Supporting data values generated and analyzed in this study are provided in the Supporting Data Values file included with this article.

## Author contributions

LW, JB, MF conceived and designed the study. LW, JB, JP, FI, SH, CB; CR, AMH, YK, NH, NI, MAD, BD, JMW, AMS, R Bagatelle, AGH, AE, TS, BH, MB, STM, R Büttner, MP, and MF collected and assembled the data. FI, KS, OW, KB, AGWR, MWG, KL, JM, MB, STM, DH, SLG, LC, GB, MC, LFS, RB, JMP, GS, FW, and NNS recruited and/or treated the patients and provided clinical data. JHS, FW, FM, AGH, HG and AE provided data and experimental models. LW, JB, and MF wrote and edited the manuscript. HCR and RKT revised the manuscript critically for important intellectual content. All authors provided comments on and approved the final manuscript.

## Funding support

- Deutsche Forschungsgemeinschaft: grants SFB1399 (grant ID 413326622; to LW, AMS, R Büttner, HG, MP, HCR, RKT, and MF), SFB1588 (grant ID 493872418; to AE, JHS, AGH, FW, and MF), FI 1926/2–1 (to MF), BA 6984/1–1 (to CB).
- Leverkusen hilft krebskranken Kindern e.V.
- Förderverein für krebskranke Kinder e.V. Köln: endowed chair (to MF).
- CANTAR Network (an initiative of the Ministry of Science of the State of North: Rhine–Westphalia, Germany): grant NW21-062B (to CR, RT, MP, and MF).
- German Federal Ministry of Education and Research: e:Med initiative, grants 01ZX1303, 01ZX1603, 01ZX1307, and 01ZX1607 (to MF) and 01ZX1901A and 01ZX2201A (to RKT, MP, and HCR).
- Deutsche Krebshilfe: Mildred Scheel Nachwuchszentrum grant 70113307 (to JB and SH).
- Else Kröner-Fresenius-Stiftung: grants 2016-Kolleg-19 (to CR) and 2020_EKFK.19 (to AMH).
- Cancer Research UK: Clinician Scientist Fellowship (to SLG).
- ZonMw: project no. 848101004 (iTHER study).
- Dutch Foundation Children Cancer-free: KiKa Core funding (iTHER study).
- Collaboration project in the Program “Preclinical Drug Development — Targeting Transcriptional Addiction in Cancer” (TACTIC) (to RKT).

## Supplementary Material

Supplemental data

Unedited blot and gel images

Supplemental tables 1-11

Supporting data values

## Version 1 02/12/2026

In-Press Preview

## Version 2 04/01/2026

Electronic publication
