# Supplementary material for: Mutated FGFR1 is an oncogenic driver and therapeutic target in high-risk neuroblastoma
Source: J Clin Invest. 2026 Feb 12;136(7):e189152. doi: 10.1172/JCI189152 (PMC13038208; doi:10.1172/JCI189152)
Supplement: Supplemental data [file jci-136-189152-s256.pdf]

Supplemental Material

Methods

Patients

We included all patients with tumors with confirmed *FGFR1*^{N546} mutations. Patients had been registered and treated according to the trials NB2004-HR (n=4) and NB Registry 2016 (n=2) of the Gesellschaft für Pädiatrische Onkologie (GPOH, patients from Germany). Tumors from stage 4/M patients were slightly overrepresented in the German cohort of neuroblastomas obtained at diagnosis (148/239 patients, 61.9%). One patient was treated according to the Low and Intermediate Risk Neuroblastoma European Study of the European Society for Pediatric Oncology Neuroblastoma (SIOPEN/LINES, NCT01728155) (n=1, patient from Austria). Part of the data (n=3) evaluated in this study was produced and kindly provided by the INFORM program (70-73). Collection and use of specimens were approved by the Institutional Review Board of the Medical Faculty of the University of Cologne, the Medical University of Vienna (EK#1853/2016, EK#1216/2018) and the UK national SMPaeds study (UK National ethics study reference 246557/264925). The patient from Norway was treated according to the LINES protocol intermediate risk as best available treatment. Analysis for FGFR was done as part of the diagnostic workup. Informed consent from legal guardians were given March 14th 2024 for giving limited anonymous patient data to this study on FGFR-mutated patients. The French patient was treated according to the HR-NBL1/SIOPEN trial. Informed consent was obtained from the legal guardians. Biological analyses were authorized within the research program IC2007-09 “Diagnostic and prognostic classification of neuroblastoma”, authorized by the ethics committee CPP II de France 1, Reference 0811827. The patient from Italy had been registered and treated according to the HR-NBL1/SIOPEN trial. Informed consent was obtained from the legal guardians. Use of specimens was approved by the Ethic Committee of the University of Naples Federico II. A freshly resected neuroblastoma sample was collected as part of the iTHER study of the Princess Máxima Center for Pediatric Oncology, Utrecht,

Netherlands (Netherlands Trial Register, Trial NL5728; NL56826.078.16). Ethics approval was granted. The patient's legal representative signed informed consent. Assent for paired tumor/germline testing in the Children's Hospital of Philadelphia (CHOP) clinical genomics laboratory was obtained for patients treated at CHOP. The contribution of deidentified patient data for this study met the CHOP Committee for the Protection of Human Subjects (IRB) criteria for exemption. Informed consent for the off-label use of futibatinib was obtained from the patient's legal guardians. Furthermore, separate informed consent was obtained for the patient's participation in the Memorial Sloan Kettering Cancer Center's prospective tumor/germline sequencing protocol (ClinicalTrials.gov Identifier: NCT01775072). Futibatinib was obtained through the patient's commercial insurance plan under an accepted off-label use.

Site-Directed Mutagenesis

pBabe-puro was a gift from Hartmut Land & Jay Morgenstern & Bob Weinberg (Addgene plasmid # 1764 ; http://n2t.net /addgene:1764 ; RRID:Addgene_1764). The retroviral *pBABE*-puro backbone vector (74) with cloned coding sequence of *FGFR1 beta* (Transcript ID ENST00000397091.9) was used. *FGFR1*^{N546K} point mutation and the kinase-dead mutant *FGFR1*^{D623A} in combination with and without *FGFR1*^{N546K} were generated by using the Q5 Site-Directed Mutagenesis Kit (New England Biolabs). Following primers were designed by NEBaseChanger: SDM_FGFR1_N546K_For (5´ ATAT-CATCAAGCTGCTGGGGG 3´) and SDM_FGFR1_N546K_Rev (5´ TCTTATGCTTCCCGAT-CATC 3´), SDM_FGFR1_D623A_For (5´ ATACACCGAGCCCTGGCAGCC 3´) and SDM_ FGFR1_ D623A_Rev (5´ GCACTTCTTGGAGGCCAG 3´).

Stable Virus Transduction

The *pBABE-puro* (RRID:Addgene_1764) retroviral vectors containing wild-type or mutated *FGFR1* (*FGFR1*^{D623A}, *FGFR1*$^{D623A;N546K}$, *FGFR1*^{N546K}) as well as an empty vector were used for retroviral transduction according to standard procedures. HEK293T cells were grown at 70% confluency. The *pBABE* vector (RRID:Addgene_21836) with gene of interest was mixed with the retroviral packaging vector pCl-Eco (RRID:Addgene_12371), OptiMEM and TransIT-LT1

(Mirus) and added to the cells followed by incubation for 20 minutes. Viral supernatant was collected after 48 hours and sterile filtered followed by the stable transduction of Ba/F3 cells. Ba/F3 cells were seeded and viral supernatant (2/3 of final volume) and fresh media (1/3 of final volume) supplemented with 0.7 µg/ml polybrene (Santa Cruz) were added. The media were replaced after 48 hours by fresh media containing 3 µg/ml puromycin (Sigma-Aldrich) for negative selection of non-transduced cells.

Western Blot

Cells were lysed in RIPA Lysis and Extraction Buffer (Thermo Fisher Scientific) supplemented with 40 µl/ml Protease and Phosphatase Inhibitor Cocktail (Life technologies) using the manufacturer's instructions. Protein concentrations were measured by Bradford Assay. Immunoblotting was performed using standard procedures. Membranes were incubated at 4°C with primary antibodies against: FGFR1 (Cell Signaling, #9740; 1:1000), phospho-FGFR1 Tyr653/654 (Sigma-Aldrich, #06-1433; 1:1000), FRS2 (Thermo Fisher Scientific, PA5-27875; 1:1000), phospho-FRS2α Tyr436 (Cell Signaling, #3861; 1:1000), STAT3 (Cell Signaling, #4909; 1:2000), phospho-STAT3 Tyr705 (Cell Signaling, #9145; 1:1000), AKT (Cell Signaling, #4685; 1:1000), phospho-AKT Ser473 (Cell Signaling, #9271; 1:1000), p44/42 MAPK (ERK1/2) (Cell Signaling, #4695; 1:1000), phospho-p44/42 MAPK (ERK1/2) Thr202/Tyr204 (Cell Signaling, #4370; 1:2000), β-Actin (Abcam, ab49900; 1:10000) and Bcl-2 (Cell Signaling, #3498; 1:1000). The next day, after washing membranes were incubated with following secondary antibodies for 1 hour at 4°C: Polyclonal Goat anti-Mouse Immunoglobulins (CiteAb, P0447; 1:1000) or anti-Rabbit IgG, HRP-linked Antibody (Cell Signaling, #7074; 1:1000). Detection was performed using Detection Reagent (Invitrogen) and visualized using a chemiluminescense detection system.

For treatment studies with FGFR inhibitors, cells were seeded at 4.4 x 10^6 cells/ml in serum starved media. After 24 hours, cells were treated with 10, 50 and 100 nM erdafitinib (JNJ-42756493) (Selleckchem, S8401) or futibatinib (TAS-120) (Selleckchem, S8848) and DMSO for 1 hour. Stimulation was conducted with 1 ng/ml recombinant FGF2 (Gibco) and 1 ng/ml

Heparin sodium (Braun) 20 minutes prior preparing lysates (75). For lysates of murine tumor tissue, 20 µm sections of fresh-frozen tumors were cut and lysed in RIPA Lysis and Extraction Buffer (Thermo SCIENTIFIC; 100 µl buffer for 30 mg tumor tissue) supplemented with 40 µl/ml Protease and Phosphatase Inhibitor Cocktail (Life technologies).

MR Imaging

Magnetic Resonance imaging (MRI) was performed on a clinical 3.0T MRI system (Achieva Quasar Dual, Philips Healthcare, Best, Netherlands) using a small rodent solenoid coil with an inner diameter of 40 mm and an implemented heating system to keep body temperature constant during MRI examination (Philips Research Europe, Hamburg, Germany). Animals were anesthetized by a 2.0 - 2.5% isoflurane inhalation. High resolution axial T2-weighted MR images were acquired using a multishot turbo-spin echo (TSE) sequence (repetition time (TR) = 5729 ms (cor) and 7639 ms (ax), echo time (TE) = 60 ms, flip angle (FA) = 90°, acquired resolution: 0.19 x 0.26 mm^2 in-plane, 1 mm slice thickness, turbo factor = 10). Images were exported in DICOM format and analyzed using the Horos software (Version v3.3.6). The maximum axial tumor diameter was used to evaluate the size of each tumor individually.

Histopathology

Tumors and organs were harvested and fixed in 4% PBS-buffered formalin for paraffin embedding (FFPE). Three µm sections from FFPE tissue, were deparaffinized and treated according to standard protocols of the routine diagnostics pipeline (Institute for Pathology, University Hospital Cologne, Germany). Briefly, tissue sections were deparaffinized and stained with Heamtoxylin and Eosin. Immunohistochemistry was performed with the following antibodies against PHOX2B (Clone: EPR14423; dilution: 1:100; Abcam, Cambridge, UK), MKI67 (Clone: D3B5; dilution 1:50; Cell Signaling Technology, Cambridge, UK) and cleaved Caspase 3 (Cell Signaling, 9661). Staining was performed using the Leica BOND-MAX stainer (Leica Biosystems, Germany) according to the protocol of the manufacturers. Staining for MKI67 was performed using the LabVision Autostainer 480S (Thermo Scientific) according to the protocol of the manufacturers.

BCL2 immunohistochemistry

Tissue sections were deparaffinized. Antigen unmasking was performed with Target Retrieval Solution (high pH, Agilent, K800421-2) for 10 minutes at 95°C. Sections were washed with dH_2O and then incubated in 3% hydrogen peroxide for 10 minutes. After washing steps, samples were blocked for 1 hour at room temperature (TBST/5% Normal Goat Serum). The primary antibody (anti-Bcl-2, Abcam, ab59348) was diluted (1:2000) in blocking solution and incubated over night at 4° C. Samples were then washed and incubated with SignalStain Boost Detection Reagent in a humified chamber for 30 minutes at room temperature. SignalStain DAB Chromogen substrate in SignalStain DAB Diluent was applied to the sections. After staining reaction, samples were washed in dH_2O and counterstained with hematoxylin. Sections were then dehydrated using 95% ethanol, 100% ethanol and xylene for 10 seconds each and then mounted with coverslips.

Microscopy

Microscopy was performed using a Leica DM5500 system with Cytovision (Leica, version 7.7), BZ-X810 (Keyence) microscope and ImageJ (RRID:SCR_003070), FIJI (RRID:SCR_002285, version 1.52p) software tools.

RNA sequencing

RNA isolation from fresh-frozen tissue was performed with TRIzol (Ambion). RNA concentration was measured by Qubit™ Assay (Invitrogen) and quality was assessed by 2100 Bioanalzyer (Agilent). MRNA Sequencing (Paired End, 2x100bp, 50M Reads) was conducted by Cologne Center for Genomics. Libraries were prepared using the Illumina® Stranded TruSeq® RNA sample preparation Kit. After library validation and quantification (Agilent Tape Station), equimolar amounts of library were pooled. The pool was quantified by using the Peqlab KAPA Library Quantification Kit and the Applied Biosystems 7900HT Sequence Detection System. The pool was sequenced on an Illumina NovaSeq6000 sequencing instrument with a PE100 protocol.

Quantification of RNA sequencing data was carried out using Kallisto (version 0.44.0) with parameters --bootstrap-samples 100 --bias, and a combined GRCm38/GRCh38 reference genome. Raw Kallisto transcript level read counts were transformed to gene level counts using the R package tximport (version 1.20.0). Only genes with at least 10 reads in 8 mice were used for further analysis (8 being the size of the smallest subgroup of mice).

To validate expression of *FGFR1*^{N546K} mutation in transgenic mice, the RNA sequencing data was aligned to the human GRCh37 reference genome with STAR (RRID:SCR_004463, version 2.5.3a).

Differential gene expression analysis

Normalization of Kallisto gene level read counts and differential gene expression analysis was carried out in R (version 4.2.2) using the package DESeq2 (RRID:SCR_000154, version 1.32.0) with added $\log_2$ fold-change shrinkage using the adaptive shrinkage estimator from the ashr package (version 2.2-54). P-values for each gene were computed using the Wald test and adjusted using the Benjamini-Hochberg procedure.

The Broad Institute GSEA software (version 4.2.3) was applied to the differential gene expression data to determine enrichment of the 50 MSigDB hallmark gene sets (h.all.v2023.1.Hs.symbols.gmt). Mouse gene symbols were mapped/collapsed to human orthologs using the integrated chip platform (Mouse_Gene_Symbol_Remapping_Human_Orthologs_MSigDB.v2023.1.Hs.chip). As input, preranked gene lists were generated by combining the DESeq2 $\log_2$ fold-change and p-values (sign($\log_2$ fold-change) * -$\log_{10}$(non-adjusted p-value)) into a ranking statistic. Enrichment statistic calculation was set to “weighted”. Other parameters were left to their default values.

For the purpose of visualization of gene expression values in t-SNE- and boxplots, the DESeq2 variance stabilizing transformation was applied to the normalized read counts.

TUNEL assay

Fresh frozen tumors were cut onto glass slides at 6 µm. Terminal deoxynucleotidyl transferase mediated dUTP nick end labeling (TUNEL) assay was performed using an In Situ Cell Death Detection Kit, TMR red (Roche, 12156792910) according to the manufacturer's instructions. Samples were mounted with VectaShield mounting media containing 4′,6-diamidino-2-phenylindole dihydrochloride (Vector Laboratories). Slides were scanned using a BZ-X810 (Keyence) microscope at a 20x magnification.

Reimplantation of murine tumors in NSG mice

Female NSG (NOD-scid) mice were obtained from Charles River Laboratories (RRID:SCR_003792, Strain Code: 394). Tumor pieces (approximately 1 mm^3) of genetically engineered mouse models were implanted subcutaneously into both flanks of NSG mice with Matrigel matrix (Corning, 354234). Tumor growth was monitored twice per week. Animals were randomized and treated at palpable tumor size (0.08 to 0.2 cm^3) with futibatinib (10 mg/kg, diluted in 10%DMSO + 90% solvent (20% Captisol in NaCl)) or control substance (10% DMSO + 90% solvent). Tumor volume (TV) measurement was performed with a digital caliper and volumes were calculated using the formula: TV [cm3] = length x $width^2/2$. Studies were terminated for ethical reasons when first animals reached a TV > 1.5 cm^3 or animals reached termination criteria.

Generation of patient-derived cell lines

STA-NB-1.2 cells are low-passage cultures derived from a patient with INSS stage 3, *MYCN*-amplified high-risk neuroblastoma post chemotherapy, respectively, at St. Anna Children's Cancer Research Institute. Cells were established from tumor biopsy sample by mechanic dissociation and subsequent cultivation in RPMI medium supplemented with 10% fetal calf serum, HEPES buffer, Glutamax, penicillin and streptomycin. The collection and research use of human tumor specimen was conducted according to the guidelines of the Council for International Organizations of Medical Sciences (CIOMS) and World Health Organisation (WHO) and has been approved by the Ethikkommission der Medizinischen Universität Wien. Informed consent has been obtained from the patient or parents/guardians/legally authorized

representatives. The age-adapted informed consent for the CCRI Biobank covers the use of left over materials from medically necessary surgery or biopsy, which, after completion of routine diagnostic procedures, is biobanked (EK1853/2016) and available for research purposes, including genetic analysis, that are further specified in EK1216/2018: to conduct genetic analysis and cell cultivation.

Organoid model

Freshly obtained neuroblastoma tissue was collected at the Princess Máxima Center for Pediatric Oncology, Utrecht, Netherlands. Ethics approval was granted and legal representatives signed informed consent. Organoid generation and culturing as well as drug screens were performed as previously described (75).

Generation of subcutaneous PDX model

The establishment and characterization of PDX mouse models, was previously described (76, 77). Generation of the PDX has been approved by the local ethics committee at Charité Berlin and patients have provided informed consent. In brief, fresh tumor samples were cut into pieces of 3 x 3 mm and were subcutaneously transplanted to NOD.Cg-Prkdcscid Il2rgtm1Sug/JicTac (NOG mice; Taconic, Denmark) recipient mice. Once subcutaneous (s.c.) tumors became palpable, tumor size was measured twice weekly with a digital caliper. Individual TV were calculated by the formula V= (length x width2)/2 and related to the values at the first day of treatment (relative tumor volume, RTV). The body weight (BW) of mice was determined on a regular basis and the change in body weight was taken as variable for tolerability. After 40 days, the mean tumor volume reached the indicated starting volume (146-367 mm^3). Mice were randomly assigned to control and treatment groups and treatment was started on day of randomization. The mice were treated using the following drug and treatment schedule: daily futibatinib 10 mg/kg via oral application. Placebo control mice were treated with a corresponding vehicle alone (p.o. with 10% DMSO/90% Captisol (20%) in sodium chloride). Chemotherapy treatment was performed as followed: Topotecan was administered on days 1-5 of a 21-day cycle at a dose of 0.05 mg/kg, cyclophosphamide was administered on day 1 of

the cycle at a dose of 20 mg/kg. In combination with futibatinib, futibatinib was then additionally administered daily at a dose of 20 mg/kg. At the end of the experiments, tumors were excised and one half snap frozen and stored at −80 °C for further analyses. The second half was processed for formalin fixed and paraffin embedded (FFPE) specimen. All mice used for the current study were handled in accordance with the Guidelines for the Welfare and Use of Animals in Cancer Research (78) and according to the German Animal Protection Law, approved (G0023/23) by the responsible local authorities.

Supplemental Figures

Supplemental Figure 1

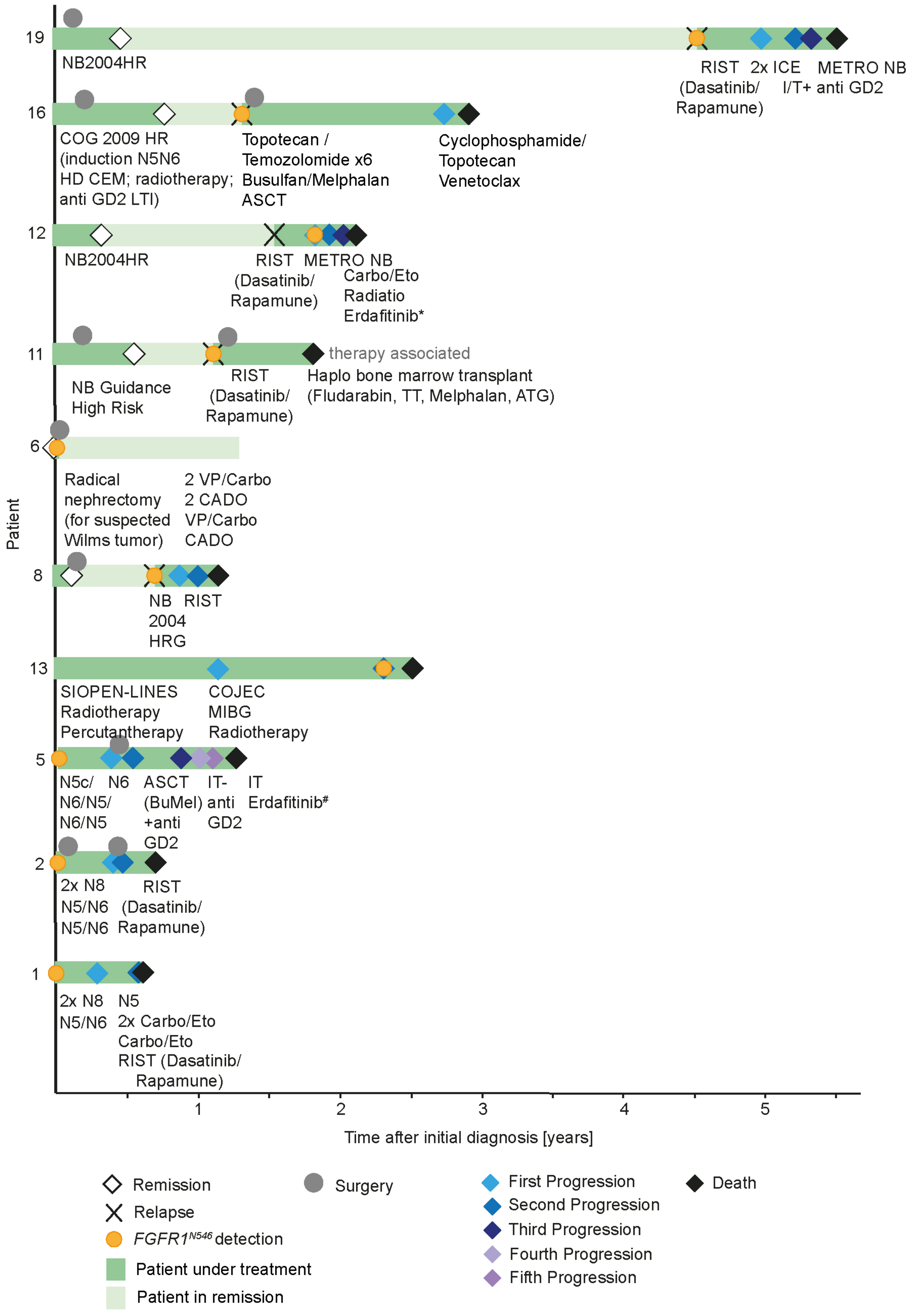

Supplemental Figure 1

Swimmer plot illustrating the course of disease of ten of the nineteen patients with *FGFR1*^{N546}-mutated neuroblastoma shown in **Figure 1**, for whom treatment information was available. Treatment of the patients is annotated. *This patient received erdafitinib treatment for 2 days before he succumbed to disease. #This patient received erdafitinib treatment for 21 days before he succumbed to disease.

Supplemental Figure 2

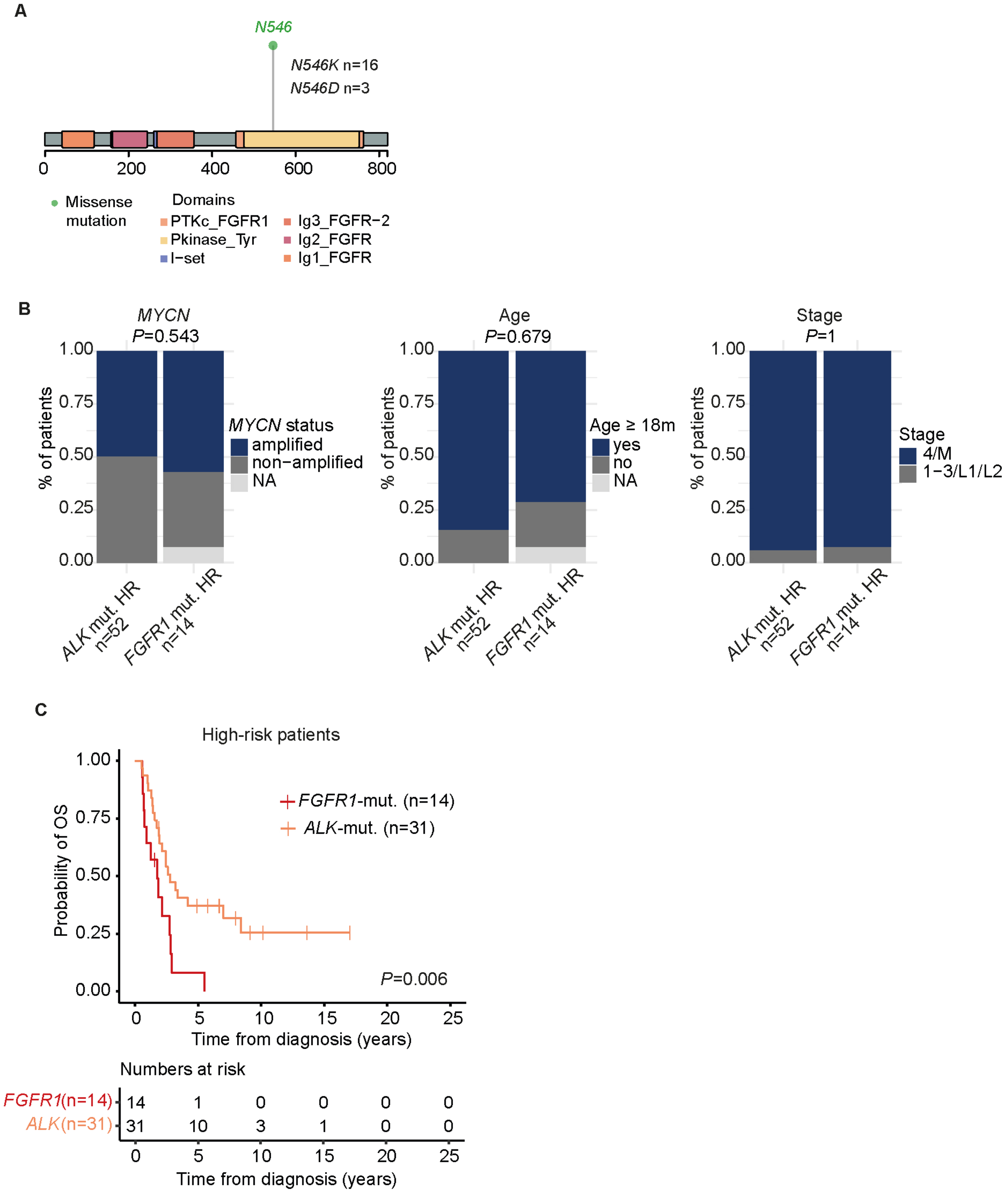

Supplemental Figure 2

A Lollipop plot illustrating the position of *N546* mutations in *FGFR1* (transcript variant NM_015850).

B Overall survival (OS) of patients with high-risk neuroblastoma bearing $FGFR1^{N546}$ (red) or *ALK* (orange) mutations. The patient cohort corresponds to the cohort shown in **Figure 1** after exclusion of patients who had received ALK inhibitor treatment. Survival curves were estimated according to Kaplan-Meier and compared with log-rank test.

C Frequencies of the risk factors *MYCN* status, age, and stage in high-risk patients with *ALK* or *FGFR1* mutations.

Supplemental Figure 3

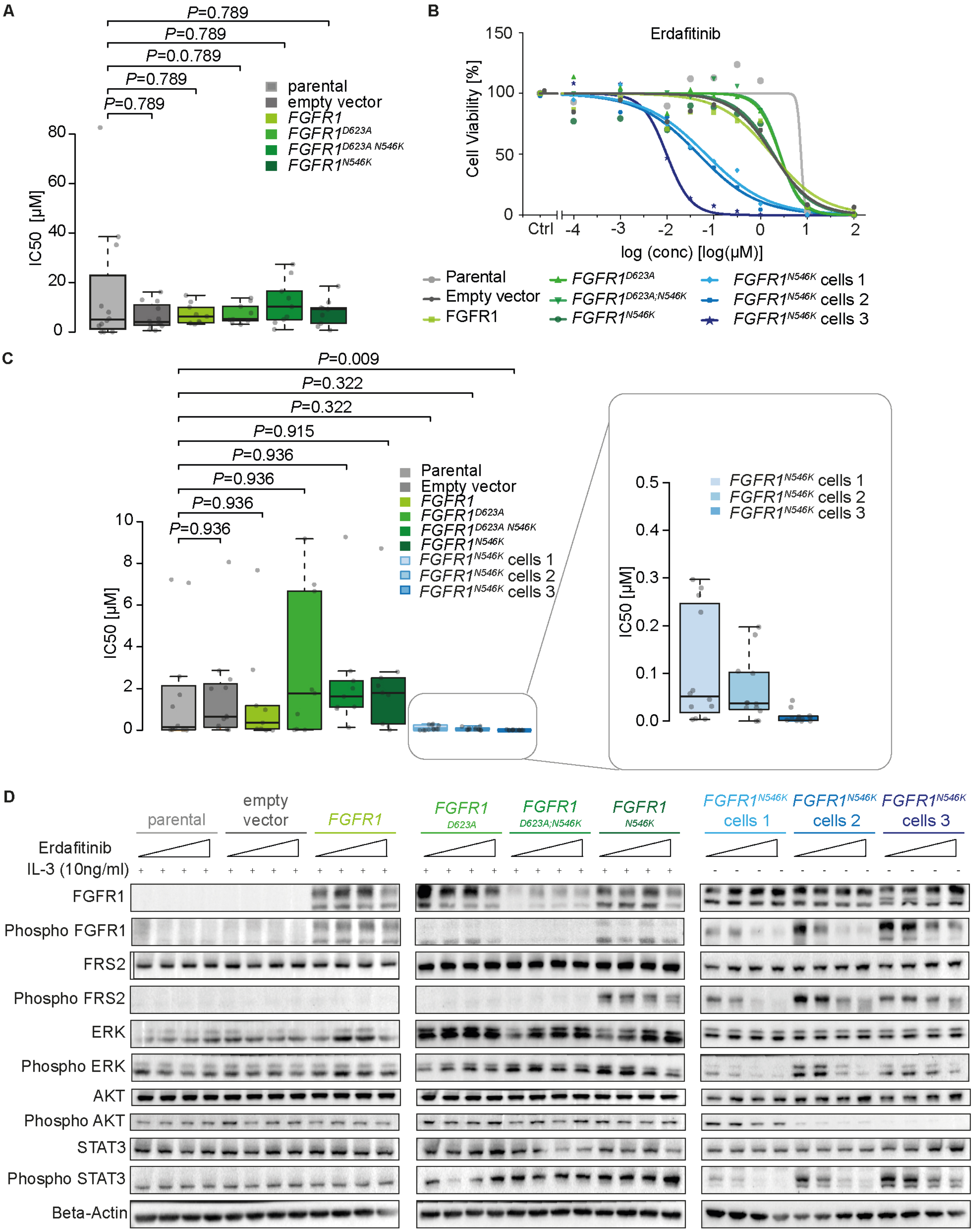

Supplemental Figure 3

A Half-maximal inhibitory concentrations (IC_{50}) of futibatinib in IL-3 dependent control Ba/F3 cells, derived from three independent experiments consisting of triplicates each. *P* values were calculated in pair-wise comparisons to Ba/F3 parental cells using a one-sided Wilcoxon rank sum test and adjusted for multiple testing using the Benjamini–Hochberg method. Same experimental data as the corresponding subplot in Main Fig. 4B is shown supplemented by statistical analysis.

B Relative cell viability of IL-3 dependent Ba/F3 cells (parental, empty vector, *FGFR1* wild-type, $FGFR1^{D623A}$, $FGFR1^{D623A;N546K}$, $FGFR1^{N546K}$) and three IL-3 independent $FGFR1^{N546K}$ transduced Ba/F3 cell clones (blue) after treatment with various concentrations of erdafitinib (0.0001 µM, 0.001 µM, 0.01 µM, 0.0398 µM, 0.0631 µM, 0.1 µM, 1 µM, 10 µM, 100 µM) and DMSO as control for 72 hours. Mean cell viabilities +/- SD of n=3 (*FGFR1* wild-type, $FGFR1^{D623A}$, $FGFR1^{D623A;N546K}$, $FGFR1^{N546K}$) or n=4 (parental, empty vector, $FGFR1^{N546K}$ IL-3 independent cells) independent experiments consisting of triplicates each are plotted.

C Half-maximal inhibitory concentrations (IC_{50}) of erdafitinib in IL-3 dependent Ba/F3 cells and three IL-3 independent $FGFR1^{N546K}$ transduced Ba/F3 cell clones, derived from three (*FGFR1* wild-type, $FGFR1^{D623A}$, $FGFR1^{D623A;N546K}$, $FGFR1^{N546K}$) or four (parental, empty vector, $FGFR1^{N546K}$ IL-3 independent cells) independent experiments consisting of triplicates each. *P* values were calculated in pair-wise comparisons to Ba/F3 parental cells using a one-sided Wilcoxon rank sum test and adjusted for multiple testing using the Benjamini–Hochberg method.

D Levels of total and phosphorylated proteins of the FGFR pathway in IL-3 dependent Ba/F3 cells (parental, empty vector, *FGFR1* wild-type, $FGFR1^{D623A}$, $FGFR1^{D623A;N546K}$, $FGFR1^{N546K}$) and three IL-3 independent $FGFR1^{N546K}$ transduced Ba/F3 cell clones (blue) after treatment with DMSO or 10, 50 or 100 nM erdafitinib.

Supplemental Figure 4

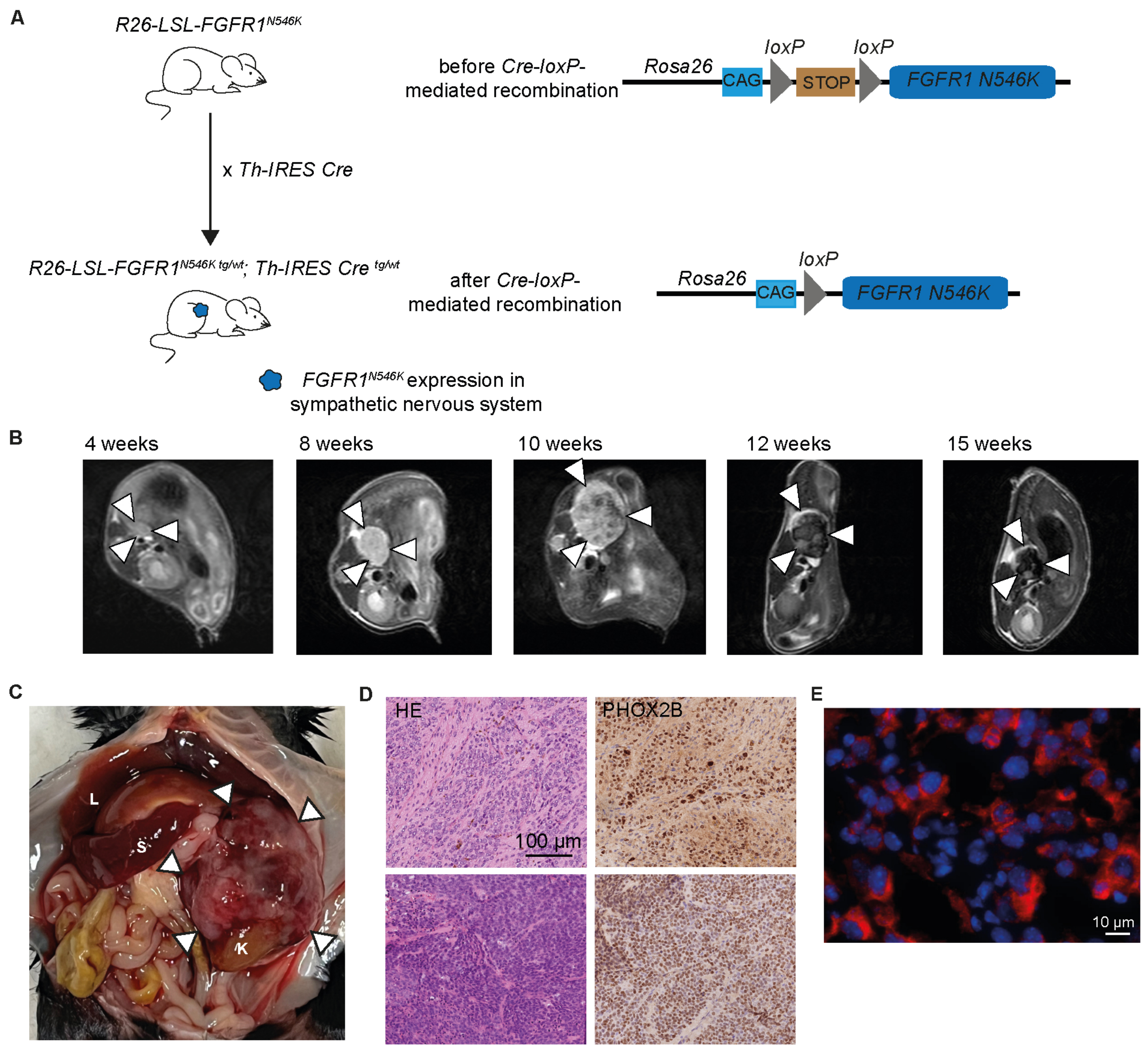

Supplemental Figure 4

A Schematic overview on the conditional expression of *FGFR1*^{N546K}. Representation of the primary targeting vector, where the *FGFR1*^{N546K} transgene (dark blue) expression is driven by the CAG promotor (light blue) but inactive due to a STOP cassette (brown). The stop cassette carries a kanamycin selection marker and is flanked by FRT sites (grey triangles), which provide target sites for the Flp recombinase that allow excision of the stop cassette and conditional activation of *FGFR1*^{N546K} transgene expression. *R26-LSL-FGFR1*^{N546K} mice were crossbred with *Th-IRES-Cre* mice to direct expression of *FGFR1*^{N546K} to cells of the sympathetic nervous system.

B Axial MRI scans of a *R26-LSL-FGFR1*$^{N546K\ tg/wt}$*;Th-IRES-Cre*$^{tg/wt}$ mouse at different timepoints are shown illustrating the development of a tumor at the age of 8 weeks (highlighted by triangles) that then showed a spontaneous regression after 10 weeks of age.

C Image of the abdominal region of *R26-LSL-FGFR1*$^{N546K\ tg/wt}$*;Th-IRES-Cre*$^{tg/wt}$ mouse at the age of 52 weeks showing a tumor (highlighted by triangles) in the adrenal region; L=liver; S=spleen, K=Kidney.

D HE staining (left) and PHOX2B (right) immunohistochemical staining of tumor sections from *R26-LSL-FGFR1*$^{N546K\ tg/wt}$*;Th-IRES-Cre*$^{tg/wt}$ mice shown in **B** (top) and **C** (bottom).

Supplemental Figure 5

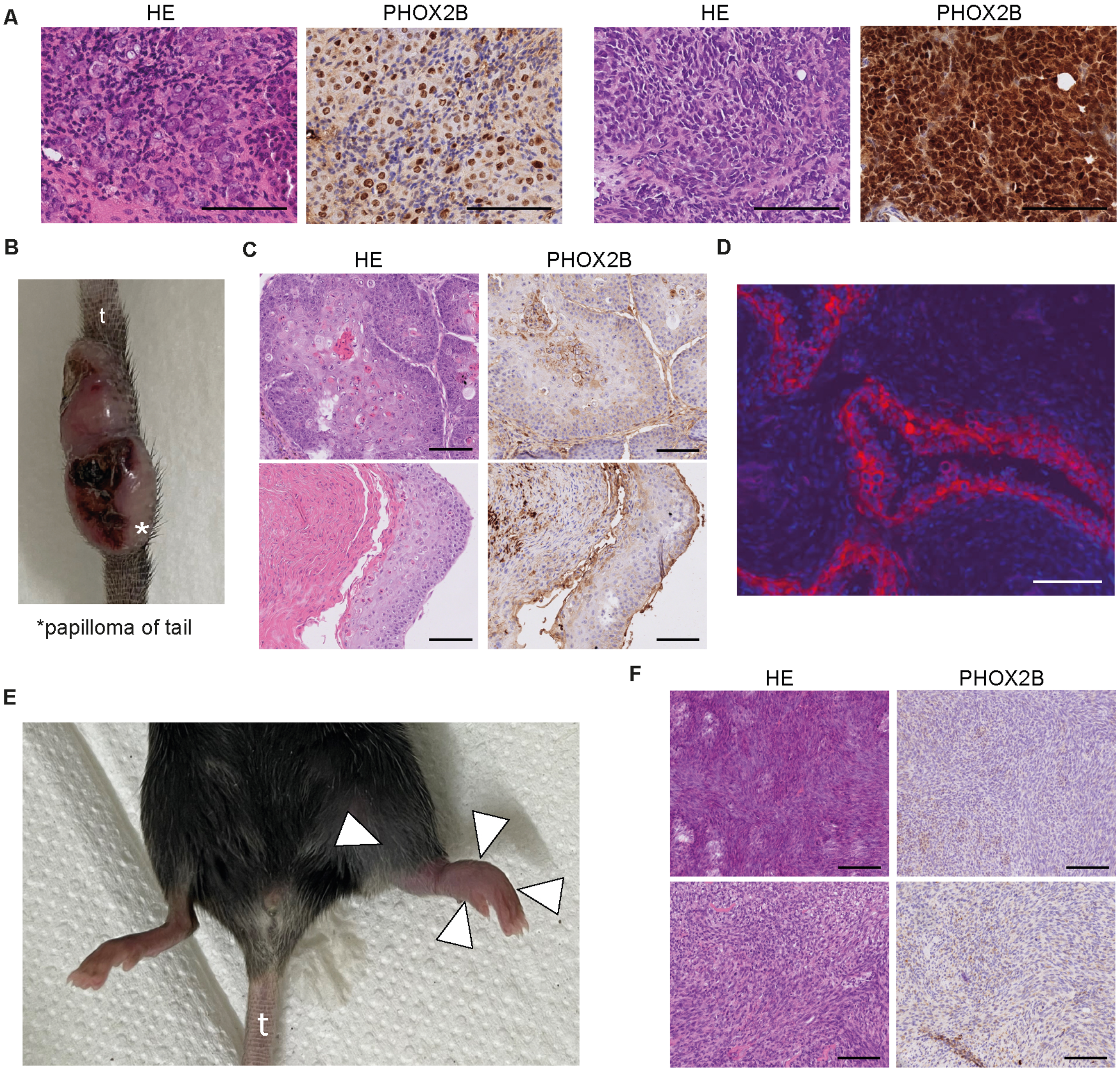

Supplemental Figure 5

A HE and PHOX2B immunohistochemical stainings of sections from adrenal glands from *R26-LSL-FGFR1*$^{N546K\ tg/wt}$*;Th-IRES-Cre*$^{tg/wt}$*;* mice (scale bar, 100 µm).

B Image of a papilloma (*) of the tail of a *R26-LSL-FGFR1*$^{N546K\ tg/wt}$*;Th-IRES-Cre*$^{tg/wt}$ mouse

C HE and PHOX2B immunohistochemical stainings of tumor sections of papillomas from *R26-LSL-FGFR1*$^{N546K\ tg/wt}$*;Th-IRES-Cre*$^{tg/wt}$*;* mice (scale bar, 100 µm).

D Immunofluorescence staining of a papilloma section of a *R26-LSL-FGFR1-N546K*$^{fl/wt}$;*Th-IRES-Cre*$^{tg/wt}$ mice shows FGFR1 expression (in red; scale bar, 100 µm). Slides were counterstained with DAPI (blue).

E Image of a sarcoma (indicated by triangles) at the leg of a *R26-LSL-FGFR1*$^{N546K\ tg/wt}$*;Th-IRES-Cre*$^{tg/wt}$ mouse.

F HE and PHOX2B immunohistochemical stainings of tumor sections of sarcomas from *R26-LSL-FGFR1*$^{N546K\ tg/wt}$*;Th-IRES-Cre*$^{tg/wt}$ mice (scale bar, 100 µm).

Supplemental Figure 6

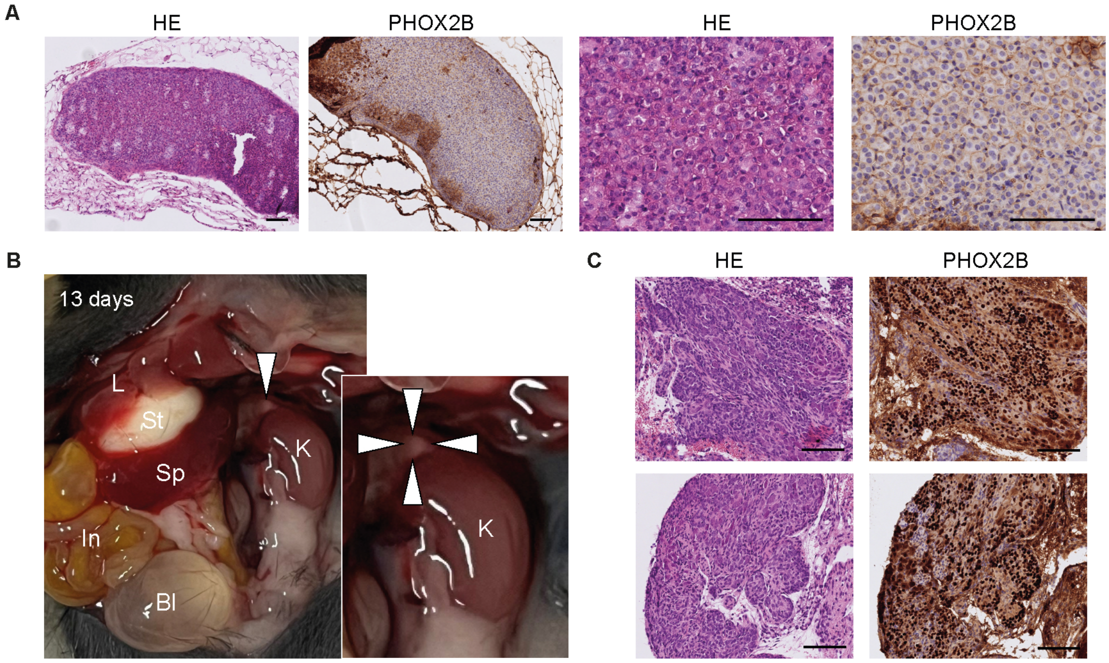

Supplemental Figure 6

A HE and PHOX2B immunohistochemical stainings of sections from adrenal glands from a *Th-ALK*$^{F1174L\ tg/wt}$ mouse at the age of one year (scale bar, 100 µm).

B Image of the abdominal region of a *Th-ALK*$^{F1174L\ tg/wt}$ mouse at the age of 13 days showing a macroscopically unremarkable adrenal gland (highlighted by triangles) in the adrenal region; L=liver; St=stomach; S=spleen, K=Kidney; In=intestine, Bl=bladder.

C HE and PHOX2B immunohistochemical stainings of sections from adrenal glands from *Th-ALK*^{F1174L} mice at the age of 13 days.

Supplemental Figure 7

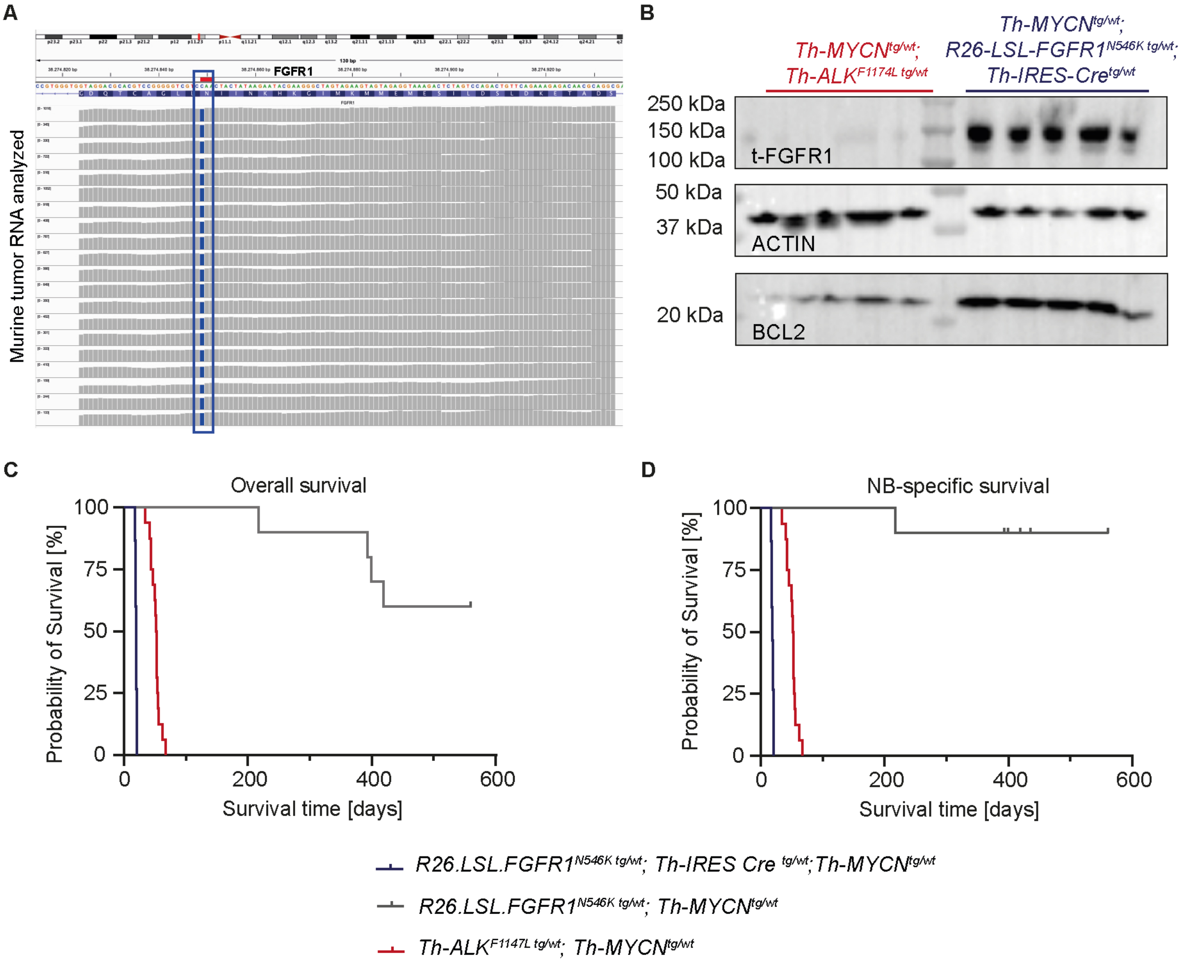

Supplemental Figure 7

A Integrative Genomics Viewer coverage plot of murine tumor RNA sequencing data illustrating expression of the mutated *FGFR1*.

B Western blot analysis of FGFR1, Bcl2 and actin in tumors of *Th-ALK*$^{F1174L\ tg/wt}$*;Th-MYCN*$^{tg/wt}$ and of *Th-MYCN*$^{tg/wt;}$ *R26-LSL-FGFR1*$^{N546K\ tg/wt}$*;Th-IRES-Cre*$^{tg/wt}$ mice.

C-D Kaplan-Meier curves showing overall survival (**C**) and disease-specific survival (**D**) of *Th-MYCN*$^{tg/wt;}$ *R26-LSL-FGFR1*$^{N546K\ tg/wt}$*;Th-IRES-Cre*$^{tg/wt}$ (dark blue; n=15), Th-*ALK*$^{F1174L\ tg/wt}$;Th-MYCN$^{tg/wt}$ (dark red; n=16) and *Th-MYCN*$^{tg/wt;}$ *R26-LSL-FGFR1*$^{N546K\ tg/wt}$ (grey, n=10). Survival of all groups differed significantly from each other (*Th-MYCN*$^{tg/wt;}$ *R26-LSL-FGFR1*$^{N546K\ tg/wt}$*;Th-IRES-Cre*$^{tg/wt}$ vs Th-*ALK*$^{F1174L\ tg/wt}$;Th-MYCN$^{tg/wt}$, *P*<0.001; *Th-MYCN*$^{tg/wt;}$ *R26-LSL-FGFR1*$^{N546K\ tg/wt}$*;Th-IRES-Cre*$^{tg/wt}$ vs *Th-MYCN*$^{tg/wt;}$ *R26-LSL-FGFR1*$^{N546K\ tg/wt}$*;* *P*<0.001; Th-*ALK*$^{F1174L\ tg/wt}$;Th-MYCN$^{tg/wt}$ vs *Th-MYCN*$^{tg/wt;}$ *R26-LSL-FGFR1*$^{N546K\ tg/wt}$, *P*<0.001). Survival curves were estimated according to Kaplan-Meier and compared with log-rank test.

Supplemental Figure 8

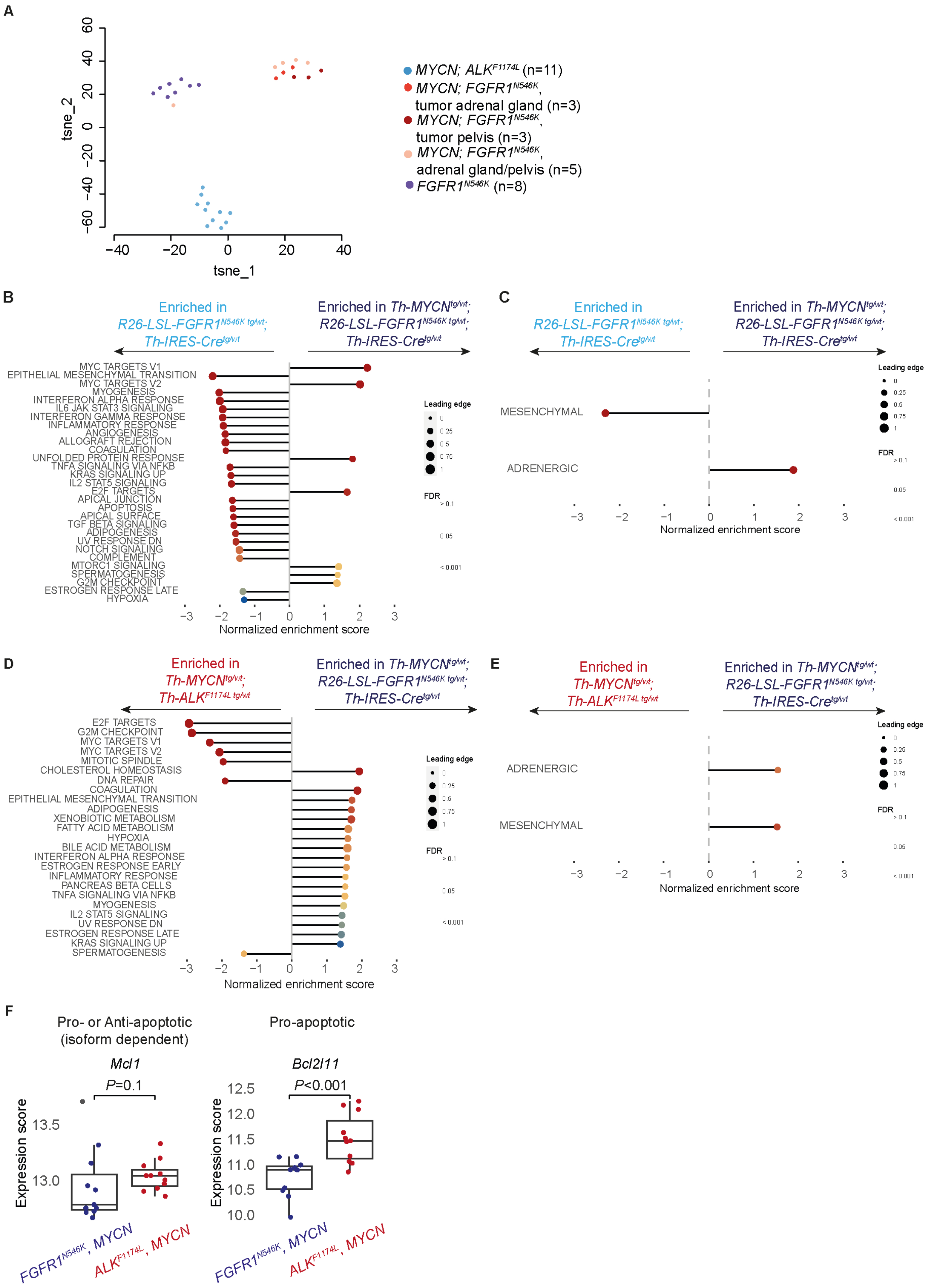

Supplemental Figure 8

A t-distributed stochastic neighbor embedding (t-SNE) plot of RNA sequencing data, determined by RNA sequencing, from *MYCN; ALK*^{F1174L}—driven, *FGFR1*^{N546K}-driven tumors and *MYCN; FGFR1*^{N546K}-driven tumors; color according to the genotype and tumor localization.

B Results of gene set enrichment analysis (GSEA) of early *FGFR1*$^{N546K-}$driven *vs MYCN; FGFR1*^{N546K}-driven tumors. Lollipop charts show the normalized enrichment scores, dot color indicate the significance level, and dot size the leading edge (size of the gene subset (in %) contributing most to the gene set enrichment score.

C Results of mesenchymal and adrenergic gene set enrichment analysis (GSEA) of *FGFR1*^{N546K} driven vs *MYCN;FGFR1*^{N546K} driven tumors. Lollipop charts show the normalized enrichment scores, dot color indicate the significance level, and dot size the leading edge (size of the gene subset (in %) contributing most to the gene set enrichment score.

D Results of gene set enrichment analysis (GSEA) of *MYCN;ALK*^{F1174L} driven *vs MYCN; FGFR1*^{N546K} driven tumors. Lollipop charts show the normalized enrichment scores, dot color indicate the significance level, and dot size the leading edge (size of the gene subset (in %) contributing most to the gene set enrichment score.

E Results of mesenchymal and adrenergic gene set enrichment analysis (GSEA) of *MYCN;ALK*^{F1174L} driven *vs MYCN;FGFR1*^{N546K} driven tumors. Lollipop charts show the normalized enrichment scores, dot color indicate the significance level, and dot size the leading edge (size of the gene subset (in %) contributing most to the gene set enrichment score.

F Expression levels of *Mcl1* and *Bcl2l11* in tumors of *R26-FGFR1*$^{N546K/wt}$*;Th-IRES-Cre*$^{tg/wt}$*;Th-MYCN*$^{tg/wt}$ and *Th-ALK*$^{F1174L/wt}$*;Th-MYCN*$^{tg/wt}$ mice, as determined by RNA sequencing.

Supplemental Figure 9

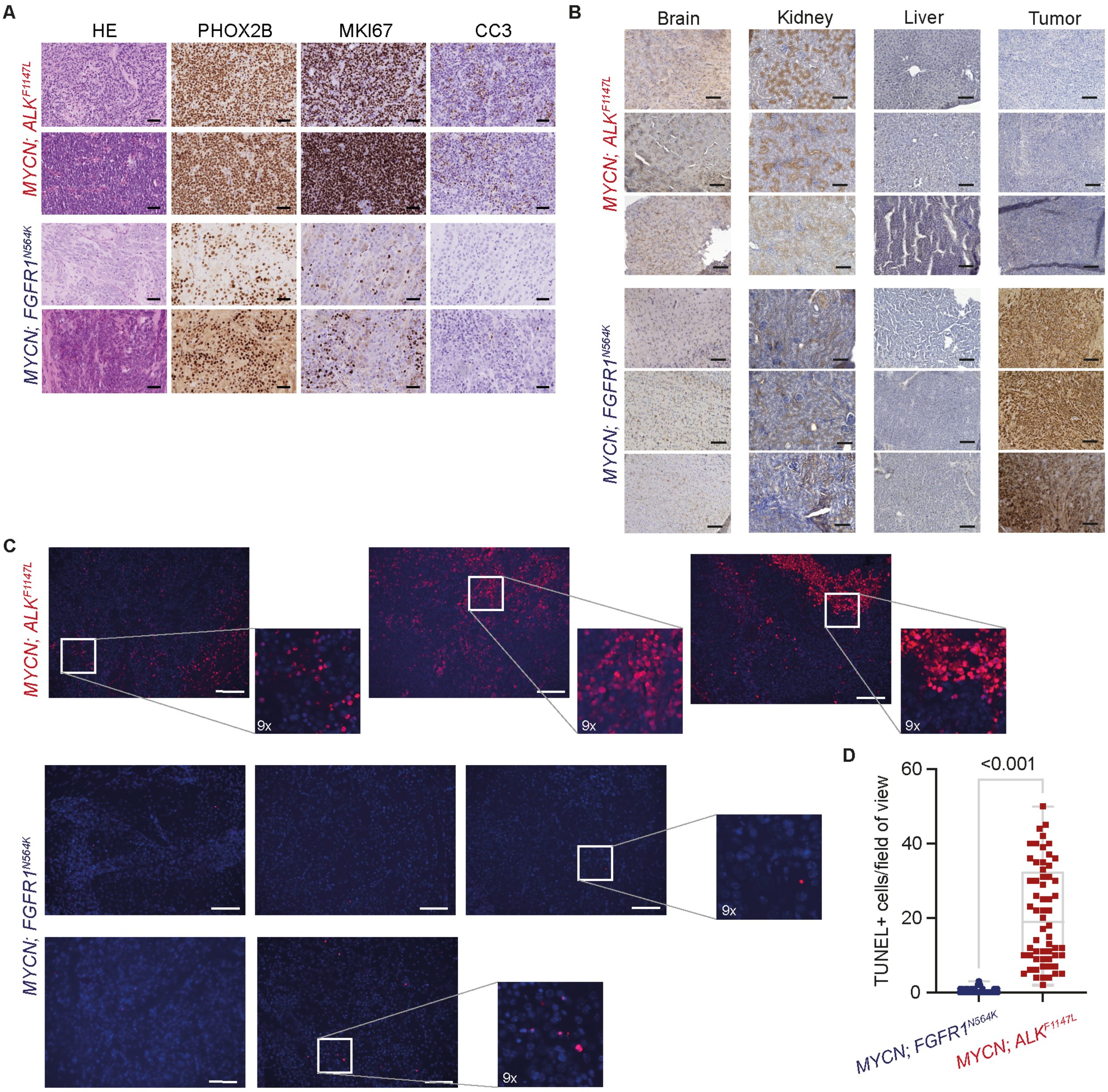

Supplemental Figure 9

A HE staining, and PHOX2B, Ki67, and cleaved caspase 3 (CC3) immunohistochemical staining of tumor sections from two *Th-MYCN;Th-ALK*^{F1174L} (top) and two *R26-LSL-FGFR1-N546K*$^{fl/wt}$*;Th-IRES-Cre*$^{tg/wt}$*; Th-MYCN*$^{tg/wt}$ mice (bottom, scale bar, 50 µm).

B BCL2 immunohistochemical staining of tumor and normal tissue sections from three *Th-MYCN;Th-ALK*^{F1174L} and three *R26-LSL-FGFR1-N546K*$^{fl/wt}$*;Th-IRES-Cre*$^{tg/wt}$*;Th-MYCN*$^{tg/wt}$ mice. Scale bar, 100 µm.

C TUNEL staining of tumor sections from three *Th-MYCN;Th-ALK*^{F1174L} (top) and five *R26-LSL-FGFR1-N546K*$^{fl/wt}$*;Th-IRES-Cre*$^{tg/wt}$*;Th-MYCN*$^{tg/wt}$ mice (bottom, scale bar, 100 µm).

D Comparison of TUNEL-positive cells per field of view in tumor sections from three *Th-MYCN;Th-ALK*^{F1174L} and five *R26-LSL-FGFR1-N546K*$^{fl/wt}$*;Th-IRES-Cre*$^{tg/wt}$*;Th-MYCN*$^{tg/wt}$ mice.

Supplemental Figure 10

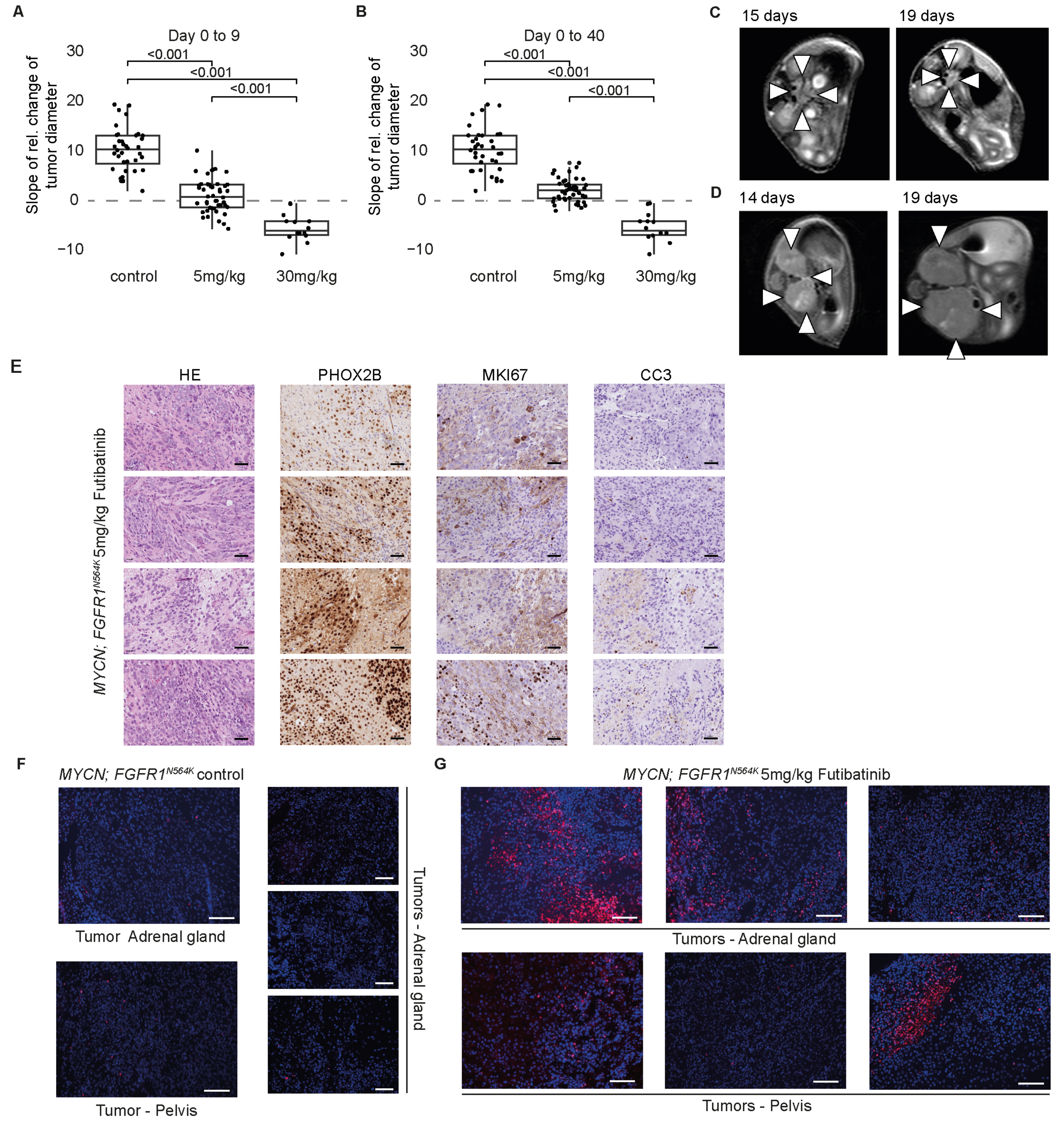

Supplemental Figure 10

A-B Comparison of the dynamics of tumor diameters of control and futibatinib treated *R26-LSL-FGFR1-N546K*$^{fl/wt}$*;Th-IRES-Cre*$^{tg/wt}$*;Th-MYCN*$^{tg/wt}$ mice, considering the dynamics of each individual tumor (up to 4 tumors per mouse). To compare the development of tumor diameters over time between treatment conditions of mice, a linear model and their slopes were calculated for each mouse using R. Treatment groups were compared using two-sided Wilcoxon rank sum test. Analysis was performed for the period over which data for all mice were available (day 0 to 9; **A**), and the maximum treatment period (day 0 to 40; **B**).

C MRI scans at the age of 15 and 19 days of a *R26-LSL-FGFR1-N546K*$^{fl/wt}$*;Th-IRES-Cre*$^{tg/wt}$*; Th-MYCN*$^{tg/wt}$ mouse treated with 30 mg/kg futibatinib daily. The tumor is indicated by triangles.

D MRI scans at the age of 14 and 19 days of a control (CMC-Na) treated *R26-LSL-FGFR1-N546K*$^{fl/wt}$*;Th-IRES-Cre*$^{tg/wt}$*;Th-MYCN*$^{tg/wt}$ mouse. Tumors are indicated by triangles.

E HE staining, PHOX2B, Ki67 and cleaved caspase 3 (CC3) immunohistochemical staining of tumor sections from *R26-LSL-FGFR1-N546K*$^{fl/wt}$*;Th-IRES-Cre*$^{tg/wt}$*;Th-MYCN*$^{tg/wt}$ mice treated with 5mg/kg futibatinib daily (bottom, scale bar, 50 µm).

F-G TUNEL staining of tumor sections from *FGFR1-N546K*$^{fl/wt}$*;Th-IRES-Cre*$^{tg/wt}$*;Th-MYCN*$^{tg/wt}$ mice treated with the control substance (**F**) or 5 mg/kg futibatinib daily (**G**; scale bar, 100 µm).

Supplemental Figure 11

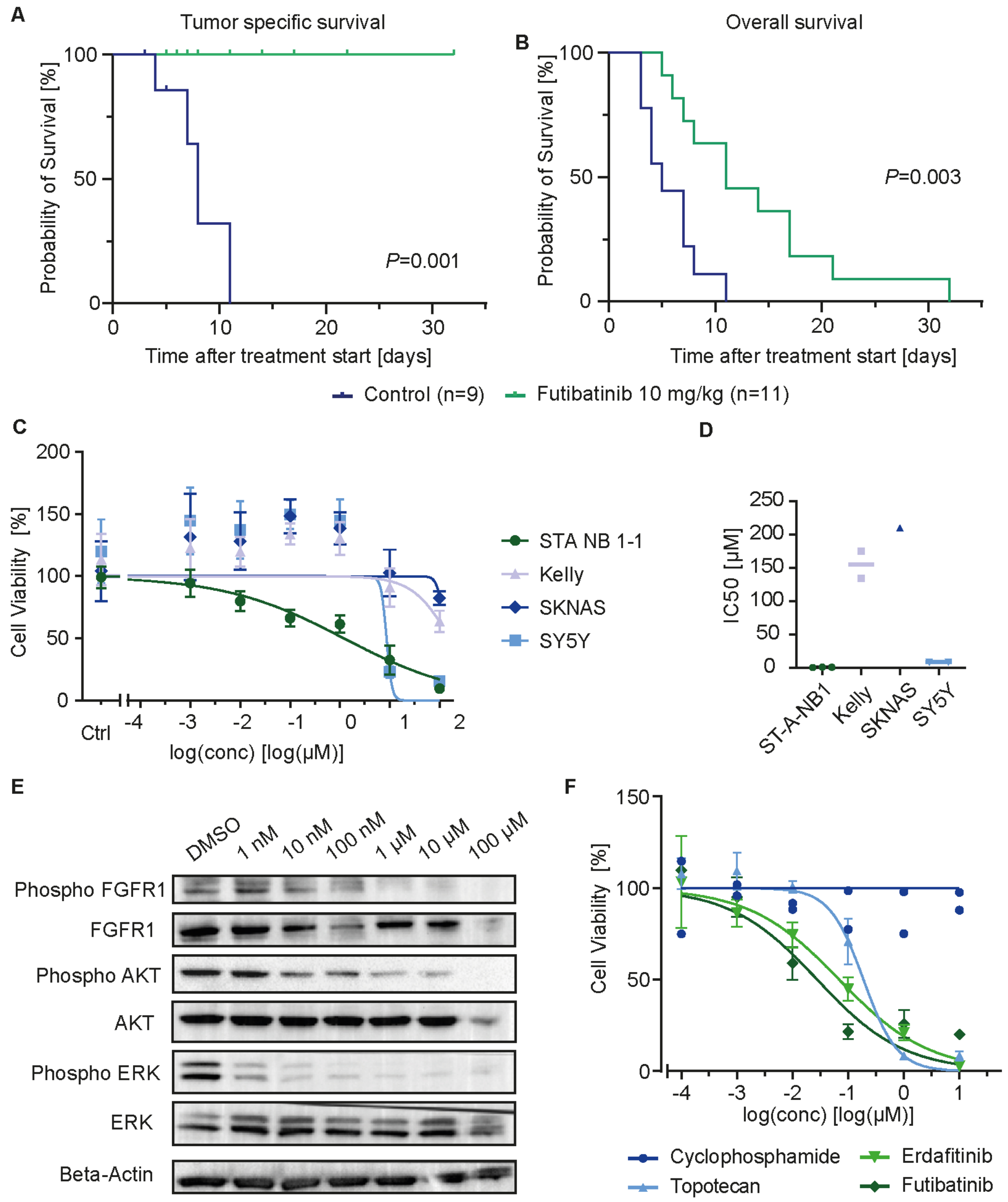

Supplemental Figure 11

A Tumor-specific survival of NSG mice with reimplanted *MYCN;FGFR1*^{N546K} driven murine tumors. Survival curves were estimated according to Kaplan-Meier and compared with log-rank test.

B Overall survival of NSG mice with reimplanted *MYCN;FGFR1*^{N546K} driven murine tumors. Survival curves were estimated according to Kaplan-Meier and compared with log-rank test.

C Relative cell viability of an *FGFR1*^{N546K} mutant patient-derived cell line (STA NB 1-1) and *FGFR1* wild-type neuroblastoma cell lines (Kelly, SKNAS, SY5Y) after exposure to increasing concentrations of futibatinib (0.0001 µM, 0.001 µM, 0.01 µM, 0.1 µM, 1 µM, 10 µM, 100 µM) or DMSO as control for 72 hours.

D Half-inhibitory concentrations (IC_{50}) of futibatinib in neuroblastoma cell lines, calculated from the cell viability assay shown in **C**.

E Levels of total and phosphorylated proteins of the FGFR pathway in an *FGFR1*^{N546K} mutant patient-derived cell line (STA NB 1-1) after treatment with DMSO or increasing concentrations of futibatinib.

F Relative cell viability of an *FGFR1*^{N546K} mutant patient-derived organoid after treatment with increasing concentrations of futibatinib, erdaftinib, topotecan or cyclophosphamide (0.0001 µM, 0.001 µM, 0.01 µM, 0.1 µM, 1 µM, 10 µM, 100 µM), or DMSO as control for 72 hours.

Supplemental Figure 12

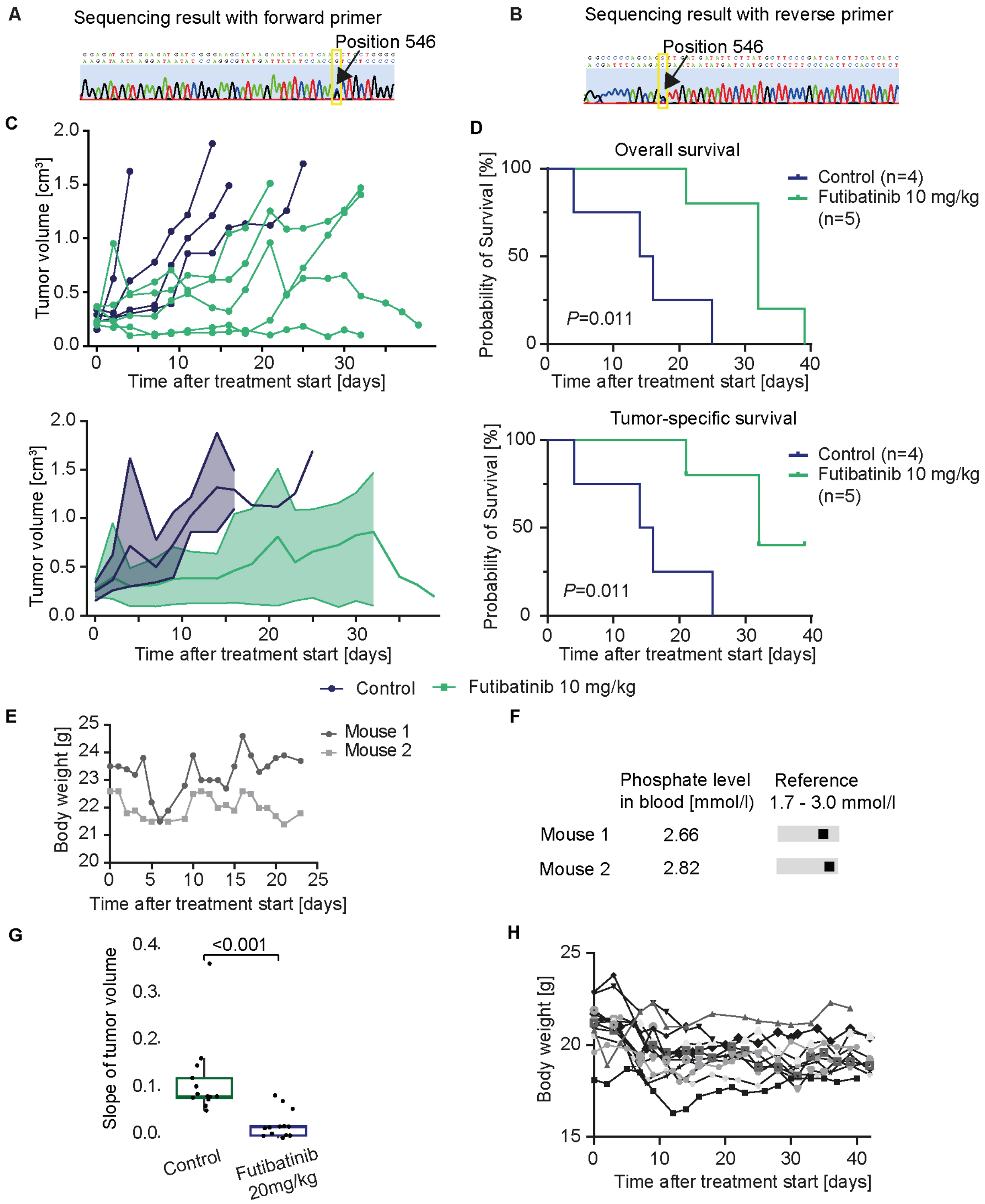

Supplemental Figure 12

A Sequencing result (forward primer) of DNA extracted from the tumor used for reimplantation of the patient-derived xenograft model.

B Sequencing result (reverse primer) of DNA extracted from the tumor used for reimplantation of the patient-derived xenograft model.

C Absolute tumor volumes of individual tumors (top) and mean and range (bottom) of tumors of an *FGFR1* mutant patient-derived xenograft model treated with control substance (n=4, blue) or 10 mg/kg futibatinib daily (n=5, green).

D Overall survival (top) and tumor-specific survival (bottom) of the patient-derived xenograft model treated with control (blue) or 10 mg/kg futibatinib (green). Survival curves were estimated according to Kaplan-Meier and compared with log-rank test.

E Body weight of NOG mice treated with 10 mg/kg futibatinib daily over three weeks.

F Phosphate levels in the blood of the mice treated with 10 mg/kg futibatinib over three weeks.

G Comparison of the dynamics of tumor volumes of control and futibatinib treated mice bearing a patient derived neuroblastoma xenograft. To compare the development of tumor volumes over time between treatment conditions of mice, a linear model and their slopes were calculated for each mouse using R. Treatment groups were compared using two-sided Wilcoxon rank sum test.

H Body weight of NOG mice treated with 20 mg/kg futibatinib daily (n=3).

Supplemental Figure 13

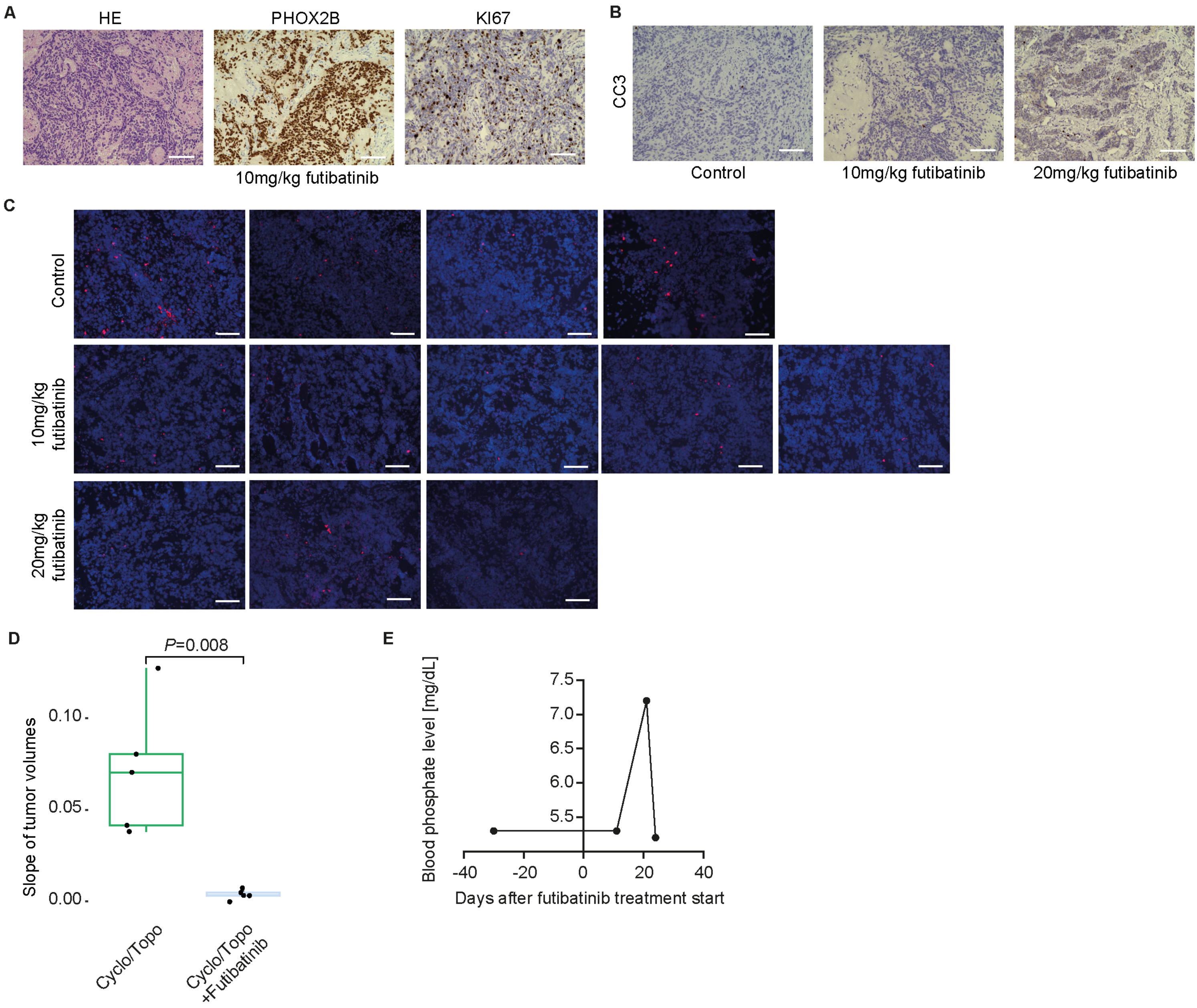

Supplemental Figure 13

A HE staining, PHOX2B and Ki67 immunohistochemical staining of tumor sections from *FGFR1*-mutated PDX model treated 10 mg/kg futibatinib (scale bar, 100 µm).

B Cleaved caspase 3 (CC3) immunohistochemical staining of tumor sections from the PDX model treated with the control substance, 10 mg/kg or 20 mg/kg futibatinib (scale bar, 100 µm)

C TUNEL staining of tumor sections from the PDX model treated with the control substance, 10 mg/kg or 20 mg/kg futibatinib (scale bar, 100 µm).

D Comparison of the dynamics of tumor volumes of mice bearing a patient derived neuroblastoma xenograft treated with cyclophosphamide/topotecan alone or in combination with futibatinib. To compare the development of tumor volumes over time between treatment conditions of mice, a linear model and their slopes were calculated for each mouse using R. Treatment groups were compared using two-sided Wilcoxon rank sum test.

E Blood phosphate levels of the patient shown in **Figure 6** before and during futibatinib treatment.
