# Supplementary material for: Mutated FGFR1 is an oncogenic driver and therapeutic target in high-risk neuroblastoma
Source: J Clin Invest. 2026 Feb 12;136(7):e189152. doi: 10.1172/JCI189152 (PMC13038208; doi:10.1172/JCI189152)
Supplement: Unedited blot and gel images [file jci-136-189152-s257.pdf]

Full unedited gels for Figure 3B

FGFR1	Phospho-FGFR1
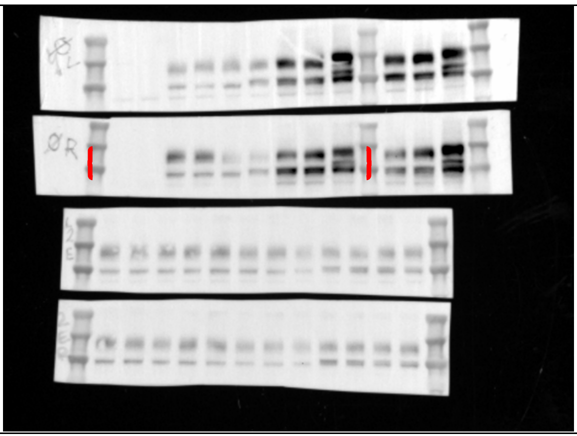	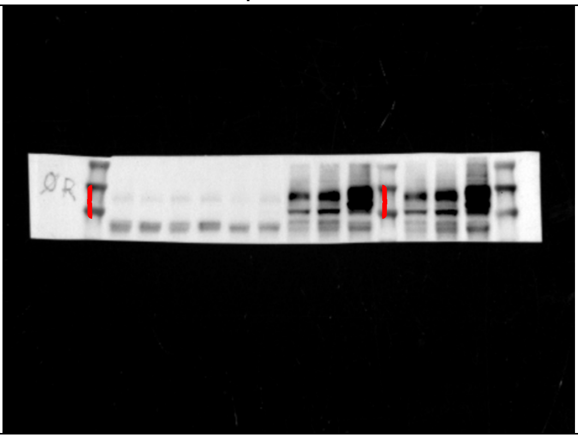
FRS2	Phospho-FRS2
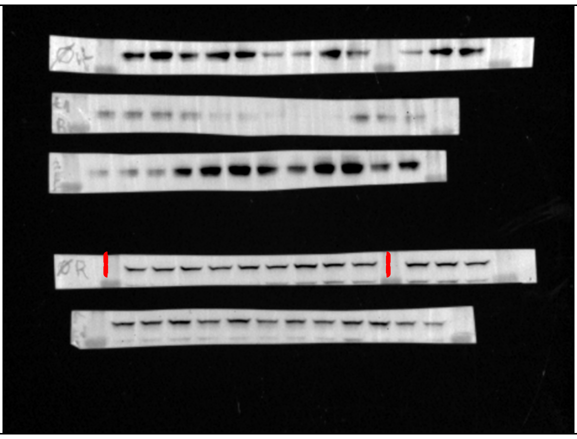	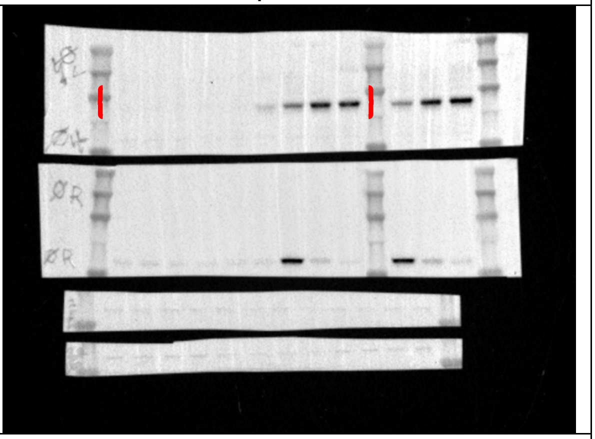
ERK	Phospho-ERK
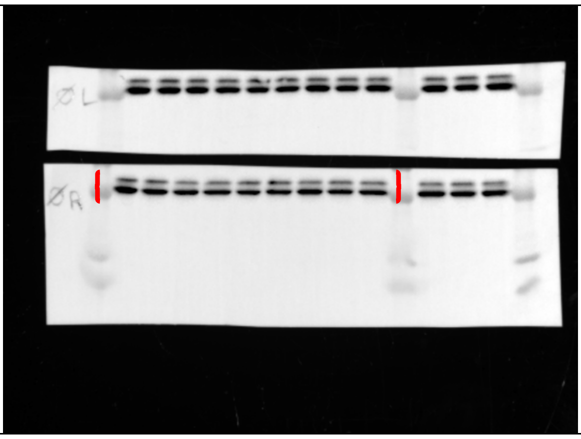	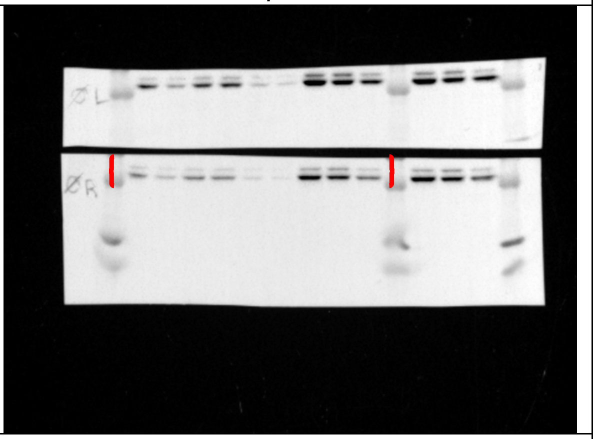
AKT	Phospho-AKT
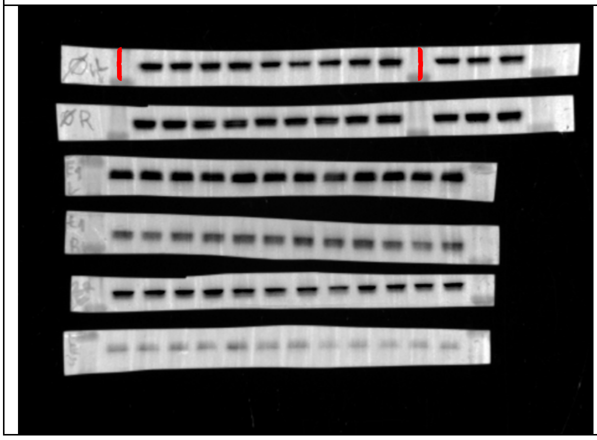	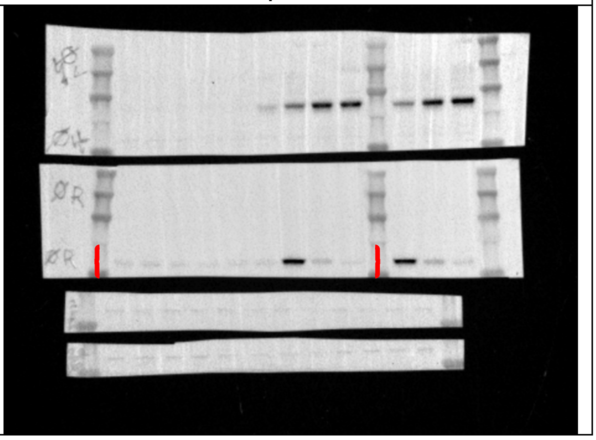

STAT3	Phospho-STAT3
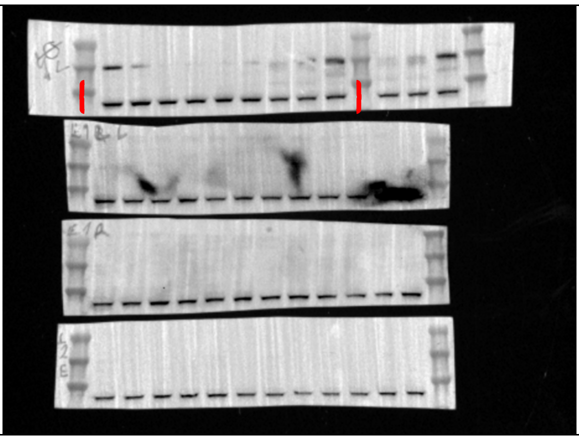	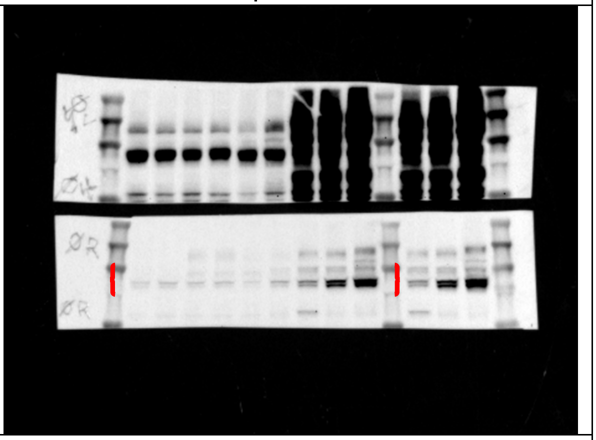
Beta-Actin	
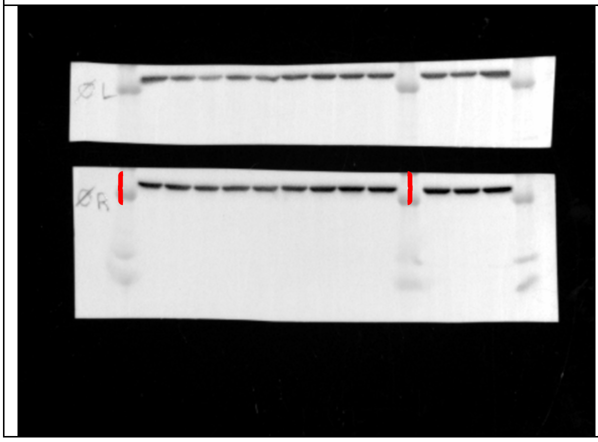	

Full unedited gels for Figure 4C

FGFR1	Phospho-FGFR1
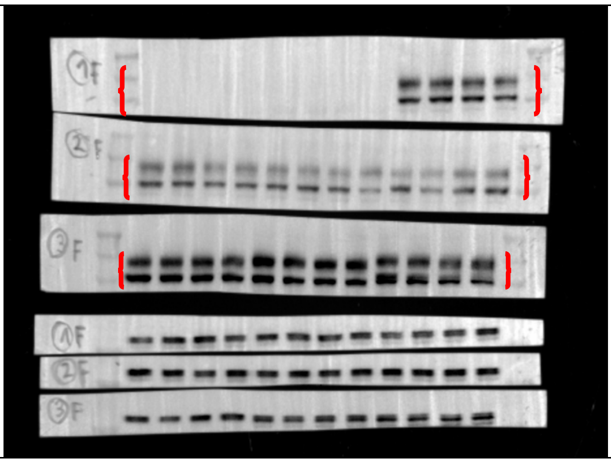	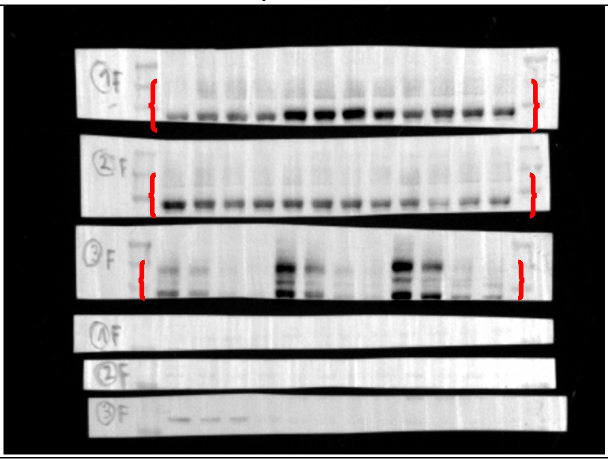
FRS2	Phospho-FRS2
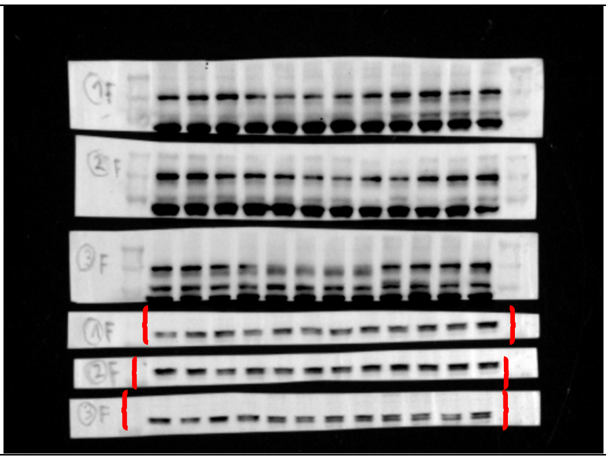	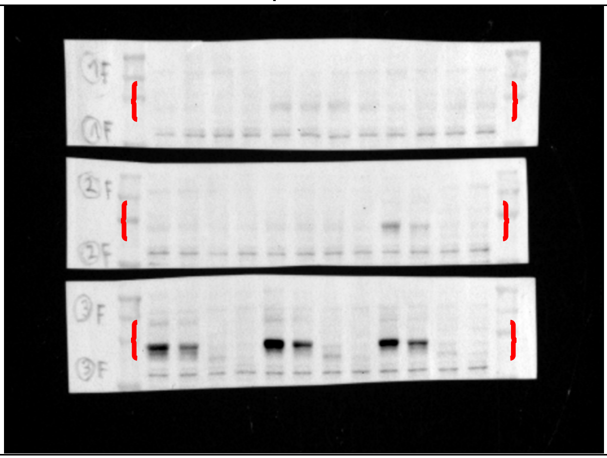
ERK	Phospho-ERK
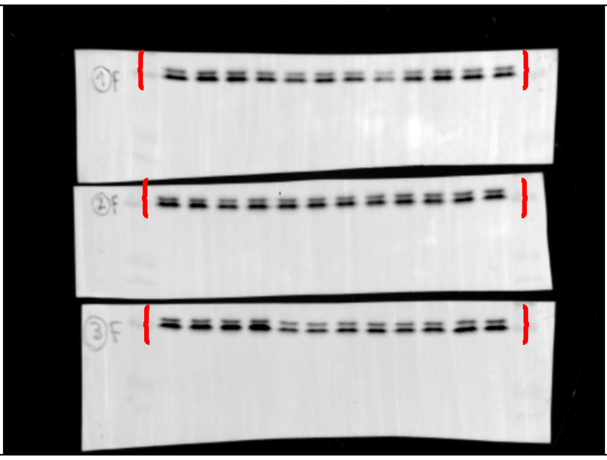	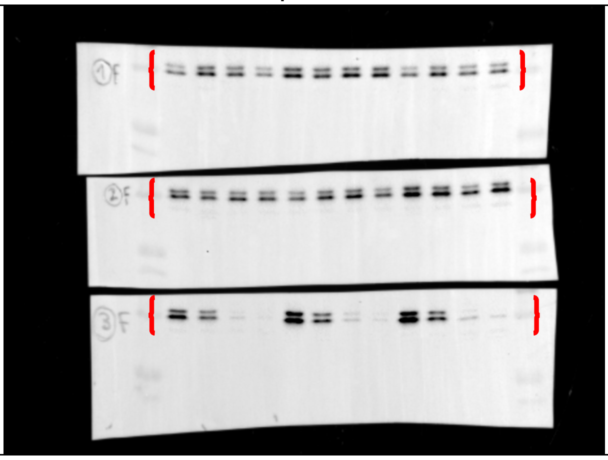
AKT	Phospho-AKT
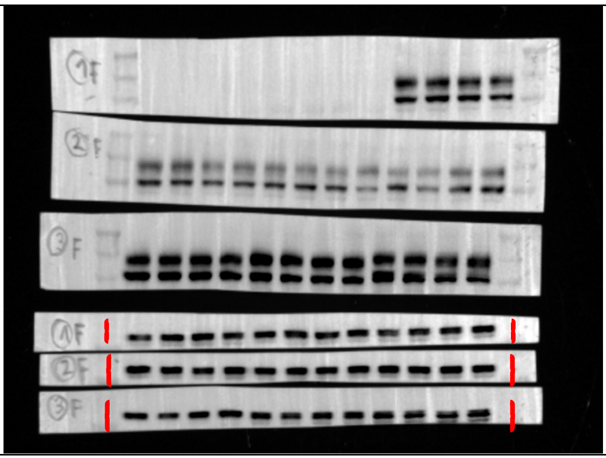	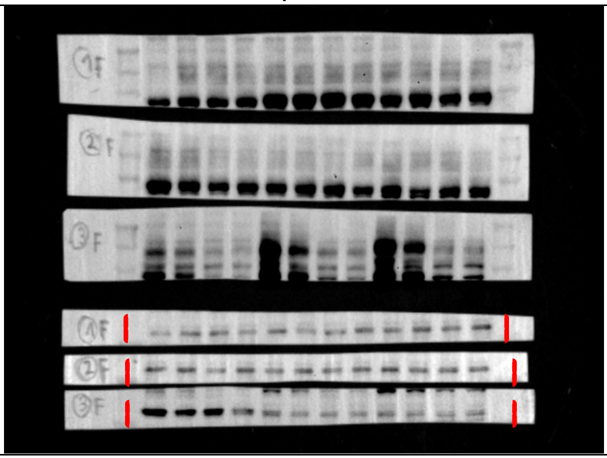

STAT3	Phospho-STAT3
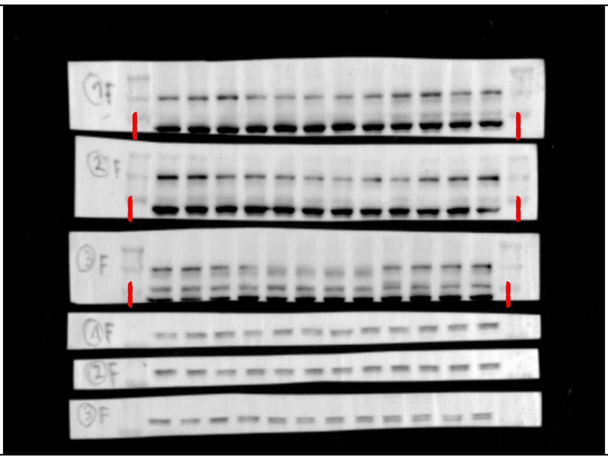	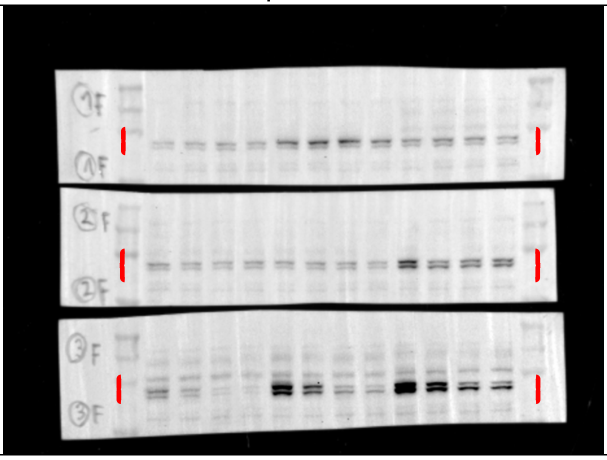
Beta-Actin	
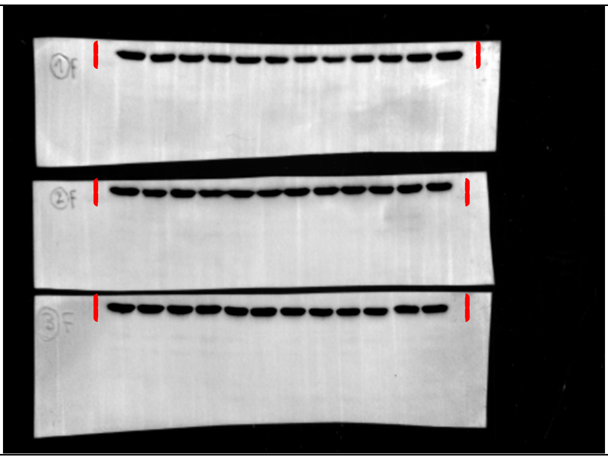	

Full unedited Gels for Supplemental Figure 2D

FGFR1	Phospho-FGFR1
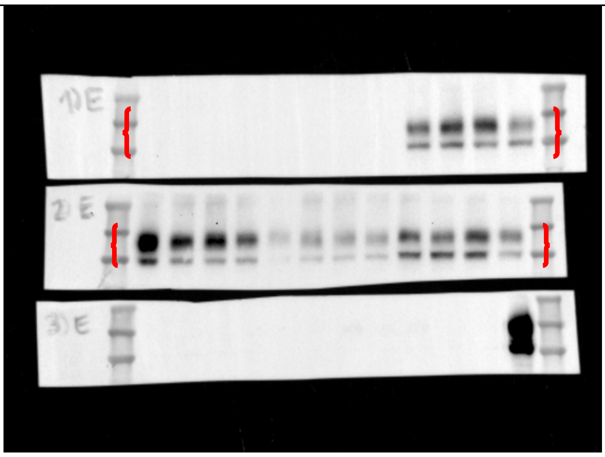	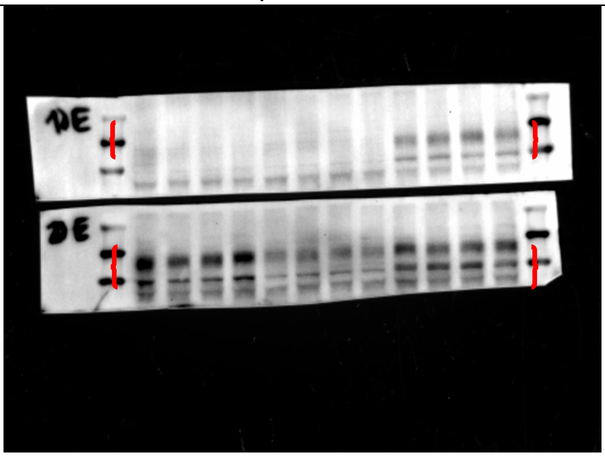
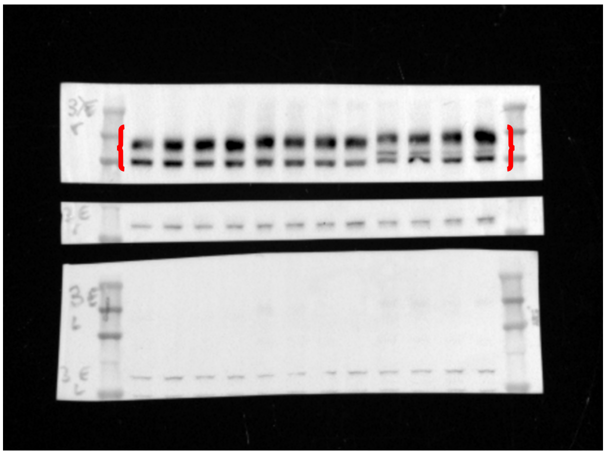	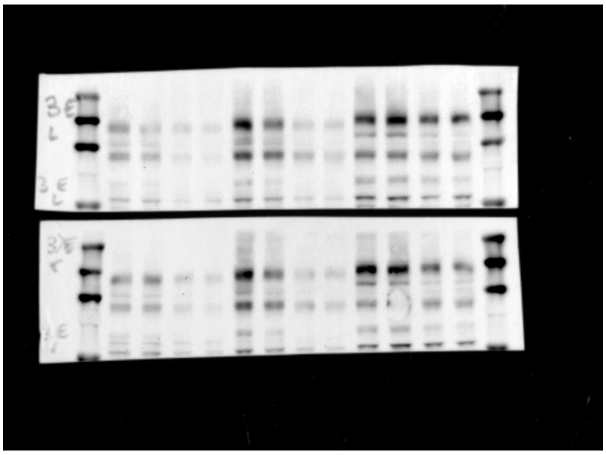
	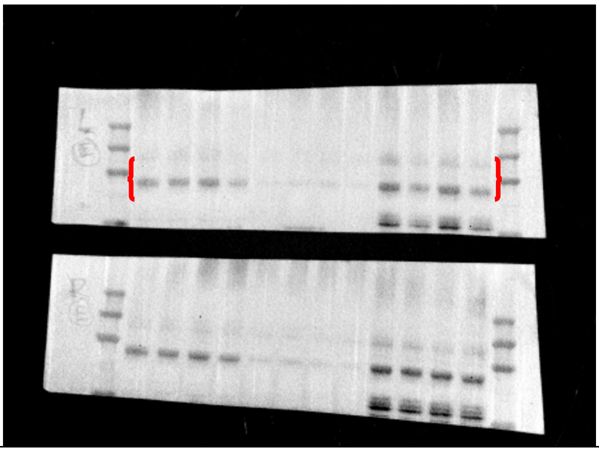
FRS2	Phospho-FRS2

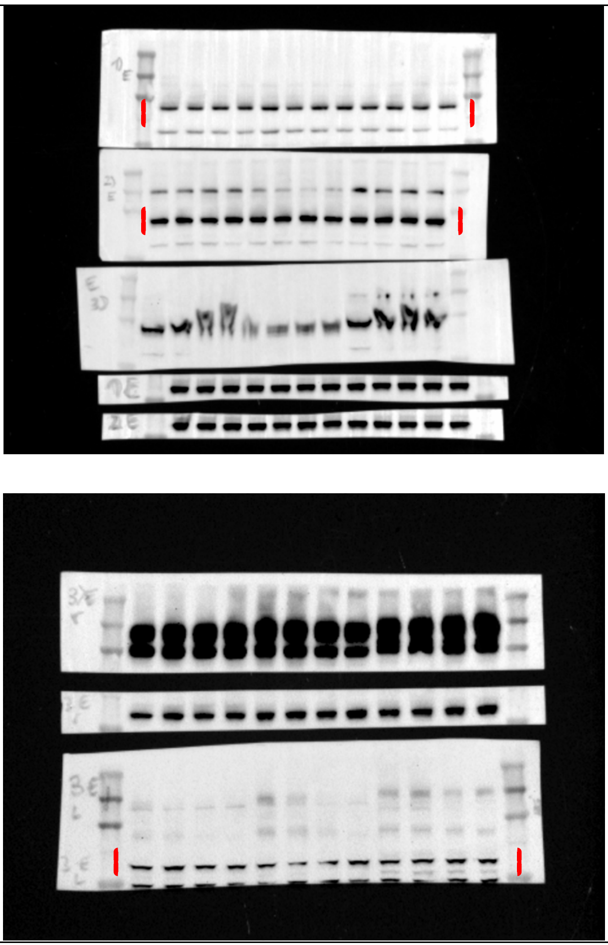	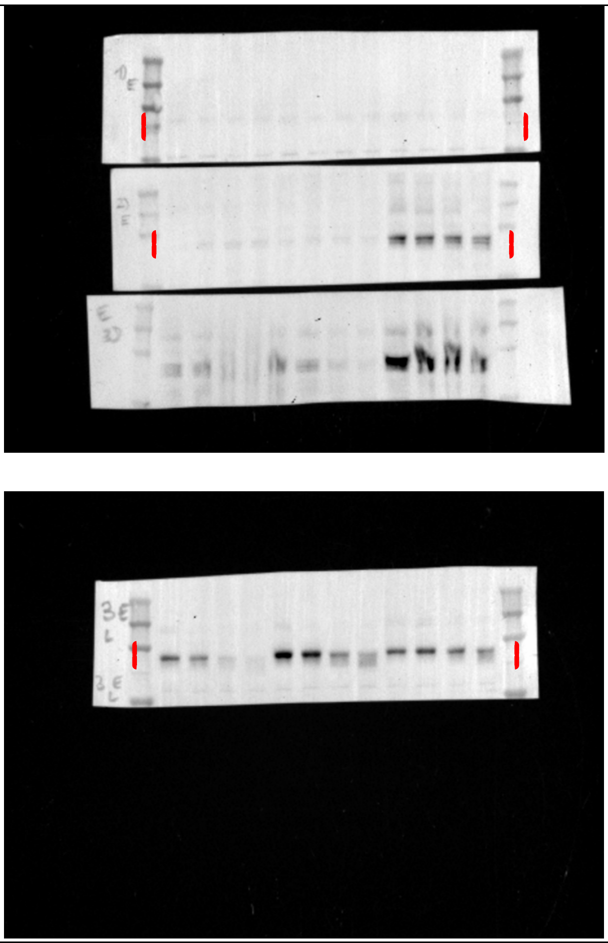
ERK	Phospho-ERK
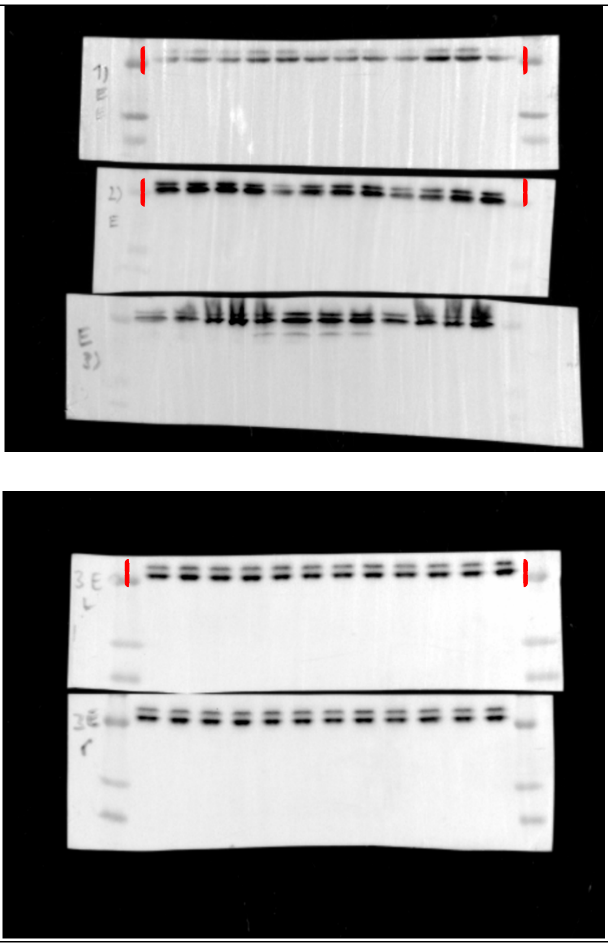	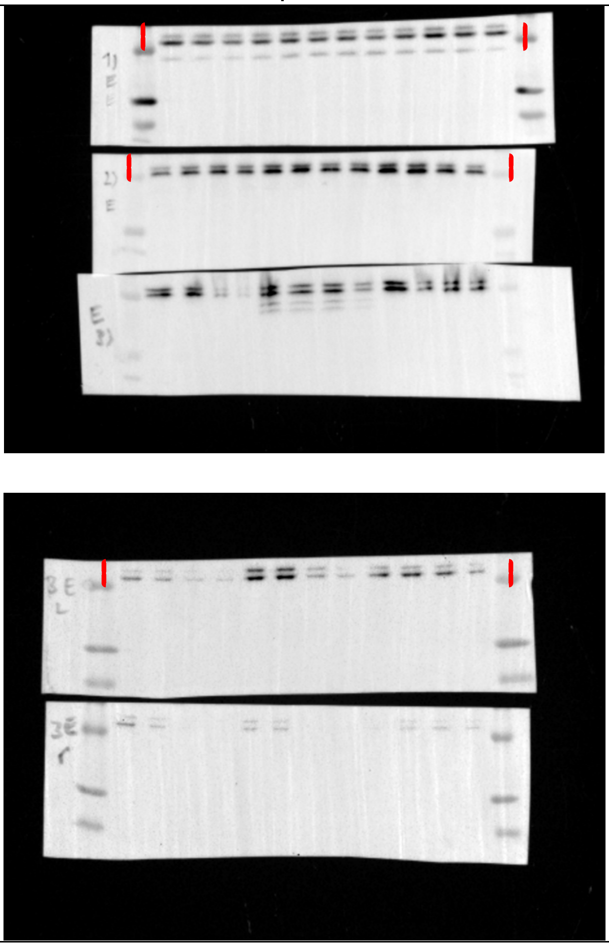
AKT	Phospho-AKT

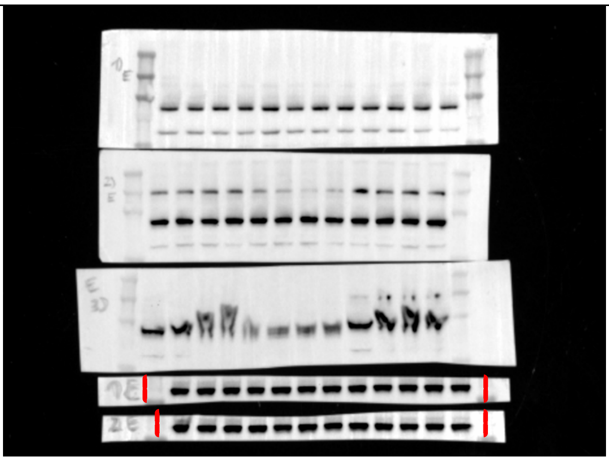 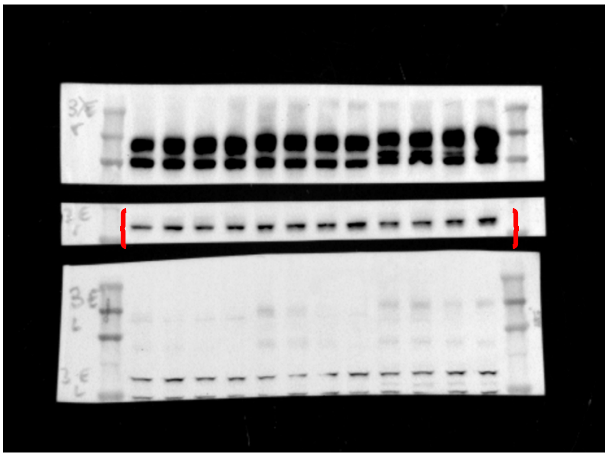	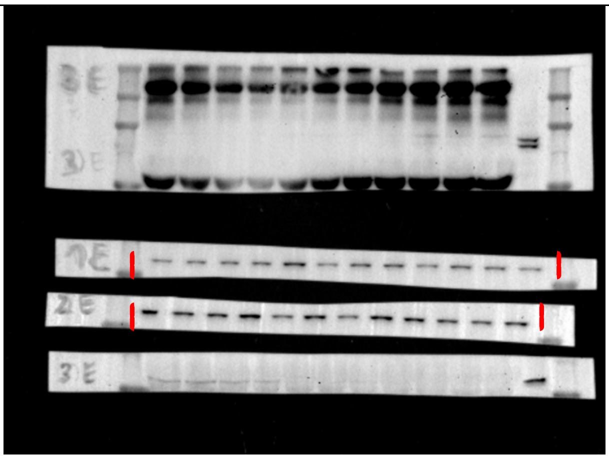 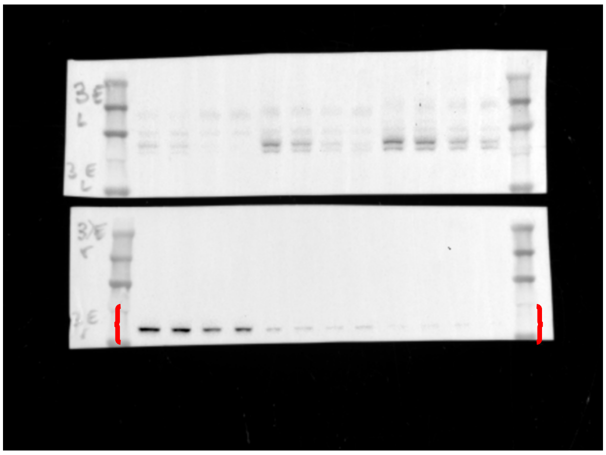
STAT3	Phospho-STAT3
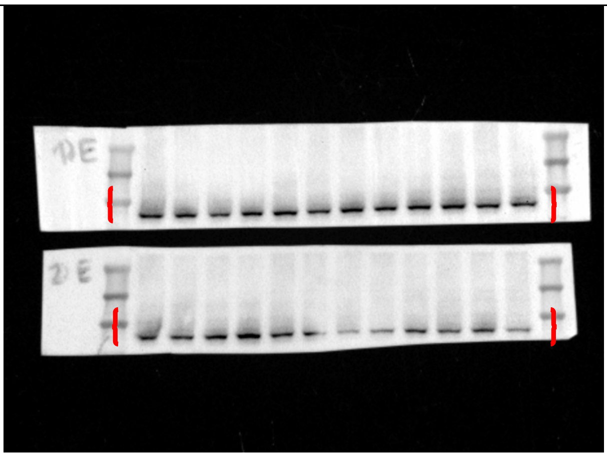 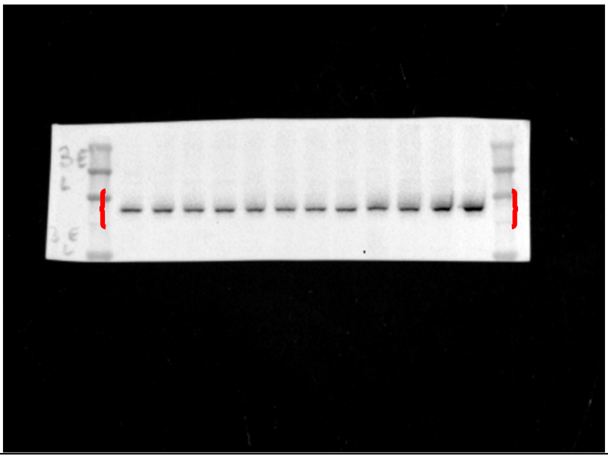	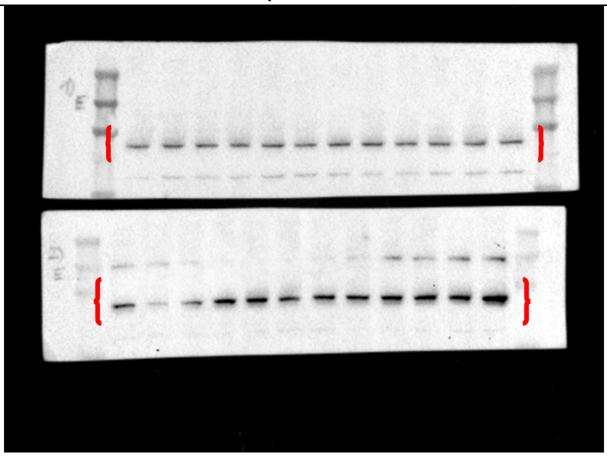 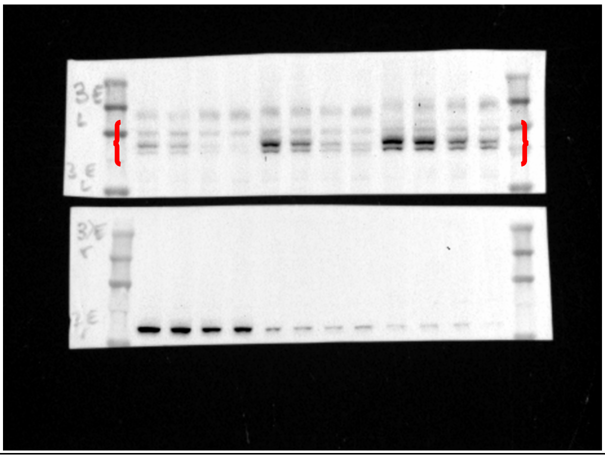
Beta-Actin	

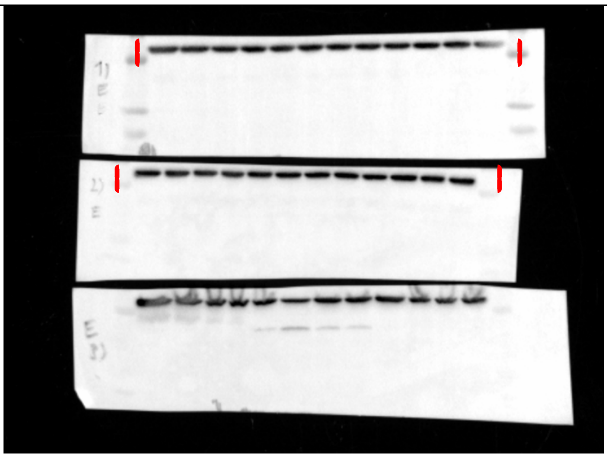

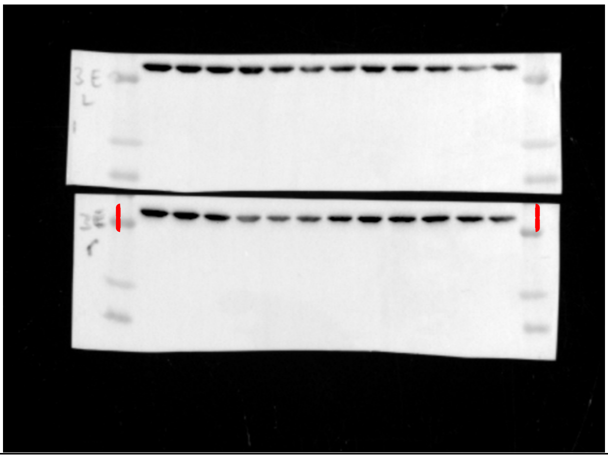

Full unedited gels for Supplemental Figure 7B

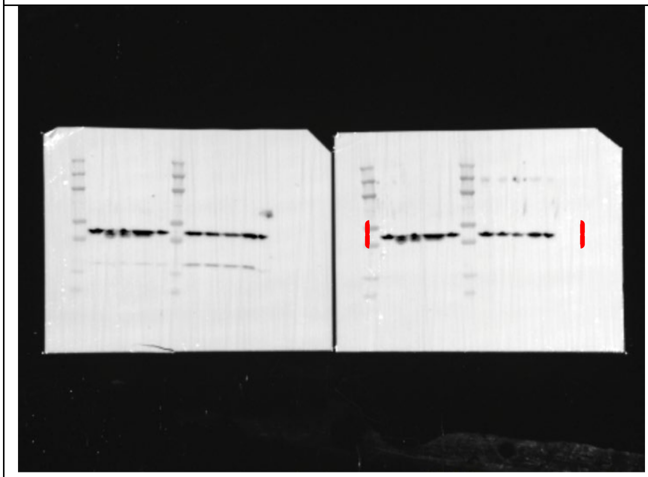	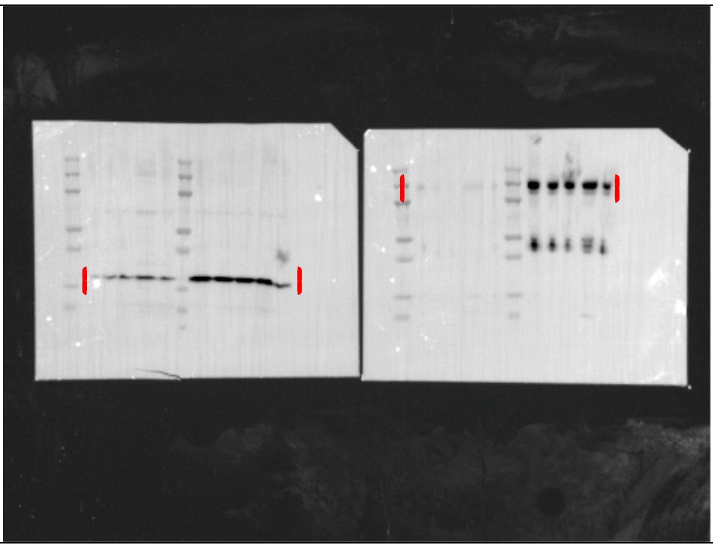

Full unedited gels for Supplemental Figure 11E

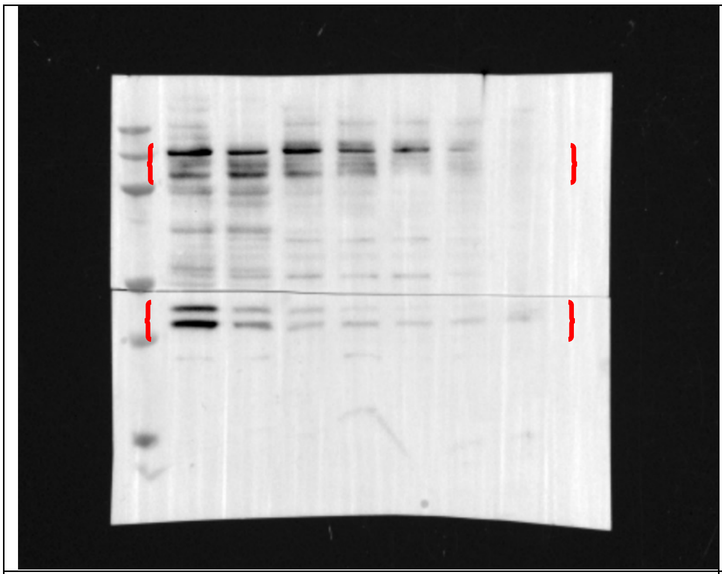

Phospho FGFR1 + Phospho ERK

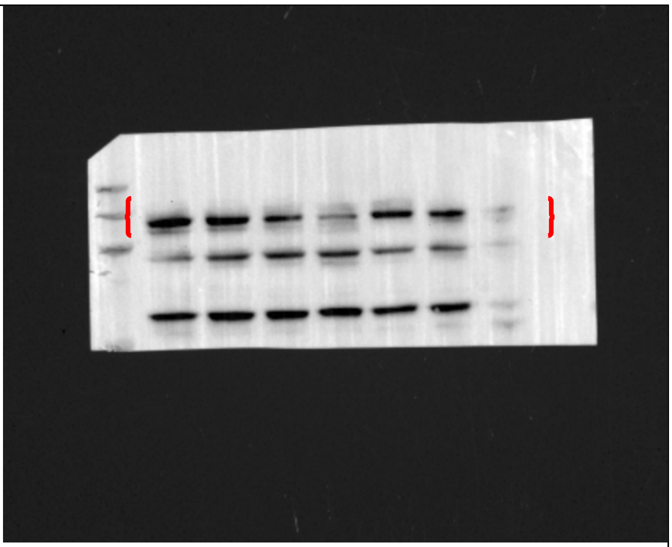

FGFR1

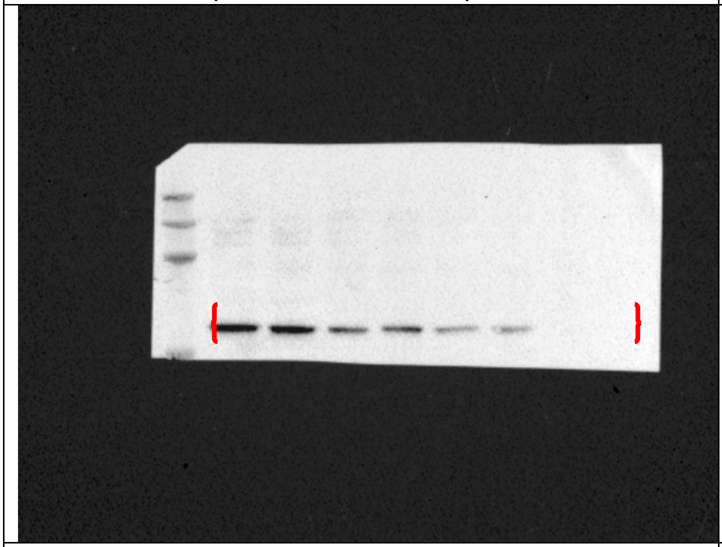

Phospho AKT

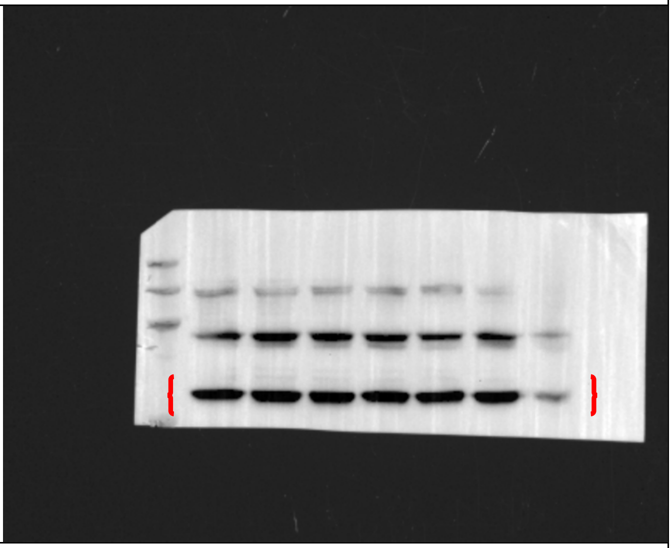

AKT

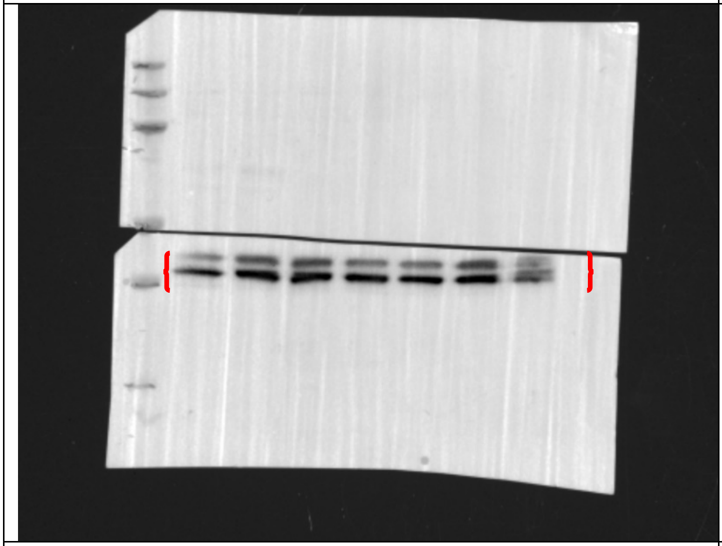

ERK

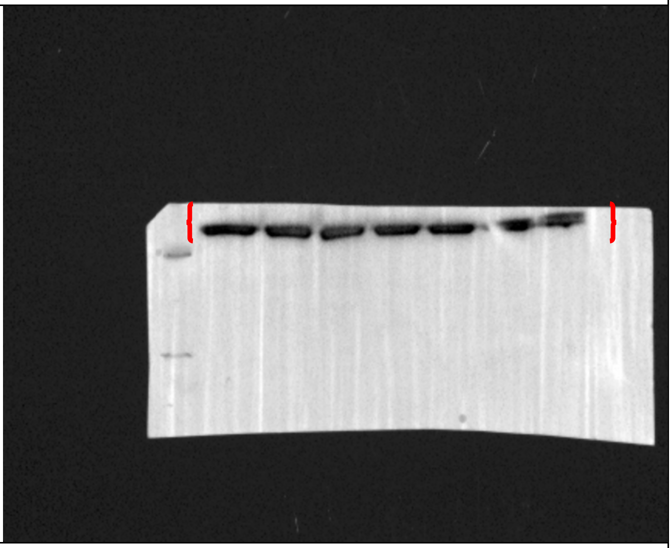

b-Actin
